# A Comparative Metagenome Survey of the Fecal Microbiota of a Breast- and a Plant-Fed Asian Elephant Reveals an Unexpectedly High Diversity of Glycoside Hydrolase Family Enzymes

**DOI:** 10.1371/journal.pone.0106707

**Published:** 2014-09-10

**Authors:** Nele Ilmberger, Simon Güllert, Joana Dannenberg, Ulrich Rabausch, Jeremy Torres, Bernd Wemheuer, Malik Alawi, Anja Poehlein, Jennifer Chow, Dimitrij Turaev, Thomas Rattei, Christel Schmeisser, Jesper Salomon, Peter B. Olsen, Rolf Daniel, Adam Grundhoff, Martin S. Borchert, Wolfgang R. Streit

**Affiliations:** 1 Universität Hamburg, Biozentrum Klein Flottbek, Abteilung für Mikrobiologie & Biotechnologie, Hamburg, Germany; 2 Georg-August-University Göttingen, Institute of Microbiology and Genetics, Göttingen, Germany; 3 University Medical Center Hamburg-Eppendorf, Bioinformatics Service Facility, Hamburg, Germany; 4 University of Vienna, CUBE - Division for Computational Systems Biology, Department of Microbiology and Ecosystem Science, Vienna, Austria; 5 Novozymes A/S, Microbial Discovery, Bagsværd, Denmark; 6 Heinrich Pette Institute, Leibniz Institute for Experimental Virology, Hamburg, Germany; Charité-University Medicine Berlin, Germany

## Abstract

A phylogenetic and metagenomic study of elephant feces samples (derived from a three-weeks-old and a six-years-old Asian elephant) was conducted in order to describe the microbiota inhabiting this large land-living animal. The microbial diversity was examined via 16S rRNA gene analysis. We generated more than 44,000 GS-FLX+454 reads for each animal. For the baby elephant, 380 operational taxonomic units (OTUs) were identified at 97% sequence identity level; in the six-years-old animal, close to 3,000 OTUs were identified, suggesting high microbial diversity in the older animal. In both animals most OTUs belonged to Bacteroidetes and Firmicutes. Additionally, for the baby elephant a high number of Proteobacteria was detected. A metagenomic sequencing approach using Illumina technology resulted in the generation of 1.1 Gbp assembled DNA in contigs with a maximum size of 0.6 Mbp. A KEGG pathway analysis suggested high metabolic diversity regarding the use of polymers and aromatic and non-aromatic compounds. In line with the high phylogenetic diversity, a surprising and not previously described biodiversity of glycoside hydrolase (GH) genes was found. Enzymes of 84 GH families were detected. Polysaccharide utilization loci (PULs), which are found in Bacteroidetes, were highly abundant in the dataset; some of these comprised cellulase genes. Furthermore the highest coverage for GH5 and GH9 family enzymes was detected for Bacteroidetes, suggesting that bacteria of this phylum are mainly responsible for the degradation of cellulose in the Asian elephant. Altogether, this study delivers insight into the biomass conversion by one of the largest plant-fed and land-living animals.

## Introduction

The microbiota of mammalian intestines is the main driver of plant cell wall degradation as mammalian genomes do not encode for a significant number of genes linked to structural polysaccharide, i.e. cellulose, degradation [1]. Thereby herbivores can gain 70% of their energy from microbial polysaccharide breakdown [1]–[3]. Within the herbivores two groups are distinguished corresponding to the location of the main fermentation: the foregut fermenters (mainly ruminants) and the hindgut fermenters. In foregut fermenters the fermentation takes place in pregastric chambers, the rumen. In hindgut fermenters the main fermentation chamber is the colon or the caecum (e.g. elephants, their caecum is up to 1.5 m long) and therein the dry matter content is significantly higher than in the rumen [4]. In addition, ruminants grind the lignocellulosic material mechanically by chewing regularly during the fermentation process. Ruminants therefore have a very effective way of digesting lignocellulose. Hindgut fermenters in contrast are able to digest faster; this is an advantage at high body size as more food can be ingested [5]. Recently, the intestinal or fecal microbiota of different animals has been investigated using next generation sequencing (NGS) technology. Among them were cow, reindeer, wallaby, yak, giant panda, buffalo, swine, Iberian lynx and termite [6]–[16]. These studies delivered a significant amount of novel sequence data giving insight into the phylogeny, the metabolism and the genetic potential of the intestinal microbiota. Furthermore, these studies have led to a better understanding of fecal and gut microbial communities and they have underlined the importance of these communities for host survival, fitness, physiology and nutrient utilization [17]–[19]. In addition these studies have suggested a core and a variable gene set, influenced by host traits, environment, type of diet and other not yet identified factors [20]–[22]. In general and as expected, the herbivorous microbiomes encoded for high numbers of carbohydrate active enzymes (CAZymes). For many of the studied systems Clostridia were most abundant and were identified as main cellulose degraders [11], [23]. Interestingly, in termites Spirochaetes and Fibrobacteres are supposed to be the main contributors to cellulose digestion [16]. For these microbes the absence of cellulosomes and the release of at least some of the cellulases in the ruminal fluid were proposed [16]. Furthermore, some Bacteroidetes have been described as being highly abundant in different fecal/intestinal samples [6], [8]–[10]. Recent metagenomic and genomic studies have highlighted the presence of polysaccharide utilization loci (PULs) from Bacteroidetes that include cellulase genes [8], [10]. PULs were primarily described as starch degradation operon composed of genes encoding proteins designated SusA to SusG. These proteins coordinately cleave carbohydrates and transport sugar into the cell [1]. PULs have been identified in different gut samples and genomes of *Bacteroides* sp. [3], [8], [24]–[26].

Adult Asian elephants can reach a weight of 5,000 kg and consume about 150 kg plant materials per day. The nourishment is composed of highly fibrous plant material, mainly grass, fruits, leaves, twigs, roots and bark. As our understanding of the microbiota inhabiting elephants is poor, we were interested in characterizing the microbial population in the feces of this large herbivorous animal. Within the current paper we deliver evidence that the microbiome of Asian elephants is highly diverse and that microbes of the Bacteroidetes phylum are presumably the main cellulose degraders. Thereby we show that elephants are generalists rather than specialists with respect to the degradation of plant biomass.

## Materials and Methods

### DNA isolation

No specific permissions were required for these activities. The study did not involve endangered or protected species. Coordinates are: Longitude/Latitude 9.941572/9.941572. Fresh feces samples derived from a six-years-old female Asian elephant (“Kandy”, *2003 in Hamburg) and from a three-weeks-old male elephant (“Assam”, *2012 in Hamburg), both living in the zoo ‘Hagenbecks Tierpark’ in Hamburg (Germany), were collected from the zoo staff directly after defecation. The samples were transported, directly and on ice, to the laboratory in Hamburg Klein Flottbek for further analysis. The elephants were not treated with antibiotics. The older Asian elephant was mainly fed with grass, hay, leaves and twigs, with additional fruits and vegetables. In contrast, the three-weeks-old male elephant was breast-fed. DNA isolation was performed with the QIAamp DNA Stool kit from Qiagen (Hilden, Germany) as described previously [27].

### Metagenome sequencing

Libraries were prepared with the NEB DNA Ultra Kit following the manufacturer’s protocol. Illumina sequencing for the six-years-old elephant was performed using a HiSeq 2000 instrument (1.5 lanes, paired-end run (2×100 bases)). For the three-weeks-old animal a HiSeq 2500 instrument (one lane, paired-end run (2×100 bases)) was used for sequencing at the HPI in Hamburg. *De novo* assembly was performed with the Velvet assembly program version 1.2.08 [28]. For the investigation of the sequences the IMG server (https://img.jgi.doe.gov/cgi-bin/mer/main.cgi) was used. To further analyze the possible biological processes linked to the individual genes and ORFs mainly the KEGG [29], the COG [30] and the Pfam [31] databases were employed using a cut off of 10^−5^.

### Amplification and sequencing of 16S rRNA genes

To assess the microbial diversity, variable regions of the 16S rRNA genes were amplified as previously published [32] but with minor modifications.

The V3–V5 region was amplified using the following primer set: V3for 5′-TCTC ATCCCTGCGTGTCTCCGACTCAGACGCTCGACACCTACGGGNGGCWGCAG-3′ and V5rev 5′-CCTATCCCCTGTGTGCCTTGGCAGTCTCAGCCGTCAATTCMTTTRAGTTT-3′. The primers contained Roche 454 pyrosequencing adaptors, keys and one unique MID per sample (underlined). To assess the archaeal diversity, the V4–V6 region was amplified using the primer set: A519F 5′-CCATCTCATCCCTGCGTGTCTCCGACT CAGATATCGCGAGCAGCMGCCGCGGAA- 3′ and A1041R 5′-CCTATCCCCTGTGT GCCTTGGCAGTCTCAGGGCCATGCACCWCCTCTC-3′. The PCR reaction (50 µl) contained 0.5 U of Phusion High-Fidelity DNA Polymerase (Thermo Scientific, Germany), 10 µl 5x Phusion GC Buffer, 200 µM of each *d*NTP, 2.5% DMSO, 1.5 mM MgCl_2_, 4 µM of each primer, and 20 ng isolated DNA. PCR cycling conditions were: initial denaturation at 98°C for 3 min, followed by 28 cycles of denaturation at 98°C for 30 s, annealing at 61°C for 30 s (archaeal primer set: 66°C), and extension at 72°C for 25 s. The final extension was conducted at 72°C for 5 min. Negative controls were performed with H_2_O instead of template DNA. The obtained PCR products were purified via Gel/PCR DNA Fragments Extraction Kit (Geneaid Biotech, Taiwan) as recommended by the manufacturer. Three separate PCR reactions were conducted for each sample. After gel extraction, the reaction products were pooled in equal amounts. The 16S rRNA gene sequencing was performed at the Göttingen Genomics Laboratory using a Roche GS-FLX+454 pyrosequencer and titanium chemistry (Roche, Branford, USA).

### Processing and analysis of 16S rRNA gene data sets

Via pyrosequencing generated raw sequences were processed according to [33], with the following modifications: After raw data extraction, reads shorter than 300 bp and those possessing long homopolymer stretches (>8 bp) or primer mismatches (>5 bp) were removed. The sequences were denoised employing Acacia [34]. Chimeric sequences were removed using UCHIME in reference mode with the most recent SILVA SSU database as reference dataset (SSURef 115 NR) [35]. The processed 16S rRNA gene sequences were uploaded to the SILVA NGS (SILVA next-generation sequencing) server for taxonomic classification [36]. Microbial taxonomy was determined using default settings with two adjustments: The cluster sequence identity threshold was decreased to 0.97 and the maximal taxonomic depth was increased to 30. Rarefaction curves were calculated employing the QIIME 1.8 software package [37].

### Comparison with other fecal metagenomes and statistical analysis

An additional set of metagenomes of animals’ microbiotas was used for comparative analysis by the IMG/M ER webpage of the US Department of Energy Joint Genome Institute (https://img.jgi.doe.gov/cgi-bin/mer/main.cgi) [38]. The phylogenetic distribution of the metagenomes was analyzed. A phylogenetic tree was automatically created with the public Metagenomics RAST server [39].

### Binning

The assembly of both metagenomic samples was performed with the Ray Meta assembler [40], version 2.3.1, using a k-mer length of 31 and default parameters. Scaffolds of ≥2 kb length and a mode k-mer coverage depth of >5 were binned based on unsupervised tetranucleotide frequencies using MetaWatt2.0 [41]. To assess bin taxonomy, homogeneity and completeness we identified bacterial and archaeal phylogenetic marker genes using AMPHORA2 [42]. Using project-specific scripts we estimated the most recent taxonomic level supported by most high-confidence AMPHORA2 markers (confidence score ≥0.9) for each bin. At these levels the phylogenetic markers determined the taxonomic affiliation and completeness of each bin. The tRNA genes, rRNA genes and protein-coding genes were predicted in each bin using RNAmmer [43], tRNAscan-SE [44] and GeneMarkS [45], respectively.

### Transmission electron microscopy (TEM)

Slices were prepared with the microtome Reichert-Jung Ultracut E. Fixation was performed in 2% glutaraldehyde in 75 mM cacodylate buffer (pH 7.0). After washing it was supplied with 2% agar in 75 mM cacodylate buffer (pH 7.0) and further fixed with 1% OsO_4_ in 50 mM cacodylate buffer (pH 7.0). After washing with 75 mM cacodylate buffer (pH 7.0) water was removed with acetone and the sample was infiltrated with Spurr resin (Polysciences, Warrington, PA, USA). TEM pictures were observed with the LEO 906 E, the camera Gatan 794 and the software Digital micrograph.

### DNA sequences obtained and GenBank submissions

This project has been deposited at GenBank using the BioProject number PRJNA240141. The sequences derived from Illumina and 454 sequencing were deposited in the NCBI Short Read Archive, the study accession number is SRP040073. Assembled sequence data with predicted gene models and annotation is available from www.jgi.doe.gov. (DOE Joint Genome Institute), the IMG Project Id is 50566.

## Results and Discussion

### Population structure of the elephant feces samples

**Figure 1:**
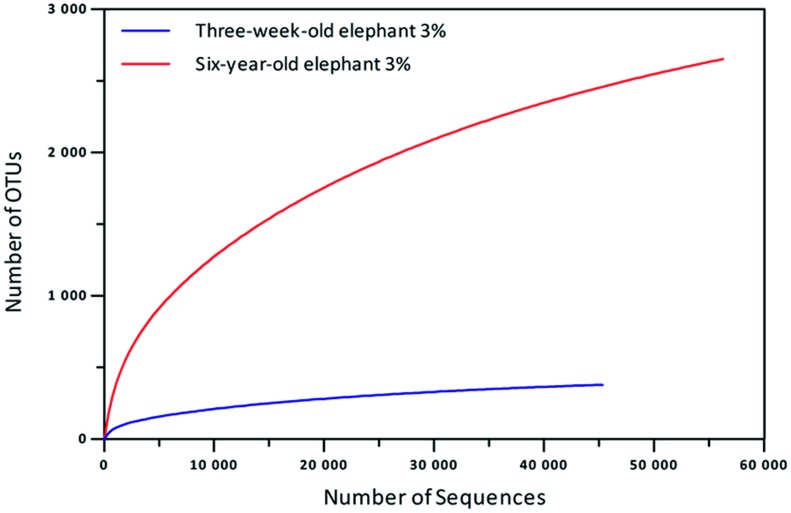
Rarefaction curves calculated for the feces sample of the three-weeks-old and the six-years-old Asian elephant at 3% genetic distance of 16S rRNA genes. The curve for the six-years-old elephant comprises 8,014 archaeal sequences which were clustered to 54 OTUs. The sequences were denoised employing Acacia. Chimeric sequences were removed using UCHIME in reference mode with the most recent SILVA SSU database as reference dataset (SSURef 115 NR).

Recent metagenomic research has demonstrated a close correlation between host diet and intestinal microbiome and it has highlighted the metabolic diversity within intestinal and fecal samples of mammals and insects [6], [8], [9], [11], [15], [16], [46]. These intriguing findings inspired us to analyze the fecal microbiota of one of the largest land-living herbivores (only the African elephant is larger than the Asian elephant), which is known to be an exception in the world of large foregut fermenters as digesting faster than the high body size would allow [5]. To estimate the diversity of the microbes and their individual tasks that meet the energy need of elephants, a detailed phylogenetic analysis using NGS technologies was performed. Thereby we analyzed the community of a plant-fed female specimen (six-years-old) and of a three-weeks-old breast-fed male animal. Both animals were living in a local zoo and they were not treated with antibiotics. For the baby elephant 44,508 partial 16S rRNA sequences with an average length of 536 bp were generated and 56,124 sequences with an average length of 523 bp for the six-years-old elephant (TABLE 1). Rarefaction curves suggested that both datasets had the required amount of sequence data to assess operational taxonomic unit (OTU) richness and that both datasets had a similar coverage of biodiversity (FIGURE 1).

**Table 1:** Overall numbers of sequences and contigs generated for the two elephant feces samples.

Parameter	Six-years-old elephant	Three-weeks-old elephant
*16S rRNA gene sequences*		
No. of GS-FLX+454 sequences	56,124	44,508
*Feces Microbiome DNA*		
No. of Illumina reads	415,784,264	200,946,703
No. of bases assembled	929,519,943	138,494,152
No. of assembled sequences	260,535	50,765
Coverage	42	73
Mean contig size (bp)	3,566	5,801
N50 (bp)	5,751	22,052
Largest contig (bp)	344,979	597,113
Gene count	1,068,385	173,134
GC mean %	43,96	47,71
Protein coding genes	1,052,245	171,134
with COG	629,485	111,920
with Pfam	818,373	136,417
with KEGG	210,308	42,640

**Figure 2:**
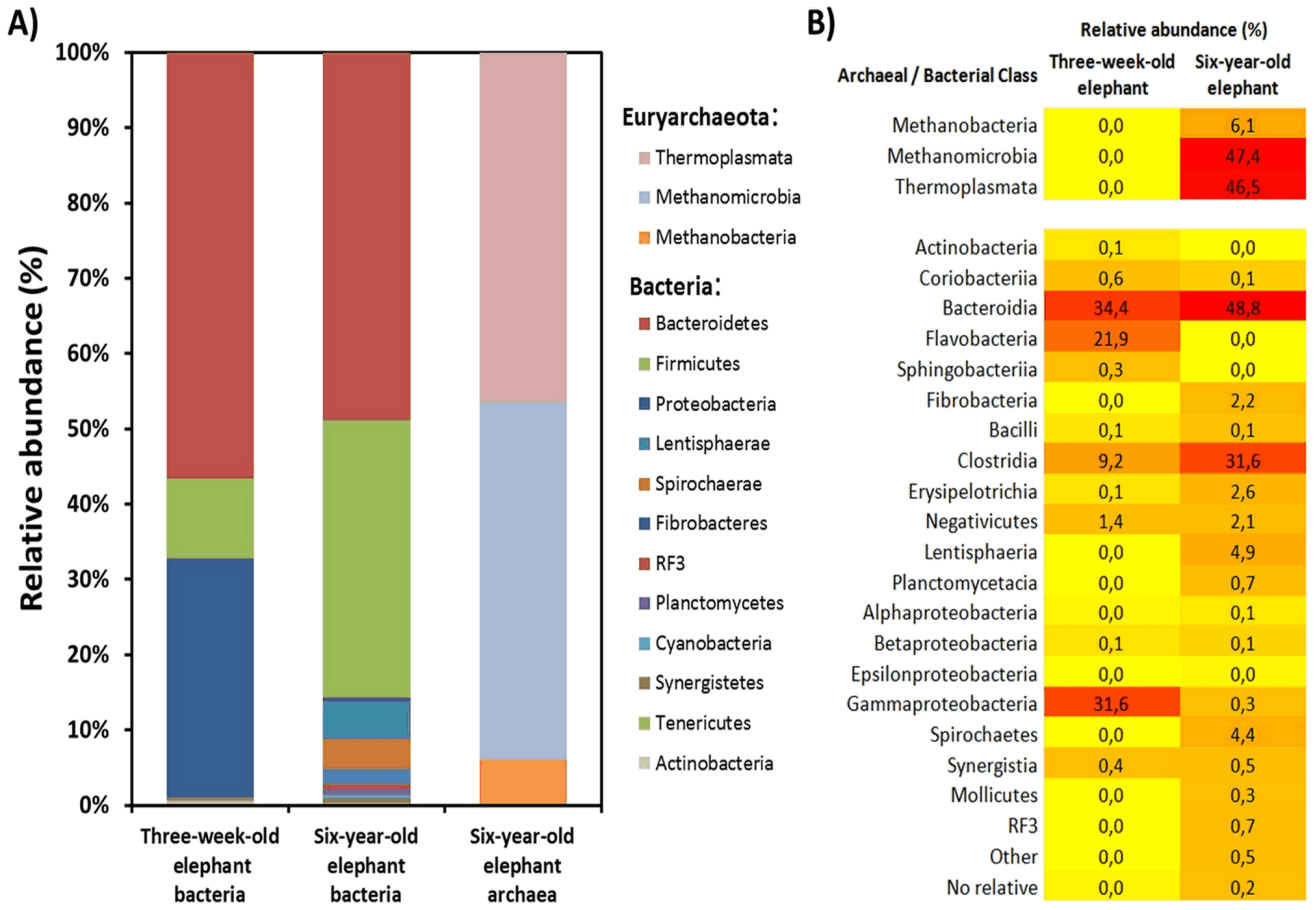
Relative abundances of different phyla and classes in the two elephant feces samples. **A:** Relative abundance of phyla in the feces of the three-weeks-old and the six-years-old Asian elephant based on 16S rRNA gene sequences. For the three-weeks-old elephant no Archaea were observed. **B:** Phylogenetic comparison on class level between both elephants. Heat map colors indicate the abundances of the respective 16S rRNA genes.

The feces sample of the six-years-old elephant revealed an almost 10-fold higher diversity than that of the baby animal (FIGURE 1, 2). For the three-weeks-old animal the phylogenetic analysis suggested the presence of approximately 380 OTUs based on a 97% sequence identity cut off for bacterial 16S rRNA genes. However, in the feces of the older animal 2,656 OTUs (calculated max. 3,487 OTUs) were identified (FIGURE 1, TABLE S1). The Shannon index for the baby elephant was 4.3 and for the six-years-old elephant it was 8.4. Furthermore the Chao1 index for species richness was 449 for the baby and 3,281 for the six-years-old elephant (TABLE S1), leading to the overall conclusion that the microbiome of the older elephant was much more diverse than that of the baby.

The baby elephant’s feces sample was dominated by bacteria belonging to the Bacteroidetes (FIGURE 2). More than 50% of the microbes were Flavobacteriales (22%) or Bacteroidales (34%). The sample of the baby elephant furthermore comprised a high number of Proteobacteria (32%) and Firmicutes (11%) (FIGURE 2). On genus level most abundant bacteria were *Myroides* (22%), the S24-7 group of Bacteroidetes (14%) and *Pseudomomas* and *Psychrobacter* species (12% each) (supporting information S1). With the archaeal primer set no PCR product was observed.

The bacterial population of the six-years-old animal was dominated by Firmicutes (36%) and Bacteroidetes (47%). Furthermore Spirochaetes (4%), Fibrobacteres (2%) and Lentisphaeria (5%) were detected. Interestingly no bacteria belonging to these phyla were detected in the three-weeks-old elephant’s sample. On genus level uncultured *Lachnospiraceae* (10%), *Ruminococcaceae* (10%), *Prevotellaceae* (11%) and the RC9 gut group of *Rickenellaceae* (13%) dominated (supporting information S2). Archaea were found in this sample. About 50% of these belonged to the Methanomicrobiales and the remainder belonged mostly to the Thermoplasmatales, i.e. Candidatus *Methanomethylophilus* (FIGURE 2). The respective organisms most likely contribute to elephant’s methane production during feed fermentation [47].

To further verify these data, we analyzed the sequences obtained by Illumina sequencing for the presence of rRNA gene fragments or complete genes. 102 16S rRNA gene fragments were found for the baby elephant and 338 16S rRNA gene fragments for the six-years-old elephant. The results from this analysis largely confirmed the results of the 16S rRNA amplicon sequencing. Additionally, binning was performed with the metagenomic sequence data. For the baby elephant 234 bins were observed, for the six-years-old elephant 1,401. Thereof 131, or 935, respectively, were unassigned (TABLE S2, S3). The results largely confirm the 16S rRNA analysis, the higher microbial richness of the six-years-old elephant and the phylogenetic affiliations. For the older elephant Firmicutes and Bacteroidetes were dominant with 39 and 41% of the bins, respectively. Spirochaetes and Fibrobacteres were present at lower levels and 6% of the bins were assigned to Archaea (TABLE S3). For the baby elephant Firmicutes (35%), Bacteroidetes (32%) and Proteobacteria (28%) were dominant (TABLE S2). This is, in comparison with the 16S rRNA analysis, an overrepresentation of Firmicutes and an underrepresentation of Bacteroidetes. This difference nevertheless can easily be explained by the different methods.

**Figure 3:**
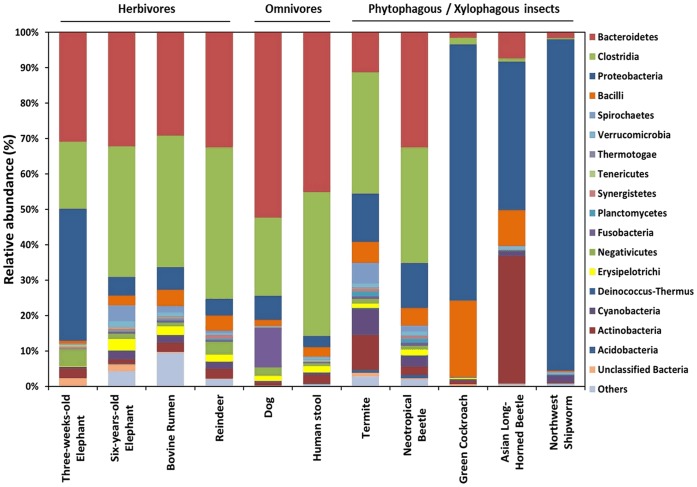
Phylogenetic analysis of the elephant feces in comparison with other fecal and intestinal metagenome data sets. Data indicate the phylogenetic relation based on gene similarities in the metagenome sequences. The percent of sequences assigned to each phylum according to IMG/M ER is shown based on the total number of obtained sequences of each data set. Sequence data for the metagenomes were extracted from the IMG/M ER web page of the US Department of Energy Joint Genome Institute and the respective bioprojects (IMG Genome IDs: Six-years-old Elephant (this study): 3300001598; three-weeks-old Elephant (this study): 3300001919; Green Cockroach: 2228664000; Termite: 3300001544; Dog: 2019105001; Reindeer: 2088090000; Neotropical Beetle: 3300000114; Asian Long-Horned Beetle: 2084038013; Bovine Rumen: 2061766007; Northwest Shipworm: 2189573029; Human stool: 7000000038).

Overall and as expected the microbiota of the six-years-old elephant resembled those of most herbivorous mammals like reindeer, wallaby and cow [6], [8], [9] with Firmicutes, Bacteroidetes and Proteobacteria being dominant (FIGURE 3). Surprisingly, the number of OTUs was at least two-fold higher than those of other herbivores (FIGURE 3; TABLE 2 and references given herein). Since Asian elephants have a rather diverse diet in the zoo compared to wild living animals this relatively high bacterial diversity may reflect an adaptation to this feeding manner. Furthermore the nourishment is composed of different plants and the microbiome is limited to the small zoo population. Thus, the results altogether do not represent the microbiome of wild living animals. It has been shown e.g. for pandas that the communities of wild and captive animals differ greatly [11].

**Table 2:** Selected and recent metagenome studies published from insect or mammalian fecal and gut samples.

Microbiome	OTUs detected	Assemble DNA (Mbp)	Sequencing strategy	No. of GH families identified	Total no. of CAZymes	No. of GH per Mbp assembled DNA	^f)^Total no. of cellulolytic enzymes	Reference
Iberian lynx	n.d.	18.3	454 GS FLX	42	372	20.3	39	[15]
Tammar wallaby	236	85.2	454 GS FLX	53	557	6.5	175	[9]
Reindeer^a^	1,182	26	454 GS FLX	30	^d)^5,000	^e)^9.9	^d)^400	[8]
Giant panda	85	37	Illumina	44	440	11.9	124	[11]
Bovine	1,000	^b)^1,930	Illumina	60	27,755	14.4	5,670	[36]
Bovine	161–259	^c)^104	454 GS20	35	3,828	^c)^36.8	1,017	[7]
Termite	216	71	454 GS20+ Sanger	45	703	9.9	259	[16]
Elephant gut (six-years-old)	2,656	929.5	Illumina	82	11,038	11.9	2,074	This study
Elephant gut(three-weeks-old)	380	138.5	Illumina	70	1,873	13.5	242	This study

n.d., not determined; a), in 2 samples; b), 286 Gb resulted in approx. 2 Gb of assembled DNAs >1 kb scaffolds; c),unassembled; d), in unassembled reads; e), per 503 Mbp unassembled DNA; f), total gene count with respect to the GH1, GH3, GH 5, GH6, GH8, GH9, GH44, GH45, GH48, GH51, GH74, GH94 family enzymes. Total gene counts, size of assembled DNAs, OTUs and numbers of carbohydrate active enzymes were extracted from the indicated references.

As the baby elephant was breast-fed, the microbes inhabiting the intestine of this animal were in part determined by the mother’s milk and of those bacteria attached to the mother transmitted by direct contact [48]. We speculate that especially the relatively high level of Proteobacteria was a result of the breast-feeding (FIGURE 3). This observation fitted well with the fact that in human breast milk more than 60% of the bacteria were Proteobacteria [49]. While the observed differences in OTU richness of the microbial communities of both elephants can most likely be explained by the differing nourishment, it should be noted that baby elephants also eat feces from older animals. Because of this it is likely that a significant fraction of the microbes in its feces originated from other animals from the herd.

Nevertheless, only 13 OTUs were present in both elephants’ microbiomes. These were seven Firmicutes, three Bacteroidetes and three Proteobacteria (TABLE S4). Interestingly, most of these OTUs were considerably higher represented in one of the samples (TABLE S4). Altogether the two communities are distinct with only few overlaps. Though of course analyzing only two individuals, these data give hint to the great changes the intestinal microbiota is subjected to during animal’s development.

### Metagenome survey reveals remarkable metabolic richness

In this study metagenomic sequence data was generated for the intestinal microbiota of a six-years-old Asian elephant and of a three-weeks-old baby elephant. The reads observed from Illumina sequencing were assembled to 929,519,943 bp in 260,535 contigs for the six-years-old animal. The largest contig was 344,979 bp. The average contig length was 3,566 bp (TABLE 1). For the baby elephant 138,494,152 bp were assembled. The average contig length was 2,728 bp. The largest contig was 597,113 bp (TABLE 1).

To broadly characterize the sequence data, general traits were analyzed. Approximately 1,052,245 protein-coding genes could be identified for the-six-years-old elephant and 171,134 for the baby elephant. Of these, a total of 210,308 for the former and 42,640 for the latter were similar to putative proteins in the KEGG database (TABLE 1). A small fraction (1.0/0.6% respectively) of the putative proteins was derived from eukaryotes. Although the available sequences do not allow a complete analysis, when taking the coverage of the obtained sequences into account, the 1.1 Gbp assembled DNA represented a significant fraction of the metagenome and the sequences therefore gave a good first estimation of the communities’ metabolic potential. The amount of assembled sequence data was in general significantly higher than in similar studies (TABLE 2). Only for the cow rumen metagenome a two-fold higher amount of assembled sequence data was reported [6]. Furthermore the N50 of both datasets (5,751 and 22,052 bp, for the baby and the six-years-old elephant, respectively) were average or high in comparison with similar studies. E.g. in a study with Iberian Lynx the N50 was 4,370 bp [15] and for a study with bovine rumen it was 24 kb [6].

The KEGG analysis suggested that the metabolic potential of the microbiomes of the fecal samples is highly diverse and versatile. Genes of many of the classical catabolic pathways linked to the degradation of diverse polysaccharides and proteins, but also for the degradation of aromatic compounds, were identified. In both samples about 25% of the putative proteins were involved in carbohydrate metabolism and glycan biosynthesis (data not shown). A high number of genes coding for proteases (9,998 and 1,396 for the six-years-old and the three-weeks-old animal, respectively) were observed. Further 6,657/843 genes encoding putative esterolytic and/or lipolytic enzymes were detected, suggesting high hydrolytic activity for esters of fatty acids within both microbial communities.

### Unique GH diversity in the fecal samples

Since the older elephant was fed on a large variety of different plant-based polysaccharides we focused on the analysis of genes and enzymes linked to carbohydrate catabolism. For a more detailed analysis we compared our set of predicted CAZymes (carbohydrate active enzymes) to entries in the CAZy database. The CAZy database contains a large set of validated CAZymes and it provides a sequence-based family classification of enzymes that are involved in the modification and breakdown of polysaccharides [50]. One of the most striking findings was the observation that the metagenomes of both elephants exhibited extraordinary high diversity of GHs when compared to other fecal samples of herbivores (TABLE 2). Our analyses identified a total of 11,038 putative genes for CAZymes from 82 different GH families in the six-years-old elephant’s microbiome (TABLE 2). In the three-weeks-old elephant we detected 1,873 GH genes from 70 GH families. The majority of the identified GH genes were predicted to represent full-length genes. Furthermore, more than 50% of the enzymes showed less than 50% identity to the nearest neighbor (data not shown).

Interestingly, the diversity of GH family enzymes observed in both samples in this study was in general 2-fold higher than reported for any of the previously studied samples (TABLE 2) and with respect to the study on the cow rumen the GH diversity still was 20% higher [6]. This observation is in line with the observed higher phylogenetic diversity in the feces sample of the older elephant. When comparing GH diversity and OTU richness for the baby elephant, a very high GH diversity was observed (TABLE 2). Within the older elephant’s fecal metagenome enzymes belonging to GH2, GH3, GH5, GH43 and GH78 families were predominant. Altogether 4,039 genes were linked to these five GH families, being equivalent to one third of all observed CAZymes in this study (TABLE 3). With respect to the overall occurrence of GH2, GH3, GH5 and GH43 family enzymes this observation fitted well with reports on other herbivores such as cow rumen, reindeer and others summarized in TABLE 2. GH2 and GH3 family enzymes encompass ß-galactosidases, ß-glucosidases, exoglucanases but also xylosidases. These enzymes are involved in the breakdown of a large variety of oligosaccharides. GH5 enzymes are endoglucanases involved in cellulose breakdown. Similar to microbiota of other herbivores the six-years-old elephant’s data set contained a large number of putative genes matching enzymes of GH families specific for the metabolism of xylo-oligosaccharides (TABLE 3). The most abundant were GH10 mainly acting as ß-1,4-xylanases and the GH43 family enzymes acting mainly as ß-xylosidases (TABLE 3).

**Table 3:** Gene count, relative gene count and relative coverage of genes for families of carbohydrate-active enzymes discovered in the sequences of the elephant feces samples according to CAZy.

	Baby elephant			Six-years-old elephant		
1)GH family	Gene count	Relative count	Relative coverage	Gene count	Relative count	Relative coverage
**1**	33	0.01761	0.00979	103	0.00933	0.00667
2	220	0.11745	0.09455	917	0.08311	0.082006
**3**	148	0.07901	0.08355	804	0.07287	0.07784
4	16	0.00854	0.00430	57	0.00517	0.00405
**5**	19	0.0101	0.01064	517	0.04686	0.04808
**8**	4	0.00213	0.00134	85	0.00770	0.01198
**9**	9	0.00481	0.00406	119	0.01078	0.01199
10	6	0.00320	0.00836	258	0.02338	0.02915
11	–	–	–	20	0.00181	0.00142
13	124	0.06620	0.07574	845	0.07658	0.07423
15	1	0.00053	0.00019	1	0.00009	0.00008
16	22	0.01174	0.01399	198	0.01794	0.01552
17	–	–	–	5	0.00045	0.00022
18	51	0.02723	0.03378	86	0.00779	0.00824
19	1	0.00053	0.00150	3	0.00027	0.00011
20	108	0.05766	0.05088	266	0.02411	0.01881
23	69	0.03684	0.06012	294	0.02664	0.03324
24	12	0.00641	0.00685	26	0.00236	0.00191
25	30	0.01602	0.03030	229	0.02075	0.02255
26	8	0.00427	0.00341	103	0.00933	0.00931
27	12	0.00641	0.00591	158	0.01432	0.01366
28	26	0.01388	0.01502	242	0.02193	0.02112
29	79	0.04218	0.04032	376	0.03408	0.02950
**30**	17	0.00908	0.00727	95	0.00861	0.00975
31	35	0.01869	0.01000	318	0.02882	0.02962
32	21	0.01121	0.00879	119	0.01078	0.01409
33	31	0.01655	0.01639	149	0.01350	0.01003
35	36	0.01922	0.01745	123	0.01115	0.01155
36	32	0.01708	0.01637	310	0.02809	0.02396
37	3	0.00160	0.00112	17	0.00154	0.00144
38	12	0.00641	0.00653	81	0.00734	0.00645
39	3	0.00160	0.00374	89	0.00807	0.00554
42	9	0.00481	0.00262	37	0.00335	0.00247
43	98	0.05232	0.03978	894	0.08102	0.09836
**44**	–	–	–	7	0.00063	0.00116
**45**	–	–	–	7	0.00063	0.00084
46	–	–	–	1	0.00009	0.00004
50	2	0.00107	0.00343	26	0.00236	0.00184
**51**	24	0.01281	0.00653	239	0.02166	0.02139
53	6	0.00320	0.00632	88	0.00798	0.00976
54	–	–	–	13	0.00118	0.00136
55	2	0.00107	0.00030	8	0.00073	0.00052
57	11	0.00587	0.00893	115	0.01042	0.00941
63	7	0.00374	0.00434	57	0.00517	0.00318
64	–	–	–	2	0.00018	0.00008
65	7	0.00374	0.00574	39	0.00353	0.00285
66	3	0.00160	0.00068	10	0.00091	0.00069
70	–	–	–	1	0.00009	0.00002
73	36	0.01922	0.01851	202	0.01831	0.01721
**74**	–	–	–	11	0.00100	0.00084
76	12	0.00641	0.00510	9	0.00082	0.00081
77	28	0.01495	0.01611	193	0.01749	0.01614
78	41	0.02189	0.03159	413	0.03743	0.02897
79	3	0.00160	0.00264	–	–	–
81	–	–	–	3	0.00027	0.00009
84	9	0.00481	0.00250	19	0.00172	0.00165
88	33	0.01762	0.01190	54	0.00489	0.00342
92	79	0.042178	0.04649	213	0.01930	0.01900
93	–	–	–	2	0.00018	0.00009
**94**	5	0.00267	0.00140	89	0.00807	0.00906
95	47	0.02509	0.01727	164	0.01486	0.01587
97	44	0.02349	0.02573	197	0.01785	0.02091
98	1	0.00053	0.00021	23	0.00208	0.00330
99	–	–	–	2	0.00018	0.00012
102	3	0.00160	0.00084	4	0.00036	0.00010
103	3	0.00160	0.01026	2	0.00018	0.00005
104	4	0.00214	0.00193	–	–	–
105	25	0.01335	0.00548	126	0.01142	0.01231
106	13	0.00694	0.01614	97	0.00879	0.00832
108	6	0.00320	0.00165	14	0.00127	0.00081
109	22	0.01175	0.01299	125	0.01133	0.00876
110	15	0.00801	0.00812	30	0.00272	0.00237
113	–	–	–	11	0.00100	0.00036
115	7	0.00374	0.00223	74	0.00671	0.00891
116	8	0.00427	0.00147	26	0.00236	0.00139
117	4	0.00214	0.00079	9	0.00082	0.00055
120	6	0.00320	0.00197	45	0.00408	0.00468
123	10	0.00534	0.00288	46	0.00417	0.00276
125	17	0.00908	0.01145	35	0.00317	0.00313
126	–	–	–	2	0.00018	0.00004
127	11	0.00587	0.00802	88	0.00798	0.00639
128	1	0.00053	0.00298	15	0.00136	0.00121
129	1	0.00053	0.00024	7	0.00063	0.00034
130	22	0.01175	0.01020	127	0.01151	0.01197

1) GH families according to the CAZy database http://www.cazy.org; Searches for glycoside hydrolases were performed with pfam HMMs, named in accordance with the CAZy nomenclature scheme. - no count observed, GH families not listed were not detected in any of the samples; the relative counts indicate the relative number in comparison to all GHs in the respective sample, and the relative coverage indicates the respective coverage in relation to the coverage of all GHs in the respective sample; GH families with cellulases are in bold.

Further, we observed a high number of GH78 and GH13 family enzymes. GH78 family enzymes are mainly α-L-rhamnosidases and involved in rhamnose removal from polysaccharides or other molecules including polyphenols. GH78 has only been reported to be predominant in the cow rumen metagenome [6]. Since GH13 family enzymes are mainly involved in starch breakdown the relatively high number of GH13 enzymes suggests that starch depolymerization plays a major role for nutrient uptake in the elephant’s intestinal tract. This is likely since the elephants are also fed with fruits and vegetables containing high amounts of starch.

With respect to those enzymes involved in cellulose breakdown our data analysis suggested that within the feces microbiome of the six-years-old animal at least 10 GH families can be identified which are partially involved in cellulose hydrolysis (e.g. GH1, GH3, GH5, GH8, GH9, GH44, GH45, GH51, GH74 and GH94). The predominant ones were GH5 (517) and GH9 (119) cellulases and endoglucanases. Further, 239 GH51 endoglucanases/arabinofuranosidases could be identified (TABLE 3). A total number of 84 GH94 enzymes were found. GH94 enzymes are acting as cellobiose or cellodextrin phosphorylases. Thus altogether 2,074 candidate enzymes were identified to be involved in cellulose degradation in the feces sample of the six-years-old elephant and 242 in the feces of the three-weeks-old elephant, respectively.

The overall occurrence of cellulolytic GH genes per assembled Mbp of metagenome DNA in the elephant feces sample was comparable to those numbers reported for the cow rumen and the reindeer [6], [8] (TABLE 2).

The observation that the baby elephant microbiome already encoded a large GH diversity was intriguing. It however may suggest that the mother transfers already very early a part of its microbiome to the baby. This is in line with earlier reports [48]. Baby elephants occasionally eat feces from older elephants what might explain the relatively high content of GH family enzymes in the three-weeks-old animal. Nevertheless, the majority of GH family enzymes in the three-weeks-old elephant were ß-galactosidases (GH2). The rather frequent occurrence of many GH2 family enzymes is most likely linked to the breast-feeding and the high contents of lactose in the mothers’ milk.

### Bacteroidales constitute the main cellulolytic organisms within the elephant feces microbiome

**Figure 4:**
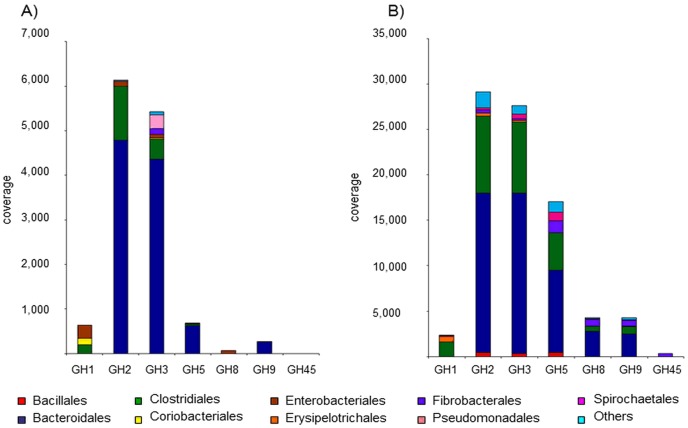
Overall coverage of selected cellulolytic GH family genes in the feces samples in relation to their phylogenetic affiliation. **A**) three-weeks-old elephant, **B**) six-years-old elephant.

**Figure 5:**
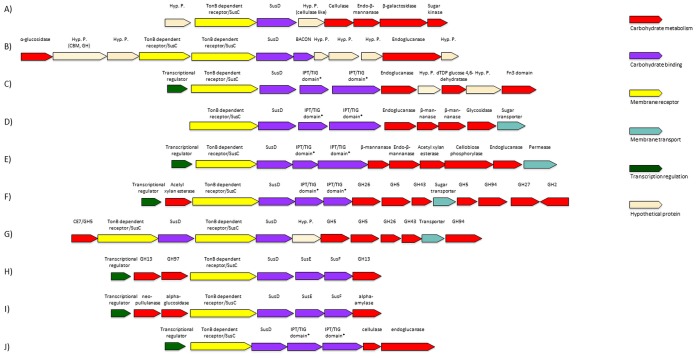
Physical map of selected putative polysaccharide utilization loci (PULs). **A)–E)** show regions from the sequence data derived from the microbiome of the six-years-old elephant. The different contigs are available from www.jgi.doe.gov (DOE Joint Genome Institute) under the IMG Project Id: 50566 with the scaffold id numbers: EMG_10007792, EMG_10000304, EMG_10002947, EMG_10003848 and EMG_10000174. **F–J)** indicate PULs from the literature or databases. **F)** from Tammar wallaby foregut [9], **G)** from Svalbard reindeer rumen [8], [21], **H)** *Bacteroides thetaiotaomicron* [3], **I)** *B. faecis* CAG:32 (GenBank: FR891562.1), **J)** *Prevotella* sp. Sc00026 (GenBank JX424618.1). *indicates IPT/TIG domain containing hypothetical proteins which have also been annotated as SusE and SusF proteins.

Interestingly, when considering protein sequence identities, the GH5, GH9 and other GH families associated with cellulolytic enzyme activities belonged in majority to Bacteroidales (FIGURE 4). In both elephants most of these sequences belonged to *Bacteroidaceae*, followed by *Lachnospiraceae* for the older and *Porphyromonadaceae* for the baby elephant (data not shown). This partially differs from other herbivores such as cow rumen in which cellulose degrading enzymes mainly derived from *Clostridium* or *Ruminococcus* species [1]. Nevertheless, a significant role of Bacteroidetes in carbohydrate degradation was already suggested for the intestines of other animals like wallaby and reindeer [8], [9]. Furthermore electron microscopic examinations confirmed the absence of significant numbers of cellulosome-producing bacteria in the elephants’ fecal samples (data not shown). Instead small coccoid cells were identified in close proximity to plant cell walls (FIGURE S1). Furthermore searches within the metagenome data set failed to identify high numbers of cellulosome associated proteins. For the older elephant 4 genes encoding a protein with a dockerin and a GH domain were discovered, for the baby elephant none. Because of these observations and the high coverage of GH5 and GH9 genes for Bacteroidales (FIGURE 4) we speculate that these are the main cellulose degraders in the elephant gut. Members of this phylum are known to degrade a variety of carbohydrates but only rarely cellulose [3]. They are well known to degrade starch and other carbohydrates with proteins encoded in an operon designated as PUL (polysaccharide utilization locus) typically containing the genes *susA* to *susG*. We identified 1,383 putative *susD* genes for the six-years-old elephant and 733 for the breast-fed animal. Interestingly, about 25 putative *sus* operons were identified which included a putative cellulase gene suggesting a possible role of PULs during cellulose breakdown. These clusters had a size from 14 kb to 25 kb. Next to cellulases these operons included a variety of different glycoside hydrolases like mannanases, galactosidases and arabinosidases indicating the flexibility of Bacteroidetes. PULs have been identified in a growing number of herbivore microbiomes and in aquatic environments [8], [9], [51]. The observation here, however, that 1,383 *susD* homologues (733 for the baby elephant) were identified suggests a high importance of these for polysaccharide degradation. Some of the clusters comprising a *susD* gene are exemplarily shown in FIGURE 5. When comparing these to clusters from other feces samples or those deriving from single strains it becomes obvious that most of the operons show high synteny (FIGURE 5). Interestingly, the operon organization in the elephant derived clusters resembles *Prevotella* species rather than *Bacteroides faecis* or the model organism *B. thetaiotaomicron* (FIGURE 5).

Besides the Bacteroidetes, the Fibrobacteres show high abundance of GH5 genes with more than 8% for the six-years-old elephant (FIGURE 4), compared to rather low abundance on phylogenetic level (2%). In contrast to the six-years-old elephant the baby elephant did not show any residing Spirochaetes or Fibrobacteres. Microorganisms of both phyla are known for their ability to digest lignocellulose and to contribute to this task in termites [16]. *Fibrobacter succinogenes* has been described as cellulolytic bacterium able to grow on crystalline cellulose. Nevertheless its mode of cellulose degradation is not elucidated. It does not produce multiprotein complexes like some *Ruminococcus* or *Clostridium* species or secrete cellulases in the surrounding medium [52]. *F. succinogenes* instead produces cellulose-binding proteins that putatively mediate close proximity of the bacterium to the substrate [46]. Therefore and as a relatively high numbers of GH5 genes have high sequence similarity to Fibrobacteres it is likely that they also play a significant role in cellulose degradation in the elephant gut.

Similar to other samples like the termite gut microbiome [16] and the cow rumen [6], exoglucanase genes were absent, suggesting that these plant-cell-wall (GH6) and *Clostridium*-derived (GH48) cellulolytic enzymes play virtually no role during cellulose degradation in the elephant feces microbiome.

### Concluding remarks

Elephants are the largest land-living animals, but our knowledge on their microbiome is limited. The data presented here give a first insight into the phylogeny of the elephant gut microbiome (i.e. fecal samples). The phylogenetic analysis of the feces of the six-years-old Asian elephant indicated that these animals host a very diverse community dominated by Bacteroidetes and Firmicutes (approx. 3,000 OTUs at 97% identity level). Furthermore, metagenome sequencing revealed a very high GH diversity of altogether 84 families. Bacteroidetes thereby seem to have a predominant role in biomass degradation. Altogether these findings distinguish the elephant feces microbiota from that of other animals like cow (Clostridiales and *Prevotellaceae* predominant) or termite (Spirochetes and Fibrobacteres predominant). Thus elephants do have a unique and flexible microbiome that meets their high energy need and allows them to digest a wide range of plant-based biopolymers. In this context it is possible to speculate that the elephant is rather a generalist and not a specialist regarding the breakdown of plant biomass. This speculation is in line with the nutrition of the six-years-old animal, which is based on leaves, twigs, hay, grass, vegetables and fruits. While both studied animals were zoo animals, in nature elephants also nurture from a wide variety of plant-derived biomass.

## Supporting Information

Figure S1 **TEM picture of a typical cell found in the feces of the six-years-old Asian elephant.**
(TIF)

Table S1 **Description of diversity and richness of the fecal samples of the three-weeks-old and the six-years-old Asian elephant based on 16S rRNA gene analysis.** The data for the six-years-old includes Bacteria and Archaea, the data for the three-weeks-old elephant only Bacteria.
(DOCX)

Table S2 **Bins observed for the baby elephant.**
(DOCX)

Table S3 **Bins observed for the six-years-old elephant.**
(DOCX)

Table S4 **OTUs observed in the 16S rRNA gene datasets of the six-years-old and the three-weeks-old elephant and their frequencies.**
(DOCX)

Supporting Information S1 **16S rRNA gene analysis of the baby elephant.**
(HTML)

Supporting Information S2 **16S rRNA gene analysis of the six-years-old elephant.**
(HTML)
