# Supplementary figures and images for: A Comparative Metagenome Survey of the Fecal Microbiota of a Breast- and a Plant-Fed Asian Elephant Reveals an Unexpectedly High Diversity of Glycoside Hydrolase Family Enzymes
Source: PLoS One. 2014 Sep 10;9(9):e106707. doi: 10.1371/journal.pone.0106707 (PMC4160196; doi:10.1371/journal.pone.0106707)

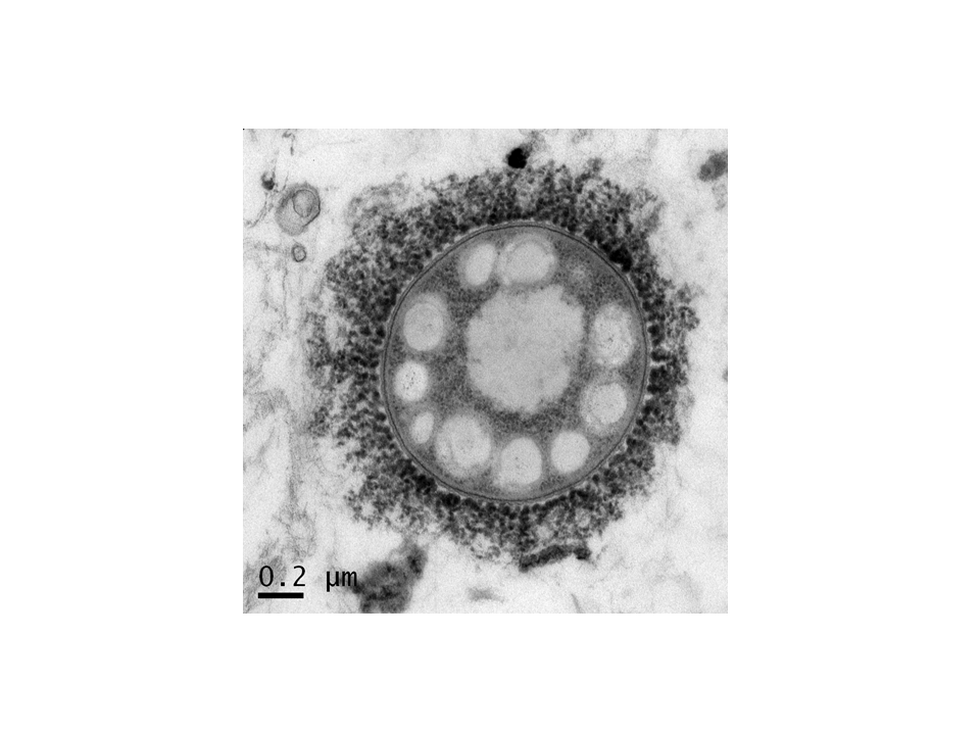

Supplement: Figure S1 — TEM picture of a typical cell found in the feces of the six-years-old Asian elephant. (TIF) [file pone.0106707.s001.tif]
