# Supplementary material for: A Comparative Metagenome Survey of the Fecal Microbiota of a Breast- and a Plant-Fed Asian Elephant Reveals an Unexpectedly High Diversity of Glycoside Hydrolase Family Enzymes
Source: PLoS One. 2014 Sep 10;9(9):e106707. doi: 10.1371/journal.pone.0106707 (PMC4160196; doi:10.1371/journal.pone.0106707)
Supplement: Table S1 — Description of diversity and richness of the fecal samples of the three-weeks-old and the six-years-old Asian elephant based on 16S rRNA gene analysis. The data for the six-years-old includes Bacteria and Archaea, the data for the three-weeks-old elephant only Bacteria. (DOCX) [file pone.0106707.s002.docx]

**TABLE S1:** Description of diversity and richness of the fecal samples of the three-weeks-old and the six-years-old Asian elephant based on 16S rRNA gene analysis. The data for the six-years-old includes Bacteria and Archaea, the data for the three-weeks-old elephant only Bacteria.

| **Sample** | **Observed OTUs** | **Calculated max. OTUs** | **Coverage (%)** | **Shannon index (H')** | **Chao1** |
| --- | --- | --- | --- | --- | --- |
| Six-years-old elephant | 2,656 | 3,487 | 76 | 8.4 | 3,281 |
| Three-weeks-old elephant | 380 | 473 | 80 | 4.3 | 449 |
