# Supplementary material for: A Comparative Metagenome Survey of the Fecal Microbiota of a Breast- and a Plant-Fed Asian Elephant Reveals an Unexpectedly High Diversity of Glycoside Hydrolase Family Enzymes
Source: PLoS One. 2014 Sep 10;9(9):e106707. doi: 10.1371/journal.pone.0106707 (PMC4160196; doi:10.1371/journal.pone.0106707)
Supplement: Table S2 — Bins observed for the baby elephant. (DOCX) [file pone.0106707.s003.docx]

Table S2: Bins observed for the baby elephant.

| Bin id | Number of scaffolds | Nucleotides | Average coverage | Taxonomic assignment | Supporting/all markers | Average marker copy number | Estimated completeness | tRNAs | rRNAs 5s/rRNAs 16s/rRNAs 23s | Number of genes |
| --- | --- | --- | --- | --- | --- | --- | --- | --- | --- | --- |
| 1 | 48 | 4417101 | 66.9+-4.2 | Odoribacter | 31/33 | 1.0 | 1.00 | 53 | 6/2/1 | 3683 |
| 2 | 34 | 6838402 | 147.7+-54.5 | Bacteroides | 51/52 | 1.6 | 1.00 | 86 | 8/0/0 | 5561 |
| 3 | 60 | 1942938 | 16.7+-1.2 | Acidaminococcus | 32/32 | 1.1 | .96 | 49 | 3/0/1 | 1806 |
| 4 | 102 | 4114179 | 65.3+-8.9 | Clostridiales | 30/30 | 1.0 | .96 | 35 | 0/0/0 | 4435 |
| 5 | 46 | 3501647 | 436.3+-69.3 | Flavobacterium | 28/31 | 1.0 | .90 | 65 | 5/0/0 | 3023 |
| 6 | 41 | 4095848 | 36.7+-9.0 | Bacteroides | 29/29 | 1.0 | .90 | 59 | 4/0/0 | 3363 |
| 7 | 53 | 1958382 | 204.7+-26.0 | Clostridiales | 28/28 | 1.0 | .90 | 22 | 0/0/0 | 1976 |
| 8 | 10 | 2807151 | 38.9+-4.8 | Coriobacterium | 39/48 | 1.5 | .83 | 72 | 3/0/0 | 2431 |
| 9 | 58 | 1892504 | 25.1+-4.2 | Ruminococcaceae | 21/26 | 1.1 | .64 | 31 | 1/1/1 | 1887 |
| 10 | 41 | 1291403 | 15.2+-1.0 | Clostridiales | 20/24 | 1.0 | .64 | 32 | 0/0/0 | 1352 |
| 11 | 63 | 3486626 | 26.1+-11.5 | Parabacteroides | 19/24 | 1.0 | .61 | 32 | 4/0/0 | 2819 |
| 12 | 32 | 1697064 | 30.1+-0.7 | Veillonella | 19/26 | 1.0 | .61 | 38 | 2/0/0 | 1591 |
| 13 | 39 | 4492362 | 41.6+-8.8 | Parabacteroides | 17/19 | 1.0 | .54 | 77 | 2/0/0 | 3580 |
| 14 | 21 | 3366039 | 94.0+-10.6 | Bacteroidales | 18/29 | 1.1 | .54 | 39 | 2/1/1 | 2783 |
| 15 | 601 | 5837111 | 429.3+-402.7 | Psychrobacter | 32/33 | 2.0 | .51 | 69 | 1/0/0 | 5813 |
| 16 | 17 | 1163258 | 55.1+-4.1 | Prevotella | 16/26 | 1.0 | .51 | 35 | 0/0/0 | 1024 |
| 17 | 16 | 81441 | 15.3+-7.0 | Pseudomonas | 20/21 | 1.2 | .51 | 7 | 0/0/0 | 116 |
| 18 | 28 | 2445681 | 809.9+-66.1 | Bacteroidales | 16/29 | 1.0 | .51 | 35 | 2/0/0 | 2025 |
| 19 | 56 | 477711 | 18.2+-5.2 | Clostridiales | 16/16 | 1.0 | .51 | 8 | 0/0/0 | 570 |
| 20 | 66 | 1684396 | 48.5+-3.5 | Ruminococcaceae | 16/16 | 1.1 | .48 | 18 | 0/0/0 | 1684 |
| 21 | 37 | 1550943 | 54.7+-21.1 | Bacteroides | 15/17 | 1.0 | .48 | 22 | 1/0/0 | 1372 |
| 22 | 74 | 3180913 | 1176.1+-342.7 | Pseudomonas | 14/28 | 1.0 | .45 | 42 | 4/0/0 | 3073 |
| 23 | 34 | 1185813 | 153.2+-7.5 | Clostridiales | 14/24 | 1.0 | .45 | 22 | 2/0/0 | 1239 |
| 24 | 17 | 77648 | 302.0+-962.3 | Prevotella | 15/15 | 1.1 | .45 | 7 | 1/1/1 | 78 |
| 25 | 65 | 322841 | 15.3+-2.8 | Xanthomonadaceae | 14/15 | 1.0 | .45 | 8 | 0/0/0 | 310 |
| 26 | 31 | 253262 | 61.3+-146.0 | Synergistaceae | 13/13 | 1.0 | .41 | 6 | 1/0/0 | 296 |
| 27 | 7 | 247319 | 675.5+-233.2 | Bacteroidales | 13/13 | 1.0 | .41 | 2 | 2/0/0 | 246 |
| 28 | 61 | 6068420 | 59.1+-12.6 | Bacteroides | 23/23 | 1.8 | .41 | 77 | 7/0/0 | 4814 |
| 29 | 8 | 119680 | 35.6+-38.2 | Enterobacteriaceae | 13/13 | 1.0 | .41 | 2 | 1/0/0 | 138 |
| 30 | 29 | 1052965 | 139.5+-33.6 | Ruminococcaceae | 12/19 | 1.0 | .38 | 22 | 0/0/0 | 1112 |
| 31 | 19 | 1541549 | 48.7+-4.8 | Coriobacterium | 12/13 | 1.0 | .38 | 43 | 3/0/0 | 1383 |
| 32 | 15 | 38337 | 10.5+-2.6 | Clostridium | 12/13 | 1.0 | .38 | 0 | 0/0/0 | 40 |
| 33 | 206 | 1951851 | 19.9+-5.8 | Clostridiales | 16/16 | 1.5 | .35 | 12 | 0/0/0 | 2095 |
| 34 | 81 | 1276098 | 12.1+-1.1 | Synergistaceae | 11/12 | 1.0 | .35 | 25 | 0/0/0 | 1247 |
| 35 | 34 | 146116 | 24.2+-16.9 | Parabacteroides | 11/23 | 1.0 | .35 | 1 | 0/0/0 | 186 |
| 36 | 11 | 68681 | 558.7+-589.5 | Acinetobacter | 14/25 | 1.3 | .35 | 2 | 0/0/0 | 93 |
| 37 | 120 | 1298872 | 13.2+-1.3 | Veillonella | 10/ 22 | 1.0 | .32 | 40 | 1/0/0 | 1247 |
| 38 | 36 | 1433264 | 15.9+-1.2 | Ruminococcaceae | 10/14 | 1.0 | .32 | 22 | 1/0/0 | 1499 |
| 39 | 72 | 3218616 | 21.7+-5.2 | Bacteroides | 10/10 | 1.0 | .32 | 24 | 3/0/0 | 2527 |
| 40 | 13 | 857212 | 723.3+-95.5 | Bacteroidales | 10/10 | 1.0 | .32 | 14 | 0/0/0 | 726 |
| 41 | 245 | 2264103 | 16.0+-2.4 | Pseudomonas | 12/13 | 1.3 | .29 | 27 | 3/0/0 | 2269 |
| 42 | 25 | 418684 | 62.7+-63.3 | Ruminococcaceae | 9/15 | 1.0 | .29 | 6 | 0/0/0 | 508 |
| 43 | 165 | 1589260 | 10.8+-0.9 | Prevotella | 8/8 | 1.0 | .25 | 10 | 1/0/0 | 1328 |
| 44 | 34 | 1108833 | 46.7+-24.6 | Bacteroides | 8/9 | 1.0 | .25 | 16 | 0/0/0 | 1029 |
| 45 | 77 | 3326270 | 88.9+-9.5 | Enterobacteriaceae | 8/8 | 1.0 | .25 | 54 | 5/0/0 | 3177 |
| 46 | 71 | 200120 | 8.7+-4.4 | Clostridiales | 9/10 | 1.1 | .25 | 4 | 0/0/0 | 250 |
| 47 | 167 | 666453 | 172.7+-330.7 | Psychrobacter | 9/9 | 1.1 | .25 | 3 | 1/0/0 | 683 |
| 48 | 56 | 376754 | 363.2+-569.6 | Psychrobacter | 12/25 | 1.5 | .25 | 0 | 0/0/0 | 499 |
| 49 | 35 | 2041463 | 723.6+-78.2 | Bacteroidales | 7/8 | 1.0 | .22 | 31 | 2/0/0 | 1617 |
| 50 | 93 | 496387 | 107.7+-69.1 | Acinetobacter | 7/7 | 1.0 | .22 | 24 | 0/0/0 | 557 |
| 51 | 45 | 1254043 | 22.9+-10.3 | Parabacteroides | 5/9 | 1.0 | .16 | 11 | 0/0/0 | 1024 |
| 52 | 96 | 383960 | 228.5+-303.9 | Psychrobacter | 6/6 | 1.2 | .16 | 4 | 1/0/0 | 426 |
| 53 | 44 | 674481 | 78.8+-31.0 | Enterobacteriaceae | 4/4 | 1.0 | .12 | 12 | 1/0/0 | 686 |
| 54 | 37 | 850792 | 86.2+-19.6 | Enterobacteriaceae | 4/4 | 1.0 | .12 | 13 | 0/0/0 | 831 |
| 55 | 26 | 1394535 | 28.9+-2.6 | Ruminococcus | 4/10 | 1.0 | .12 | 10 | 0/0/0 | 1345 |
| 56 | 188 | 1251229 | 65.7+-41.1 | Acinetobacter | 3/3 | 1.0 | .09 | 16 | 1/0/0 | 1445 |
| 57 | 29 | 712468 | 44.5+-72.8 | Clostridiales | 3/3 | 1.0 | .09 | 4 | 1/0/0 | 773 |
| 58 | 76 | 1362188 | 57.3+-90.8 | Bacteroides | 5/5 | 1.7 | .09 | 33 | 1/0/0 | 1357 |
| 59 | 51 | 502581 | 27.4+-24.1 | Clostridiales | 3/3 | 1.0 | .09 | 7 | 0/0/0 | 594 |
| 60 | 30 | 1394073 | 41.3+-43.8 | Parabacteroides | 3/5 | 1.0 | .09 | 21 | 0/1/1 | 1178 |
| 61 | 19 | 58861 | 16.6+-14.1 | Clostridium | 3/4 | 1.0 | .09 | 1 | 0/0/0 | 74 |
| 62 | 58 | 332317 | 34.5+-78.5 | Bacteroides | 3/4 | 1.0 | .09 | 2 | 0/0/0 | 376 |
| 63 | 23 | 323314 | 49.9+-69.0 | Bacteroides | 3/3 | 1.0 | .09 | 9 | 1/0/0 | 321 |
| 64 | 45 | 399721 | 38.3+-41.9 | Clostridiales | 2/3 | 1.0 | .06 | 10 | 2/0/0 | 508 |
| 65 | 2 | 43231 | 13.9+-0.1 | Clostridiales | 2/2 | 1.0 | .06 | 0 | 0/0/0 | 62 |
| 66 | 30 | 356678 | 57.8+-57.1 | Enterobacteriaceae | 2/2 | 1.0 | .06 | 13 | 2/0/0 | 479 |
| 67 | 5 | 163222 | 31.3+-1.5 | Veillonella | 2/2 | 1.0 | .06 | 7 | 2/0/0 | 155 |
| 68 | 55 | 603823 | 68.5+-109.6 | Prevotella | 3/3 | 1.5 | .06 | 7 | 0/0/0 | 674 |
| 69 | 18 | 459715 | 30.1+-1.4 | Veillonella | 2/3 | 1.0 | .06 | 8 | 2/0/0 | 458 |
| 70 | 15 | 706010 | 831.6+-110.7 | Bacteroidales | 2/3 | 1.0 | .06 | 8 | 1/0/0 | 595 |
| 71 | 19 | 52440 | 9.9+-1.8 | Prevotella | 2/2 | 1.0 | .06 | 1 | 0/0/0 | 57 |
| 72 | 24 | 74332 | 11.8+-4.2 | Pseudomonas | 2/2 | 1.0 | .06 | 0 | 0/0/0 | 84 |
| 73 | 94 | 286851 | 10.0+-2.7 | Clostridium | 3/3 | 1.5 | .06 | 0 | 0/0/0 | 307 |
| 74 | 46 | 208897 | 27.7+-27.8 | Sphingobacterium | 3/4 | 1.5 | .06 | 1 | 0/0/0 | 234 |
| 75 | 47 | 284969 | 10.8+-0.8 | Prevotella | 2/2 | 1.0 | .06 | 4 | 0/0/0 | 250 |
| 76 | 87 | 558714 | 18.6+-6.8 | Clostridiales | 2/4 | 1.0 | .06 | 6 | 0/0/0 | 656 |
| 77 | 12 | 98345 | 115.3+-105.5 | Veillonella | 2/2 | 1.0 | .06 | 0 | 0/0/0 | 103 |
| 78 | 66 | 399221 | 17.4+-15.8 | Pseudomonas | 2/2 | 1.0 | .06 | 9 | 0/0/0 | 430 |
| 79 | 101 | 701349 | 39.7+-36.6 | Acinetobacter | 1/1 | 1.0 | .03 | 6 | 0/0/0 | 752 |
| 80 | 253 | 2109472 | 14.0+-2.2 | Pseudomonas | 1/1 | 1.0 | .03 | 7 | 2/0/0 | 2127 |
| 81 | 10 | 174036 | 214.5+-356.6 | Ruminococcaceae | 1/1 | 1.0 | .03 | 4 | 1/1/1 | 219 |
| 82 | 30 | 559132 | 44.7+-52.9 | Ethanoligenens | 1/1 | 1.0 | .03 | 6 | 1/0/0 | 685 |
| 83 | 4 | 277347 | 35.3+-1.6 | Atopobium | 1/1 | 1.0 | .03 | 20 | 0/0/0 | 245 |
| 84 | 19 | 42313 | 13.3+-3.6 | Stenotrophomonas | 1/1 | 1.0 | .03 | 0 | 0/0/0 | 57 |
| 85 | 14 | 33591 | 19.5+-14.9 | Bacteroides | 1/1 | 1.0 | .03 | 1 | 0/0/0 | 17 |
| 86 | 35 | 86834 | 13.8+-10.2 | Sphingobacteriaceae | 1/1 | 1.0 | .03 | 5 | 0/0/0 | 108 |
| 87 | 22 | 57181 | 33.1+-47.4 | Psychrobacter | 1/1 | 1.0 | .03 | 0 | 0/0/0 | 82 |
| 88 | 25 | 68405 | 33.6+-66.4 | Psychrobacter | 2/3 | 2.0 | .03 | 0 | 0/0/0 | 65 |
| 89 | 38 | 119481 | 16.5+-4.9 | Pseudomonas | 1/1 | 1.0 | .03 | 0 | 0/0/0 | 137 |
| 90 | 25 | 86639 | 32.5+-30.1 | Acinetobacter | 1/1 | 1.0 | .03 | 3 | 0/0/0 | 109 |
| 91 | 63 | 195403 | 194.1+-327.0 | Psychrobacter | 2/2 | 2.0 | .03 | 2 | 0/0/0 | 172 |
| 92 | 77 | 242185 | 11.7+-11.0 | Clostridium | 1/1 | 1.0 | .03 | 2 | 0/0/0 | 319 |
| 93 | 38 | 138619 | 57.6+-114.5 | Clostridium | 1/1 | 1.0 | .03 | 2 | 0/0/0 | 201 |
| 94 | 77 | 298810 | 15.9+-7.5 | Pseudomonas | 1/1 | 1.0 | .03 | 1 | 0/0/0 | 326 |
| 95 | 74 | 266915 | 42.3+-110.1 | Clostridiales | 1/1 | 1.0 | .03 | 0 | 4/0/0 | 280 |
| 96 | 25 | 95007 | 16.7+-5.0 | Pseudomonas | 1/1 | 1.0 | .03 | 1 | 0/0/0 | 103 |
| 97 | 73 | 318825 | 50.7+-43.5 | Acinetobacter | 1/1 | 1.0 | .03 | 0 | 0/0/0 | 394 |
| 98 | 30 | 107088 | 15.2+-14.0 | Ruminococcaceae | 1/1 | 1.0 | .03 | 0 | 1/1/1 | 114 |
| 99 | 50 | 174781 | 18.5+-39.1 | Parabacteroides | 1/1 | 1.0 | .03 | 2 | 0/1/1 | 177 |
| 100 | 107 | 470496 | 12.6+-1.5 | Selenomonadales | 1/1 | 1.0 | .03 | 9 | 0/0/0 | 441 |
| 101 | 178 | 994562 | 150.8+-290.5 | Psychrobacter | 1/1 | 1.0 | .03 | 4 | 1/0/0 | 1026 |
| 102 | 154 | 708764 | 69.7+-44.1 | Acinetobacter | 1/1 | 1.0 | .03 | 3 | 0/0/0 | 783 |
| 103 | 59 | 399560 | 10.6+-1.2 | Prevotella | 1/1 | 1.0 | .03 | 6 | 0/0/0 | 351 |
| 104 | 20 | 214590 | 16.7+-1.5 | - | 0/0 | 0 | - | 1 | 2/0/0 | 237 |
| 105 | 14 | 238172 | 99.7+-61.6 | - | 0/0 | 0 | - | 3 | 0/0/0 | 278 |
| 106 | 24 | 262537 | 44.2+-50.7 | - | 0/0 | 0 | - | 3 | 0/0/0 | 293 |
| 107 | 20 | 1435728 | 27.7+-2.9 | - | 0/0 | 0 | - | 22 | 0/0/0 | 1421 |
| 108 | 11 | 209463 | 63.3+-51.2 | - | 0/0 | 0 | - | 3 | 0/0/0 | 244 |
| 109 | 154 | 1071701 | 71.1+-123.4 | - | 0/0 | 0 | - | 7 | 0/0/0 | 1041 |
| 110 | 1 | 36755 | 27.0+-0.0 | - | 0/0 | 0 | - | 0 | 0/0/0 | 61 |
| 111 | 13 | 204636 | 26.6+-2.1 | - | 0/0 | 0 | - | 6 | 0/0/0 | 211 |
| 112 | 13 | 184203 | 122.1+-69.2 | - | 0/0 | 0 | - | 0 | 0/0/0 | 227 |
| 113 | 1 | 39296 | 20.0+-0.0 | - | 0/0 | 0 | - | 0 | 0/0/0 | 42 |
| 114 | 16 | 333087 | 159.1+-5.6 | - | 0/0 | 0 | - | 2 | 0/0/0 | 326 |
| 115 | 16 | 252812 | 30.3+-20.6 | - | 0/0 | 0 | - | 0 | 1/0/0 | 295 |
| 116 | 1 | 42699 | 23.0+-0.0 | - | 0/0 | 0 | - | 0 | 0/0/0 | 60 |
| 117 | 8 | 146979 | 63.1+-36.8 | - | 0/0 | 0 | - | 2 | 0/0/0 | 191 |
| 118 | 1 | 44027 | 42.0+-0.0 | - | 0/0 | 0 | - | 0 | 0/0/0 | 55 |
| 119 | 5 | 63062 | 21262.3+-14457.8 | - | 0/0 | 0 | - | 2 | 0/0/0 | 81 |
| 120 | 70 | 755943 | 88.6+-147.0 | - | 0/0 | 0 | - | 9 | 0/0/0 | 954 |
| 121 | 1 | 49335 | 68.0+-0.0 | - | 0/0 | 0 | - | 0 | 0/0/0 | 79 |
| 122 | 3 | 108961 | 1573.4+-92.8 | - | 0/0 | 0 | - | 12 | 0/0/0 | 157 |
| 123 | 38 | 481548 | 94.5+-79.7 | - | 0/0 | 0 | - | 3 | 1/0/0 | 607 |
| 124 | 2 | 101811 | 242.1+-36.7 | - | 0/0 | 0 | - | 0 | 0/0/0 | 126 |
| 125 | 34 | 434108 | 58.6+-23.8 | - | 0/0 | 0 | - | 6 | 0/0/0 | 470 |
| 126 | 22 | 218712 | 75.7+-58.9 | - | 0/0 | 0 | - | 1 | 0/0/0 | 269 |
| 127 | 45 | 838468 | 50.2+-28.9 | - | 0/0 | 0 | - | 24 | 2/1/1 | 903 |
| 128 | 41 | 659181 | 187.2+-60.6 | - | 0/0 | 0 | - | 27 | 0/0/0 | 768 |
| 129 | 1 | 70508 | 35.0+-0.0 | - | 0/0 | 0 | - | 0 | 0/0/0 | 84 |
| 130 | 12 | 391462 | 180.9+-36.8 | - | 0/0 | 0 | - | 29 | 0/0/0 | 485 |
| 131 | 1 | 74662 | 17.0+-0.0 | - | 0/0 | 0 | - | 0 | 0/0/0 | 111 |
| 132 | 17 | 524249 | 15.8+-0.9 | - | 0/0 | 0 | - | 8 | 0/0/0 | 533 |
| 133 | 90 | 883839 | 22.1+-7.6 | - | 0/0 | 0 | - | 9 | 0/0/0 | 891 |
| 134 | 78 | 1190910 | 77.5+-59.4 | - | 0/0 | 0 | - | 7 | 0/0/0 | 1370 |
| 135 | 13 | 324277 | 63.6+-29.9 | - | 0/0 | 0 | - | 3 | 0/0/0 | 416 |
| 136 | 1 | 102210 | 409.0+-0.0 | - | 0/0 | 0 | - | 0 | 0/0/0 | 140 |
| 137 | 33 | 212391 | 17.1+-8.2 | - | 0/0 | 0 | - | 3 | 0/0/0 | 233 |
| 138 | 8 | 190171 | 21.4+-5.8 | - | 0/0 | 0 | - | 3 | 0/0/0 | 223 |
| 139 | 27 | 925058 | 71.8+-54.5 | - | 0/0 | 0 | - | 4 | 1/0/0 | 890 |
| 140 | 20 | 483292 | 94.8+-23.9 | - | 0/0 | 0 | - | 8 | 0/0/0 | 467 |
| 141 | 2 | 152888 | 31.0+-10.0 | - | 0/0 | 0 | - | 10 | 0/0/0 | 197 |
| 142 | 2 | 4002 | 9.5+-2.1 | - | 0/0 | 0 | - | 0 | 0/0/0 | 4 |
| 143 | 6 | 12077 | 8.5+-2.2 | - | 0/0 | 0 | - | 0 | 0/0/0 | 16 |
| 144 | 2 | 154001 | 287.5+-55.5 | - | 0/0 | 0 | - | 3 | 0/0/0 | 192 |
| 145 | 3 | 6072 | 8.0+-1.0 | - | 0/0 | 0 | - | 0 | 0/0/0 | 4 |
| 146 | 4 | 8072 | 117.3+-213.9 | - | 0/0 | 0 | - | 0 | 0/0/0 | 4 |
| 147 | 2 | 4073 | 175.4+-155.6 | - | 0/0 | 0 | - | 0 | 0/0/0 | 2 |
| 148 | 8 | 16357 | 10.9+-4.9 | - | 0/0 | 0 | - | 1 | 0/0/0 | 25 |
| 149 | 15 | 30737 | 9.2+-1.6 | - | 0/0 | 0 | - | 0 | 0/0/0 | 37 |
| 150 | 6 | 12554 | 19.0+-28.7 | - | 0/0 | 0 | - | 0 | 0/0/0 | 14 |
| 151 | 7 | 14693 | 9.1+-2.0 | - | 0/0 | 0 | - | 0 | 0/0/0 | 17 |
| 152 | 16 | 33602 | 10.3+-3.2 | - | 0/0 | 0 | - | 0 | 0/0/0 | 40 |
| 153 | 20 | 42376 | 13.3+-3.7 | - | 0/0 | 0 | - | 0 | 0/0/0 | 51 |
| 154 | 13 | 28017 | 62.7+-184.3 | - | 0/0 | 0 | - | 0 | 0/0/0 | 29 |
| 155 | 18 | 38495 | 8.5+-2.0 | - | 0/0 | 0 | - | 0 | 0/0/0 | 52 |
| 156 | 5 | 11151 | 7.2+-1.1 | - | 0/0 | 0 | - | 0 | 0/0/0 | 16 |
| 157 | 14 | 30983 | 101.7+-300.9 | - | 0/0 | 0 | - | 0 | 0/0/0 | 28 |
| 158 | 26 | 58226 | 9.7+-1.6 | - | 0/0 | 0 | - | 0 | 0/0/0 | 65 |
| 159 | 16 | 35876 | 10.2+-3.2 | - | 0/0 | 0 | - | 0 | 0/0/0 | 42 |
| 160 | 14 | 31285 | 179.5+-411.4 | - | 0/0 | 0 | - | 0 | 0/0/0 | 33 |
| 161 | 8 | 18411 | 106.3+-286.6 | - | 0/0 | 0 | - | 0 | 0/0/0 | 15 |
| 162 | 15 | 33591 | 14.9+-3.5 | - | 0/0 | 0 | - | 0 | 0/0/0 | 36 |
| 163 | 15 | 34451 | 12.1+-13.7 | - | 0/0 | 0 | - | 1 | 1/0/0 | 37 |
| 164 | 11 | 25379 | 42.6+-58.7 | - | 0/0 | 0 | - | 0 | 0/0/0 | 26 |
| 165 | 16 | 36624 | 9.1+-3.1 | - | 0/0 | 0 | - | 0 | 0/0/0 | 42 |
| 166 | 9 | 21627 | 15.9+-14.3 | - | 0/0 | 0 | - | 0 | 0/0/0 | 20 |
| 167 | 21 | 49923 | 55.0+-92.7 | - | 0/0 | 0 | - | 1 | 0/0/0 | 22 |
| 168 | 7 | 17941 | 9.4+-1.8 | - | 0/0 | 0 | - | 0 | 0/0/0 | 15 |
| 169 | 17 | 40089 | 31.9+-30.6 | - | 0/0 | 0 | - | 0 | 0/0/0 | 18 |
| 170 | 9 | 21625 | 8.8+-3.1 | - | 0/0 | 0 | - | 0 | 0/0/0 | 26 |
| 171 | 21 | 51606 | 12.9+-3.0 | - | 0/0 | 0 | - | 0 | 0/0/0 | 51 |
| 172 | 32 | 80716 | 9.4+-11.8 | - | 0/0 | 0 | - | 1 | 0/0/0 | 92 |
| 173 | 13 | 33475 | 10.7+-4.3 | - | 0/0 | 0 | - | 5 | 1/0/0 | 48 |
| 174 | 12 | 30167 | 482.2+-1337.3 | - | 0/0 | 0 | - | 9 | 0/2/3 | 31 |
| 175 | 3 | 8775 | 41.5+-34.3 | - | 0/0 | 0 | - | 0 | 0/0/0 | 8 |
| 176 | 27 | 67967 | 14.1+-27.1 | - | 0/0 | 0 | - | 0 | 0/0/0 | 64 |
| 177 | 9 | 25693 | 313.7+-811.9 | - | 0/0 | 0 | - | 0 | 0/0/1 | 27 |
| 178 | 42 | 111360 | 12.8+-27.3 | - | 0/0 | 0 | - | 0 | 0/0/0 | 149 |
| 179 | 50 | 138420 | 7.1+-1.1 | - | 0/0 | 0 | - | 1 | 0/0/0 | 134 |
| 180 | 28 | 77931 | 17.2+-23.5 | - | 0/0 | 0 | - | 0 | 0/0/0 | 96 |
| 181 | 23 | 71503 | 13.4+-11.9 | - | 0/0 | 0 | - | 1 | 0/0/0 | 72 |
| 182 | 53 | 151349 | 81.1+-178.7 | - | 0/0 | 0 | - | 3 | 0/0/0 | 149 |
| 183 | 52 | 143895 | 17.1+-9.3 | - | 0/0 | 0 | - | 0 | 0/0/0 | 154 |
| 184 | 15 | 47450 | 20.8+-17.9 | - | 0/0 | 0 | - | 0 | 0/0/0 | 36 |
| 185 | 26 | 71213 | 11.6+-10.6 | - | 0/0 | 0 | - | 0 | 0/0/0 | 76 |
| 186 | 46 | 124942 | 15.4+-30.0 | - | 0/0 | 0 | - | 0 | 0/0/1 | 122 |
| 187 | 33 | 109949 | 13.2+-2.9 | - | 0/0 | 0 | - | 0 | 0/0/0 | 108 |
| 188 | 51 | 162429 | 170.6+-295.0 | - | 0/0 | 0 | - | 0 | 0/0/0 | 180 |
| 189 | 15 | 50651 | 13.4+-6.1 | - | 0/0 | 0 | - | 0 | 0/0/0 | 39 |
| 190 | 54 | 190711 | 12.2+-2.7 | - | 0/0 | 0 | - | 1 | 0/0/0 | 209 |
| 191 | 14 | 48281 | 51.5+-92.5 | - | 0/0 | 0 | - | 1 | 0/0/0 | 48 |
| 192 | 26 | 92204 | 14.7+-3.3 | - | 0/0 | 0 | - | 0 | 0/0/0 | 94 |
| 193 | 42 | 144910 | 44.8+-67.3 | - | 0/0 | 0 | - | 3 | 0/0/0 | 135 |
| 194 | 14 | 46004 | 10.5+-2.2 | - | 0/0 | 0 | - | 1 | 0/0/0 | 53 |
| 195 | 8 | 35315 | 130.4+-109.3 | - | 0/0 | 0 | - | 1 | 0/0/0 | 49 |
| 196 | 16 | 58932 | 235.6+-398.5 | - | 0/0 | 0 | - | 0 | 0/0/0 | 97 |
| 197 | 31 | 120065 | 47.2+-67.1 | - | 0/0 | 0 | - | 0 | 0/0/0 | 130 |
| 198 | 23 | 101265 | 388.1+-1119.0 | - | 0/0 | 0 | - | 27 | 8/8/14 | 64 |
| 199 | 40 | 148900 | 13.0+-2.8 | - | 0/0 | 0 | - | 1 | 0/0/0 | 130 |
| 200 | 17 | 61322 | 13.9+-2.1 | - | 0/0 | 0 | - | 0 | 0/0/0 | 71 |
| 201 | 20 | 66854 | 14.9+-1.7 | - | 0/0 | 0 | - | 1 | 0/0/0 | 56 |
| 202 | 14 | 63366 | 43.2+-56.2 | - | 0/0 | 0 | - | 0 | 0/0/0 | 54 |
| 203 | 20 | 89126 | 43.0+-63.0 | - | 0/0 | 0 | - | 2 | 0/0/0 | 123 |
| 204 | 41 | 167797 | 112.4+-282.4 | - | 0/0 | 0 | - | 8 | 0/0/0 | 197 |
| 205 | 12 | 51897 | 125.1+-293.3 | - | 0/0 | 0 | - | 0 | 0/0/0 | 48 |
| 206 | 16 | 78815 | 28.7+-39.9 | - | 0/0 | 0 | - | 9 | 1/0/0 | 102 |
| 207 | 34 | 146368 | 30.2+-56.2 | - | 0/0 | 0 | - | 2 | 0/0/0 | 166 |
| 208 | 135 | 548142 | 14.0+-2.5 | - | 0/0 | 0 | - | 9 | 0/0/0 | 539 |
| 209 | 22 | 114895 | 190.7+-247.2 | - | 0/0 | 0 | - | 0 | 0/0/0 | 180 |
| 210 | 55 | 261695 | 22.1+-19.0 | - | 0/0 | 0 | - | 1 | 0/0/0 | 277 |
| 211 | 7 | 49910 | 86.3+-75.4 | - | 0/0 | 0 | - | 4 | 0/0/0 | 81 |
| 212 | 21 | 141225 | 37.5+-46.0 | - | 0/0 | 0 | - | 1 | 0/0/0 | 192 |
| 213 | 35 | 173222 | 100.2+-187.5 | - | 0/0 | 0 | - | 1 | 0/0/0 | 245 |
| 214 | 51 | 316264 | 54.4+-68.4 | - | 0/0 | 0 | - | 0 | 0/0/0 | 403 |
| 215 | 9 | 47408 | 17.1+-8.6 | - | 0/0 | 0 | - | 0 | 0/0/0 | 58 |
| 216 | 84 | 530519 | 11.0+-1.0 | - | 0/0 | 0 | - | 3 | 0/0/0 | 413 |
| 217 | 16 | 111174 | 275.5+-459.2 | - | 0/0 | 0 | - | 5 | 0/1/0 | 108 |
| 218 | 17 | 119018 | 51.3+-57.6 | - | 0/0 | 0 | - | 4 | 0/1/1 | 138 |
| 219 | 34 | 2312837 | 35.8+-4.3 | - | 0/0 | 0 | - | 44 | 1/1/1 | 2191 |
| 220 | 1 | 16647 | 107.0+-0.0 | - | 0/0 | 0 | - | 0 | 0/0/0 | 19 |
| 221 | 494 | 1907161 | 9.9+-1.4 | - | 0/0 | 0 | - | 6 | 1/1/1 | 2060 |
| 222 | 350 | 1706242 | 13.6+-2.2 | - | 0/0 | 0 | - | 18 | 0/0/0 | 1798 |
| 223 | 3 | 56655 | 23.7+-8.0 | - | 0/0 | 0 | - | 0 | 0/0/0 | 53 |
| 224 | 314 | 2301828 | 15.2+-2.1 | - | 0/0 | 0 | - | 6 | 1/0/0 | 2222 |
| 225 | 84 | 813042 | 12.0+-1.0 | - | 0/0 | 0 | - | 9 | 0/0/0 | 805 |
| 226 | 17 | 142885 | 36.6+-63.9 | - | 0/0 | 0 | - | 1 | 0/0/0 | 189 |
| 227 | 9 | 907982 | 61.1+-1.7 | - | 0/0 | 0 | - | 10 | 1/0/0 | 757 |
| 228 | 290 | 2070754 | 12.5+-1.7 | - | 0/0 | 0 | - | 5 | 0/0/0 | 2015 |
| 229 | 18 | 146254 | 15.8+-3.7 | - | 0/0 | 0 | - | 3 | 0/0/0 | 157 |
| 230 | 82 | 576370 | 39.9+-38.9 | - | 0/0 | 0 | - | 5 | 1/0/0 | 706 |
| 231 | 4 | 69030 | 30.0+-27.7 | - | 0/0 | 0 | - | 0 | 0/0/0 | 90 |
| 232 | 51 | 362246 | 60.6+-99.7 | - | 0/0 | 0 | - | 0 | 0/0/0 | 425 |
| 233 | 82 | 563089 | 207.6+-342.1 | - | 0/0 | 0 | - | 2 | 0/0/0 | 558 |
| 234 | 4 | 59023 | 27.5+-1.7 | - | 0/0 | 0 | - | 0 | 0/0/0 | 10 |
