# Supplementary material for: A Comparative Metagenome Survey of the Fecal Microbiota of a Breast- and a Plant-Fed Asian Elephant Reveals an Unexpectedly High Diversity of Glycoside Hydrolase Family Enzymes
Source: PLoS One. 2014 Sep 10;9(9):e106707. doi: 10.1371/journal.pone.0106707 (PMC4160196; doi:10.1371/journal.pone.0106707)
Supplement: Table S3 — Bins observed for the six-years-old elephant. (DOCX) [file pone.0106707.s004.docx]

Table S3: Bins observed for the six-years-old elephant.

| Bin id | Number of scaffolds | Nucleotides | Average coverage | Taxonomic assignment | Supporting/all markers | Average marker copy number | Estimated completeness | tRNAs | rRNAs 5s/rRNAs 16s/rRNAs 23s | Number of genes |  |
| --- | --- | --- | --- | --- | --- | --- | --- | --- | --- | --- | --- |
| 1 | 125 | 2307036 | 16.6+-2.4 | Bacteroidetes | 30/31 | 1.0 | .96 | 40 | 1/0/0 | 2039 |  |
| 2 | 93 | 2546626 | 117.4+-33.0 | Clostridiales | 30/30 | 1.1 | .90 | 28 | 0/0/0 | 2258 |  |
| 3 | 52 | 2096428 | 52.4+-7.2 | Ruminococcaceae | 27/30 | 1.0 | .87 | 28 | 2/0/0 | 1924 |  |
| 4 | 41 | 1723109 | 31.3+-4.0 | Clostridiales | 26/30 | 1.0 | .83 | 41 | 3/0/0 | 1702 |  |
| 5 | 89 | 1644988 | 133.6+-22.1 | Campylobacter | 26/30 | 1.0 | .80 | 40 | 0/0/0 | 1840 |  |
| 6 | 166 | 1232225 | 15.5+-9.0 | Clostridiales | 30/33 | 1.2 | .80 | 14 | 1/0/0 | 1253 |  |
| 7 | 167 | 2507114 | 18.6+-2.2 | Clostridiales | 25/25 | 1.0 | .77 | 17 | 0/0/0 | 2180 |  |
| 8 | 202 | 3825414 | 26.4+-13.7 | Erysipelothrix | 64/111 | 2.7 | .77 | 93 | 3/2/1 | 3662 |  |
| 9 | 72 | 2358312 | 25.0+-3.0 | Bacteroidales | 24/30 | 1.0 | .77 | 34 | 0/0/0 | 1875 |  |
| 10 | 118 | 2147470 | 122.3+-79.0 | Bacteroidales | 27/27 | 1.2 | .74 | 21 | 2/0/0 | 1773 |  |
| 11 | 78 | 1764926 | 21.7+-2.2 | Bacteroidales | 24/31 | 1.0 | .74 | 28 | 0/0/0 | 1466 |  |
| 12 | 51 | 1846008 | 76.7+-82.6 | Prevotella | 30/35 | 1.3 | .74 | 29 | 1/0/0 | 1460 |  |
| 13 | 78 | 2942798 | 140.2+-95.2 | Prevotella | 35/36 | 1.5 | .74 | 80 | 4/0/0 | 2419 |  |
| 14 | 144 | 986254 | 13.7+-9.3 | Clostridiales | 22/28 | 1.0 | .70 | 8 | 0/0/0 | 915 |  |
| 15 | 104 | 2114689 | 21.0+-7.0 | Clostridiales | 24/24 | 1.1 | .70 | 30 | 0/0/0 | 1966 |  |
| 16 | 40 | 2584886 | 20.4+-2.1 | Prevotella | 22/32 | 1.0 | .70 | 47 | 5/0/0 | 1925 |  |
| 17 | 20 | 380705 | 26.3+-21.0 | Bacteroidales | 22/22 | 1.0 | .70 | 9 | 0/0/0 | 417 |  |
| 18 | 104 | 1506625 | 38.1+-6.1 | Bacteroidales | 23/28 | 1.0 | .70 | 33 | 0/0/0 | 1332 |  |
| 19 | 98 | 2667892 | 19.2+-1.7 | Clostridiales | 21/31 | 1.0 | .67 | 49 | 3/0/0 | 2578 |  |
| 20 | 38 | 825512 | 54.7+-26.6 | Prevotella | 21/21 | 1.1 | .64 | 11 | 0/0/0 | 669 |  |
| 21 | 47 | 313220 | 63.1+-58.8 | Bacteroidales | 68/69 | 3.4 | .64 | 4 | 0/0/0 | 309 |  |
| 22 | 149 | 2711960 | 36.0+-25.5 | Clostridiales | 30/62 | 1.5 | .64 | 47 | 1/2/1 | 2444 |  |
| 23 | 100 | 1699294 | 88.6+-94.5 | Prevotella | 21/24 | 1.1 | .64 | 16 | 0/0/0 | 1338 |  |
| 24 | 28 | 2462096 | 27.2+-1.8 | Bacteroidales | 23/33 | 1.1 | .64 | 35 | 2/0/0 | 2012 |  |
| 25 | 26 | 1061352 | 67.1+-8.4 | Ruminococcaceae | 19/26 | 1.0 | .61 | 30 | 2/0/0 | 983 |  |
| 26 | 144 | 3246765 | 25.4+-11.1 | Bacteroidales | 32/50 | 1.7 | .61 | 46 | 0/0/0 | 2819 |  |
| 27 | 49 | 2614059 | 98.1+-12.7 | Bacteroidales | 19/31 | 1.0 | .61 | 36 | 2/0/0 | 2259 |  |
| 28 | 75 | 914092 | 14.5+-1.7 | Ruminococcaceae | 20/27 | 1.1 | .61 | 14 | 1/0/0 | 882 |  |
| 29 | 120 | 1194658 | 22.1+-26.8 | Erysipelothrix | 19/23 | 1.0 | .61 | 14 | 0/0/0 | 1162 |  |
| 30 | 118 | 2514420 | 28.0+-15.2 | Bacteroidales | 21/32 | 1.2 | .58 | 48 | 0/0/0 | 2320 |  |
| 31 | 376 | 4834788 | 34.5+-25.6 | Bacteroidales | 25/33 | 1.4 | .58 | 60 | 0/0/0 | 4142 |  |
| 32 | 197 | 1099417 | 12.1+-2.9 | Treponema | 20/21 | 1.1 | .58 | 19 | 0/0/0 | 1062 |  |
| 33 | 40 | 1391113 | 20.2+-1.7 | Ruminococcaceae | 19/32 | 1.1 | .58 | 34 | 1/0/0 | 1258 |  |
| 34 | 53 | 896568 | 14.4+-1.2 | Ruminococcaceae | 18/23 | 1.0 | .58 | 11 | 3/0/0 | 812 |  |
| 35 | 165 | 2085912 | 17.9+-5.7 | Bacteroidales | 19/20 | 1.1 | .58 | 33 | 0/0/0 | 1785 |  |
| 36 | 105 | 1552256 | 18.5+-2.4 | Bacteroidales | 18/23 | 1.1 | .54 | 35 | 1/0/0 | 1354 |  |
| 37 | 72 | 1462084 | 22.7+-3.1 | Clostridiales | 17/28 | 1.0 | .54 | 28 | 1/0/0 | 1694 |  |
| 38 | 64 | 614116 | 32.5+-39.8 | Bacteroidales | 21/21 | 1.2 | .54 | 11 | 0/0/0 | 618 |  |
| 39 | 130 | 2348191 | 15.9+-1.9 | Fibrobacter | 17/21 | 1.0 | .54 | 33 | 3/0/0 | 2076 |  |
| 40 | 191 | 1851299 | 25.3+-15.5 | Clostridiales | 26/40 | 1.5 | .54 | 30 | 1/0/0 | 1714 |  |
| 41 | 75 | 1101003 | 41.7+-35.0 | Clostridiales | 17/17 | 1.1 | .51 | 21 | 1/0/0 | 891 |  |
| 42 | 22 | 163445 | 141.3+-382.8 | Clostridiales | 67/74 | 4.2 | .51 | 0 | 0/0/0 | 218 |  |
| 43 | 17 | 310897 | 19.2+-9.4 | Clostridiales | 16/25 | 1.0 | .51 | 3 | 0/0/0 | 310 |  |
| 44 | 200 | 1637476 | 17.7+-7.8 | Mycoplasmataceae | 40/62 | 2.5 | .51 | 41 | 1/0/0 | 3426 |  |
| 45 | 263 | 2645269 | 62.3+-29.9 | Bacteroidales | 19/27 | 1.2 | .51 | 40 | 0/0/0 | 2259 |  |
| 46 | 35 | 183560 | 26.9+-30.4 | Bacteroidales | 19/20 | 1.3 | .48 | 2 | 1/0/0 | 180 |  |
| 47 | 14 | 151333 | 14.3+-2.9 | Clostridiales | 23/23 | 1.5 | .48 | 3 | 0/0/0 | 131 |  |
| 48 | 170 | 3003671 | 28.5+-5.1 | Bacteroidales | 25/25 | 1.7 | .48 | 64 | 0/0/0 | 2522 |  |
| 49 | 85 | 2647708 | 16.3+-1.3 | Clostridiales | 17/17 | 1.1 | .48 | 41 | 0/0/0 | 2316 |  |
| 50 | 59 | 572164 | 19.5+-19.6 | Bacteroidales | 27/32 | 1.8 | .48 | 16 | 0/0/0 | 556 |  |
| 51 | 120 | 2910232 | 19.0+-2.7 | Treponema | 18/20 | 1.2 | .48 | 55 | 2/0/0 | 2523 |  |
| 52 | 163 | 2120348 | 34.5+-17.0 | Euryarchaeota | 83/119 | 1.7 | .46 | 63 | 4/0/0 | 2128 |  |
| 53 | 51 | 260590 | 21.5+-22.2 | Treponema | 33/109 | 2.4 | .45 | 4 | 0/0/0 | 359 |  |
| 54 | 74 | 544560 | 30.8+-10.3 | Bacteroidales | 19/21 | 1.4 | .45 | 10 | 0/0/0 | 604 |  |
| 55 | 104 | 1500304 | 46.3+-13.7 | Bacteroidales | 16/17 | 1.1 | .45 | 16 | 2/0/0 | 1216 |  |
| 56 | 123 | 1834152 | 71.2+-22.2 | Prevotella | 14/21 | 1.0 | .45 | 27 | 3/0/0 | 1439 |  |
| 57 | 92 | 1321250 | 18.7+-4.7 | Bacteroidales | 16/23 | 1.2 | .41 | 27 | 2/0/0 | 1163 |  |
| 58 | 18 | 1434692 | 30.4+-3.3 | Ruminococcaceae | 13/31 | 1.0 | .41 | 43 | 2/0/0 | 1422 |  |
| 59 | 43 | 231887 | 69.7+-285.3 | Bacteroidales | 25/31 | 1.9 | .41 | 4 | 0/0/0 | 271 |  |
| 60 | 30 | 298714 | 40.1+-50.3 | Butyrivibrio | 19/71 | 1.5 | .41 | 2 | 0/0/0 | 360 |  |
| 61 | 44 | 592284 | 14.4+-1.3 | Ruminococcaceae | 13/21 | 1.0 | .41 | 10 | 0/0/0 | 548 |  |
| 62 | 67 | 1603440 | 36.1+-3.4 | Paludibacter | 13/30 | 1.0 | .41 | 36 | 1/0/0 | 1382 |  |
| 63 | 65 | 1893967 | 21.0+-11.3 | Erysipelothrix | 18/33 | 1.4 | .41 | 15 | 0/0/0 | 1738 |  |
| 64 | 183 | 4253460 | 34.3+-34.4 | Erysipelothrix | 26/46 | 2.2 | .38 | 72 | 1/0/0 | 3945 |  |
| 65 | 104 | 1412641 | 13.8+-1.7 | Ruminococcus | 12/22 | 1.0 | .38 | 20 | 0/0/0 | 1371 |  |
| 66 | 153 | 2064687 | 29.1+-11.4 | Erysipelothrix | 20/49 | 1.7 | .38 | 51 | 1/0/1 | 1964 |  |
| 67 | 41 | 1002413 | 120.1+-92.9 | Erysipelothrix | 15/29 | 1.2 | .38 | 41 | 3/2/1 | 986 |  |
| 68 | 33 | 1190324 | 117.1+-20.8 | Paludibacter | 12/24 | 1.0 | .38 | 30 | 1/0/0 | 992 |  |
| 69 | 48 | 1541592 | 39.0+-3.7 | Ruminococcus | 12/31 | 1.0 | .38 | 26 | 1/0/0 | 1517 |  |
| 70 | 71 | 1611383 | 15.9+-2.0 | Erysipelothrix | 13/15 | 1.1 | .38 | 31 | 1/0/0 | 1601 |  |
| 71 | 47 | 1759024 | 38.5+-3.1 | Verrucomicrobia | 12/28 | 1.0 | .38 | 33 | 1/0/0 | 1585 |  |
| 72 | 40 | 1380139 | 27.4+-12.1 | Ruminococcus | 14/32 | 1.2 | .38 | 26 | 1/0/0 | 1348 |  |
| 73 | 224 | 2588082 | 83.7+-28.5 | Fibrobacter | 14/15 | 1.2 | .38 | 44 | 2/0/0 | 2345 |  |
| 74 | 152 | 2039758 | 15.9+-2.7 | Clostridiales | 14/14 | 1.2 | .38 | 18 | 0/0/0 | 1812 |  |
| 75 | 89 | 1386128 | 154.4+-25.3 | Bacteroidales | 11/25 | 1.0 | .35 | 36 | 2/0/0 | 1271 |  |
| 76 | 166 | 1130542 | 12.2+-1.6 | Clostridiales | 11/12 | 1.0 | .35 | 17 | 0/0/0 | 1078 |  |
| 77 | 290 | 1965846 | 12.3+-1.7 | Treponema | 12/19 | 1.1 | .35 | 14 | 0/0/0 | 1838 |  |
| 78 | 71 | 2134763 | 57.4+-10.6 | Verrucomicrobia | 13/16 | 1.2 | .35 | 30 | 2/0/0 | 1701 |  |
| 79 | 105 | 2168022 | 21.6+-4.3 | Clostridiales | 14/14 | 1.3 | .35 | 41 | 0/0/0 | 1894 |  |
| 80 | 95 | 1569908 | 18.3+-1.9 | Clostridiales | 14/15 | 1.3 | .35 | 19 | 0/0/0 | 1431 |  |
| 81 | 57 | 2667153 | 37.4+-6.9 | Prevotella | 11/19 | 1.0 | .35 | 38 | 3/0/0 | 2036 |  |
| 82 | 30 | 149887 | 27.1+-30.4 | Fibrobacter | 20/24 | 2.0 | .32 | 2 | 0/0/0 | 179 |  |
| 83 | 52 | 255988 | 35.3+-30.4 | Prevotella | 16/82 | 1.6 | .32 | 5 | 1/0/0 | 309 |  |
| 84 | 81 | 2221771 | 55.7+-6.1 | Verrucomicrobia | 14/33 | 1.4 | .32 | 34 | 1/0/0 | 1739 |  |
| 85 | 204 | 4415018 | 53.1+-26.4 | Bacteroidales | 16/20 | 1.6 | .32 | 93 | 2/0/0 | 3690 |  |
| 86 | 28 | 254994 | 23.1+-14.4 | Clostridiales | 19/48 | 1.9 | .32 | 2 | 0/0/0 | 313 |  |
| 87 | 22 | 495684 | 28.9+-7.5 | Ruminococcaceae | 11/20 | 1.1 | .32 | 12 | 0/0/0 | 489 |  |
| 88 | 179 | 1115902 | 13.9+-16.2 | Bacteroidales | 12/16 | 1.2 | .32 | 7 | 0/0/0 | 1075 |  |
| 89 | 328 | 2822667 | 26.5+-21.0 | Erysipelothrix | 17/40 | 1.7 | .32 | 91 | 3/0/0 | 2752 |  |
| 90 | 128 | 1771174 | 23.1+-8.0 | Erysipelothrix | 11/14 | 1.1 | .32 | 37 | 0/0/0 | 1679 |  |
| 91 | 57 | 1785197 | 39.1+-13.7 | Acholeplasmataceae | 10/ 30 | 1.0 | .32 | 54 | 4/0/2 | 1526 |  |
| 92 | 132 | 1127590 | 21.2+-13.1 | Euryarchaeota | 47/53 | 1.5 | .30 | 22 | 2/0/0 | 1105 |  |
| 93 | 65 | 1538885 | 70.3+-6.6 | Verrucomicrobia | 9/21 | 1.0 | .29 | 26 | 0/0/0 | 1230 |  |
| 94 | 29 | 95546 | 15.8+-12.2 | Clostridiales | 11/17 | 1.2 | .29 | 4 | 0/0/0 | 127 |  |
| 95 | 40 | 159309 | 14.1+-5.5 | Bacteroidales | 10/20 | 1.1 | .29 | 3 | 1/0/0 | 198 |  |
| 96 | 46 | 2562196 | 55.2+-11.4 | Prevotella | 9/12 | 1.0 | .29 | 43 | 4/0/0 | 2041 |  |
| 97 | 48 | 269123 | 40.8+-42.0 | Bacteroidales | 11/11 | 1.2 | .29 | 5 | 1/0/0 | 279 |  |
| 98 | 100 | 1656473 | 21.6+-3.8 | Erysipelothrix | 9/21 | 1.0 | .29 | 38 | 1/1/0 | 1498 |  |
| 99 | 51 | 344190 | 72.0+-25.8 | Butyrivibrio | 11/29 | 1.2 | .29 | 2 | 0/0/0 | 328 |  |
| 100 | 105 | 818282 | 24.2+-34.8 | Bacteroidales | 10/15 | 1.1 | .29 | 15 | 1/0/0 | 680 |  |
| 101 | 101 | 770230 | 13.9+-4.9 | Clostridiales | 11/12 | 1.2 | .29 | 6 | 0/0/0 | 700 |  |
| 102 | 138 | 2241325 | 21.3+-15.1 | Bacteroidales | 10/14 | 1.1 | .29 | 38 | 1/0/0 | 1981 |  |
| 103 | 110 | 1215888 | 74.4+-17.6 | Clostridiales | 9/9 | 1.0 | .29 | 7 | 0/0/0 | 1149 |  |
| 104 | 23 | 370884 | 28.7+-36.8 | Bacteroidales | 10/16 | 1.1 | .29 | 5 | 0/0/0 | 278 |  |
| 105 | 171 | 1764627 | 17.6+-4.1 | Bacteroidetes | 16/16 | 1.8 | .29 | 24 | 2/0/0 | 1593 |  |
| 106 | 243 | 3319962 | 66.1+-39.6 | Prevotella | 13/15 | 1.4 | .29 | 41 | 4/0/0 | 2731 |  |
| 107 | 118 | 1846157 | 16.5+-7.8 | Ruminococcus | 9/21 | 1.0 | .29 | 28 | 1/0/0 | 1630 |  |
| 108 | 67 | 567641 | 14.9+-6.9 | Clostridiales | 10/13 | 1.2 | .25 | 20 | 0/0/0 | 531 |  |
| 109 | 161 | 2074635 | 21.5+-19.3 | Bacteroidales | 9/9 | 1.1 | .25 | 13 | 3/0/0 | 1745 |  |
| 110 | 116 | 522748 | 22.4+-20.1 | Acidaminococcus | 11/17 | 1.4 | .25 | 11 | 0/0/0 | 579 |  |
| 111 | 77 | 570865 | 17.8+-10.3 | Bacteroidales | 8/8 | 1.0 | .25 | 4 | 1/0/0 | 504 |  |
| 112 | 120 | 815108 | 13.7+-4.8 | Ruminococcaceae | 8/11 | 1.0 | .25 | 5 | 0/0/0 | 847 |  |
| 113 | 147 | 1175077 | 52.2+-29.3 | Acidaminococcus | 9/16 | 1.1 | .25 | 38 | 0/0/0 | 1155 |  |
| 114 | 210 | 1460615 | 14.6+-10.7 | Treponema | 11/14 | 1.4 | .25 | 0 | 0/0/0 | 1298 |  |
| 115 | 62 | 625847 | 19.6+-13.7 | Bacteroidales | 12/12 | 1.5 | .25 | 2 | 1/0/0 | 387 |  |
| 116 | 153 | 1258355 | 17.9+-3.5 | Bacteroidetes | 8/9 | 1.0 | .25 | 17 | 0/0/0 | 1110 |  |
| 117 | 109 | 1609686 | 19.3+-22.5 | Bacteroidales | 8/10 | 1.0 | .25 | 24 | 0/0/0 | 1402 |  |
| 118 | 17 | 55994 | 38.7+-62.0 | Bacteroidales | 8/14 | 1.0 | .25 | 0 | 0/0/0 | 72 |  |
| 119 | 282 | 3066099 | 53.7+-40.0 | Paludibacter | 16/33 | 2.3 | .22 | 55 | 0/0/0 | 2922 |  |
| 120 | 31 | 156705 | 20.0+-16.4 | Ruminococcaceae | 8/13 | 1.1 | .22 | 2 | 0/0/0 | 168 |  |
| 121 | 78 | 456942 | 16.9+-15.9 | Erysipelothrix | 8/20 | 1.1 | .22 | 10 | 1/0/2 | 473 |  |
| 122 | 131 | 874873 | 15.5+-7.7 | Bacteroidales | 7/10 | 1.0 | .22 | 12 | 0/0/0 | 873 |  |
| 123 | 78 | 713964 | 19.9+-29.8 | Bacteroidales | 7/9 | 1.0 | .22 | 30 | 3/0/0 | 710 |  |
| 124 | 133 | 864910 | 54.5+-50.0 | Acidaminococcus | 7/14 | 1.0 | .22 | 6 | 0/0/0 | 922 |  |
| 125 | 191 | 1403515 | 20.4+-24.8 | Bacteroidales | 8/10 | 1.1 | .22 | 23 | 1/0/0 | 1363 |  |
| 126 | 53 | 570756 | 62.2+-27.8 | Prevotella | 7/11 | 1.0 | .22 | 19 | 0/0/0 | 457 |  |
| 127 | 29 | 277920 | 17.9+-11.5 | Ruminococcaceae | 7/8 | 1.0 | .22 | 13 | 1/0/0 | 318 |  |
| 128 | 141 | 1142324 | 14.9+-5.0 | Clostridiales | 7/11 | 1.0 | .22 | 14 | 0/0/0 | 1094 |  |
| 129 | 137 | 1442464 | 14.5+-2.1 | Ruminococcaceae | 7/11 | 1.0 | .22 | 19 | 1/0/0 | 1382 |  |
| 130 | 139 | 913518 | 11.7+-1.7 | Clostridiales | 7/13 | 1.0 | .22 | 3 | 0/0/0 | 728 |  |
| 131 | 207 | 2017698 | 17.7+-3.7 | Clostridiales | 8/8 | 1.1 | .22 | 26 | 0/0/0 | 1789 |  |
| 132 | 116 | 2208503 | 26.5+-36.4 | Bacteroidales | 6/8 | 1.0 | .19 | 29 | 2/0/0 | 1903 |  |
| 133 | 36 | 191194 | 19.1+-17.4 | Bacteroidales | 6/8 | 1.0 | .19 | 0 | 1/0/0 | 200 |  |
| 134 | 36 | 227800 | 14.0+-6.9 | Bacteroidales | 13/17 | 2.2 | .19 | 1 | 0/0/0 | 203 |  |
| 135 | 118 | 598820 | 19.5+-25.7 | Bacteroidales | 7/9 | 1.2 | .19 | 2 | 1/0/0 | 587 |  |
| 136 | 94 | 605370 | 17.3+-16.3 | Bacteroidales | 6/10 | 1.0 | .19 | 6 | 0/0/0 | 613 |  |
| 137 | 18 | 108534 | 14.1+-5.0 | Verrucomicrobia | 6/10 | 1.0 | .19 | 1 | 0/0/0 | 102 |  |
| 138 | 172 | 2812254 | 27.2+-21.9 | Butyrivibrio | 6/12 | 1.0 | .19 | 38 | 0/0/0 | 2665 |  |
| 139 | 42 | 1130133 | 38.7+-9.5 | Acholeplasmataceae | 6/17 | 1.0 | .19 | 34 | 2/0/0 | 1067 |  |
| 140 | 180 | 1254256 | 12.5+-3.4 | Ruminococcus | 7/11 | 1.2 | .19 | 21 | 2/0/0 | 1129 |  |
| 141 | 124 | 1128611 | 37.1+-28.5 | Erysipelothrix | 6/17 | 1.0 | .19 | 47 | 3/0/0 | 1109 |  |
| 142 | 320 | 2545832 | 15.8+-5.8 | Bacteroidales | 15/25 | 2.5 | .19 | 34 | 0/0/0 | 2341 |  |
| 143 | 74 | 609222 | 23.3+-19.4 | Butyrivibrio | 6/14 | 1.0 | .19 | 1 | 0/0/0 | 650 |  |
| 144 | 26 | 455832 | 35.5+-5.0 | Verrucomicrobia | 7/9 | 1.2 | .19 | 12 | 0/0/0 | 334 |  |
| 145 | 116 | 1390745 | 12.8+-3.3 | Ruminococcaceae | 6/12 | 1.0 | .19 | 28 | 0/0/0 | 1333 |  |
| 146 | 254 | 3530122 | 54.7+-22.0 | Butyrivibrio | 7/14 | 1.2 | .19 | 30 | 0/0/0 | 3158 |  |
| 147 | 117 | 1426860 | 118.0+-71.2 | Bacteroidales | 7/10 | 1.2 | .19 | 41 | 2/0/0 | 1289 |  |
| 148 | 21 | 51205 | 13.0+-5.6 | Clostridiales | 6/7 | 1.0 | .19 | 0 | 0/0/0 | 65 |  |
| 149 | 17 | 47895 | 62.8+-191.9 | Ruminococcaceae | 6/9 | 1.0 | .19 | 0 | 0/0/0 | 66 |  |
| 150 | 73 | 1233891 | 15.2+-4.1 | Bacteroides | 6/12 | 1.0 | .19 | 24 | 0/0/0 | 1108 |  |
| 151 | 44 | 150009 | 14.9+-13.4 | Bacteroidales | 7/8 | 1.4 | .16 | 5 | 0/0/0 | 164 |  |
| 152 | 29 | 112659 | 30.8+-34.6 | Bacteroidetes | 8/8 | 1.6 | .16 | 2 | 0/0/0 | 104 |  |
| 153 | 19 | 81398 | 15.0+-10.5 | Bacteroidales | 5/6 | 1.0 | .16 | 2 | 0/0/0 | 67 |  |
| 154 | 71 | 233999 | 13.2+-9.2 | Verrucomicrobia | 5/11 | 1.0 | .16 | 2 | 0/0/0 | 276 |  |
| 155 | 130 | 459138 | 10.0+-1.6 | Clostridiales | 5/5 | 1.0 | .16 | 4 | 0/0/0 | 479 |  |
| 156 | 166 | 1733875 | 39.0+-39.5 | Clostridiales | 5/8 | 1.0 | .16 | 23 | 0/0/0 | 1655 |  |
| 157 | 13 | 60789 | 17.5+-11.2 | Ruminococcaceae | 5/8 | 1.0 | .16 | 0 | 0/0/0 | 66 |  |
| 158 | 20 | 91590 | 35.6+-36.6 | Paludibacter | 5/12 | 1.0 | .16 | 3 | 0/0/0 | 89 |  |
| 159 | 97 | 1198045 | 15.9+-4.5 | Clostridiales | 7/11 | 1.4 | .16 | 29 | 0/0/0 | 1109 |  |
| 160 | 83 | 373386 | 12.5+-9.3 | Alphaproteobacteria | 5/5 | 1.0 | .16 | 8 | 0/0/0 | 408 |  |
| 161 | 128 | 620759 | 12.2+-5.9 | Bacteroidales | 5/8 | 1.0 | .16 | 8 | 0/0/0 | 655 |  |
| 162 | 84 | 445002 | 12.9+-3.0 | Treponema | 6/7 | 1.2 | .16 | 5 | 0/0/0 | 390 |  |
| 163 | 224 | 1102419 | 25.2+-21.7 | Bacteroidales | 9/10 | 1.8 | .16 | 2 | 1/0/0 | 1213 |  |
| 164 | 197 | 1300497 | 27.8+-25.1 | Bacteroidales | 5/11 | 1.0 | .16 | 37 | 0/0/0 | 1401 |  |
| 165 | 21 | 192362 | 30.2+-31.8 | Bacteroidales | 5/5 | 1.0 | .16 | 0 | 0/0/0 | 155 |  |
| 166 | 145 | 773994 | 14.5+-12.6 | Ruminococcaceae | 5/6 | 1.0 | .16 | 7 | 1/0/0 | 801 |  |
| 167 | 175 | 1332461 | 16.7+-6.9 | Bacteroidetes | 5/5 | 1.0 | .16 | 23 | 0/0/0 | 1322 |  |
| 168 | 246 | 2356833 | 13.8+-1.9 | Verrucomicrobia | 5/10 | 1.0 | .16 | 26 | 1/0/0 | 2326 |  |
| 169 | 113 | 1175414 | 20.7+-12.8 | Bacteroidales | 5/8 | 1.0 | .16 | 21 | 2/0/0 | 1041 |  |
| 170 | 55 | 752729 | 58.7+-46.8 | Prevotella | 5/13 | 1.0 | .16 | 5 | 1/0/0 | 547 |  |
| 171 | 30 | 73005 | 17.3+-20.6 | Eubacterium | 5/6 | 1.0 | .16 | 0 | 0/0/0 | 77 |  |
| 172 | 54 | 594011 | 17.3+-5.8 | Clostridiales | 5/7 | 1.2 | .12 | 8 | 0/0/0 | 594 |  |
| 173 | 21 | 84382 | 35.7+-29.7 | Bacteroidales | 4/4 | 1.0 | .12 | 0 | 0/0/0 | 108 |  |
| 174 | 21 | 85440 | 30.0+-35.1 | Bacteroidales | 6/6 | 1.5 | .12 | 3 | 0/0/0 | 100 |  |
| 175 | 52 | 165257 | 15.3+-5.7 | Verrucomicrobia | 4/6 | 1.0 | .12 | 1 | 0/0/0 | 172 |  |
| 176 | 60 | 232433 | 16.3+-6.3 | Verrucomicrobia | 4/6 | 1.0 | .12 | 2 | 0/0/0 | 255 |  |
| 177 | 21 | 99851 | 46.5+-55.7 | Ruminococcus | 4/10 | 1.0 | .12 | 9 | 0/0/0 | 130 |  |
| 178 | 41 | 164761 | 12.6+-8.0 | Clostridiales | 4/4 | 1.0 | .12 | 1 | 0/0/0 | 192 |  |
| 179 | 113 | 1989600 | 118.0+-95.4 | Prevotella | 4/11 | 1.0 | .12 | 24 | 2/0/0 | 1766 |  |
| 180 | 52 | 212148 | 45.5+-110.9 | Erysipelothrix | 7/22 | 1.8 | .12 | 0 | 1/0/1 | 262 |  |
| 181 | 44 | 189204 | 14.1+-8.8 | Ruminococcaceae | 4/6 | 1.0 | .12 | 3 | 0/0/0 | 217 |  |
| 182 | 62 | 323717 | 17.7+-14.5 | Cyanobacteria | 4/10 | 1.0 | .12 | 3 | 1/0/0 | 330 |  |
| 183 | 132 | 636442 | 12.4+-2.4 | Clostridiales | 5/5 | 1.2 | .12 | 4 | 1/0/0 | 625 |  |
| 184 | 67 | 362022 | 31.9+-32.1 | Bacteroidales | 4/6 | 1.0 | .12 | 7 | 0/0/0 | 408 |  |
| 185 | 34 | 212730 | 30.4+-20.7 | Euryarchaeota | 16/23 | 1.2 | .12 | 5 | 0/0/0 | 222 |  |
| 186 | 19 | 146149 | 38.4+-41.7 | Paludibacter | 4/9 | 1.0 | .12 | 3 | 0/0/0 | 172 |  |
| 187 | 15 | 141538 | 50.4+-42.7 | Fibrobacter | 4/4 | 1.0 | .12 | 5 | 0/0/0 | 120 |  |
| 188 | 96 | 576607 | 13.2+-2.3 | Ruminococcaceae | 4/5 | 1.0 | .12 | 7 | 1/0/0 | 610 |  |
| 189 | 25 | 174921 | 22.2+-27.8 | Bacteroidales | 13/14 | 3.2 | .12 | 4 | 0/0/0 | 208 |  |
| 190 | 135 | 900481 | 12.3+-2.0 | Ruminococcus | 4/7 | 1.0 | .12 | 10 | 0/0/0 | 888 |  |
| 191 | 72 | 524861 | 16.7+-17.7 | Ethanoligenens | 4/9 | 1.0 | .12 | 14 | 0/0/0 | 537 |  |
| 192 | 54 | 720934 | 50.2+-15.4 | Bacteroidales | 4/5 | 1.0 | .12 | 17 | 0/0/0 | 658 |  |
| 193 | 265 | 1973843 | 18.9+-9.8 | Verrucomicrobia | 4/5 | 1.0 | .12 | 20 | 0/0/0 | 1508 |  |
| 194 | 238 | 1988771 | 16.2+-12.4 | Bacteroidales | 4/4 | 1.0 | .12 | 17 | 0/0/0 | 1809 |  |
| 195 | 102 | 1540800 | 46.9+-17.2 | Bacteroidales | 4/4 | 1.0 | .12 | 15 | 3/0/0 | 1267 |  |
| 196 | 97 | 884558 | 39.2+-32.4 | Acidaminococcus | 5/6 | 1.2 | .12 | 13 | 1/0/0 | 963 |  |
| 197 | 86 | 1103979 | 31.1+-8.2 | Bacteroidales | 4/4 | 1.0 | .12 | 20 | 3/0/0 | 998 |  |
| 198 | 17 | 38717 | 10.7+-2.5 | Clostridiales | 4/4 | 1.0 | .12 | 1 | 0/0/0 | 42 |  |
| 199 | 18 | 44173 | 17.8+-19.2 | Clostridiales | 4/4 | 1.0 | .12 | 2 | 0/0/0 | 72 |  |
| 200 | 244 | 2499010 | 14.9+-4.5 | Treponema | 5/6 | 1.2 | .12 | 32 | 1/0/0 | 2320 |  |
| 201 | 16 | 48706 | 23.8+-15.5 | Bacteroidetes | 4/5 | 1.0 | .12 | 0 | 0/0/0 | 74 |  |
| 202 | 56 | 595151 | 31.3+-20.9 | Euryarchaeota | 15/16 | 1.4 | .10 | 6 | 0/0/0 | 651 |  |
| 203 | 25 | 86135 | 23.1+-19.9 | Bacteroidetes | 3/3 | 1.0 | .09 | 3 | 0/0/0 | 103 |  |
| 204 | 129 | 2521740 | 53.0+-32.3 | Prevotella | 5/7 | 1.7 | .09 | 20 | 3/0/0 | 1887 |  |
| 205 | 28 | 117690 | 25.7+-18.7 | Bacteroidales | 4/5 | 1.3 | .09 | 5 | 0/0/1 | 140 |  |
| 206 | 55 | 204825 | 13.3+-5.4 | Treponema | 6/6 | 2.0 | .09 | 3 | 1/0/0 | 195 |  |
| 207 | 295 | 2846788 | 30.1+-29.9 | Clostridiales | 5/6 | 1.7 | .09 | 29 | 0/0/0 | 2998 |  |
| 208 | 67 | 906169 | 14.6+-3.0 | Clostridiales | 3/3 | 1.0 | .09 | 7 | 0/0/0 | 879 |  |
| 209 | 120 | 430721 | 11.9+-3.7 | Ruminococcus | 3/6 | 1.0 | .09 | 1 | 1/0/0 | 452 |  |
| 210 | 47 | 189341 | 20.4+-31.2 | Bacteroidales | 3/3 | 1.0 | .09 | 0 | 1/0/0 | 185 |  |
| 211 | 83 | 337280 | 13.1+-5.6 | Bacteroidales | 3/3 | 1.0 | .09 | 6 | 0/0/0 | 361 |  |
| 212 | 65 | 260826 | 14.9+-9.9 | Clostridiales | 5/5 | 1.7 | .09 | 3 | 0/0/0 | 274 |  |
| 213 | 48 | 253819 | 12.5+-3.4 | Ruminococcaceae | 3/3 | 1.0 | .09 | 9 | 0/0/0 | 278 |  |
| 214 | 37 | 194519 | 86.5+-439.9 | Clostridiales | 3/7 | 1.0 | .09 | 14 | 0/0/0 | 198 |  |
| 215 | 128 | 517227 | 10.9+-2.1 | Verrucomicrobia | 3/7 | 1.0 | .09 | 8 | 0/0/0 | 609 |  |
| 216 | 93 | 1184743 | 55.0+-22.3 | Clostridiales | 5/5 | 1.7 | .09 | 7 | 0/0/0 | 1057 |  |
| 217 | 100 | 458381 | 13.2+-4.5 | Erysipelothrix | 3/3 | 1.0 | .09 | 3 | 0/0/0 | 536 |  |
| 218 | 69 | 392615 | 27.5+-26.0 | Erysipelothrix | 3/10 | 1.0 | .09 | 3 | 0/0/0 | 443 |  |
| 219 | 205 | 1069023 | 12.0+-2.8 | Treponema | 5/6 | 1.7 | .09 | 13 | 0/0/0 | 1066 |  |
| 220 | 148 | 851289 | 18.7+-35.5 | Clostridiales | 4/4 | 1.3 | .09 | 6 | 0/0/0 | 753 |  |
| 221 | 18 | 951932 | 58.5+-8.9 | Ruminococcaceae | 3/3 | 1.0 | .09 | 18 | 1/0/0 | 855 |  |
| 222 | 79 | 627034 | 96.6+-49.0 | Bacteroidales | 4/4 | 1.3 | .09 | 2 | 0/0/0 | 559 |  |
| 223 | 57 | 350168 | 17.1+-10.0 | Erysipelothrix | 3/4 | 1.0 | .09 | 1 | 0/0/0 | 310 |  |
| 224 | 115 | 761364 | 63.8+-35.4 | Erysipelothrix | 4/7 | 1.3 | .09 | 39 | 0/0/0 | 757 |  |
| 225 | 108 | 2873101 | 29.4+-16.1 | Prevotella | 4/15 | 1.3 | .09 | 15 | 3/0/0 | 2033 |  |
| 226 | 61 | 555853 | 19.0+-14.2 | Bacteroidales | 3/5 | 1.0 | .09 | 4 | 0/0/0 | 494 |  |
| 227 | 88 | 592953 | 27.5+-23.8 | Paludibacter | 3/6 | 1.0 | .09 | 5 | 1/0/0 | 529 |  |
| 228 | 130 | 947448 | 24.3+-19.4 | Bacteroidales | 4/9 | 1.3 | .09 | 14 | 2/0/0 | 1046 |  |
| 229 | 12 | 130095 | 449.0+-553.5 | Treponema | 3/4 | 1.0 | .09 | 5 | 0/0/0 | 144 |  |
| 230 | 178 | 1837935 | 49.4+-32.6 | Paludibacter | 4/8 | 1.3 | .09 | 34 | 1/0/0 | 1576 |  |
| 231 | 74 | 838806 | 69.2+-18.8 | Prevotella | 3/8 | 1.0 | .09 | 5 | 2/0/0 | 678 |  |
| 232 | 67 | 774811 | 21.8+-27.3 | Fibrobacter | 3/3 | 1.0 | .09 | 16 | 0/0/0 | 723 |  |
| 233 | 215 | 1443080 | 17.3+-4.8 | Opitutae | 3/15 | 1.0 | .09 | 18 | 0/0/0 | 1336 |  |
| 234 | 106 | 1155349 | 55.0+-32.6 | Paludibacter | 5/10 | 1.7 | .09 | 29 | 1/0/0 | 1078 |  |
| 235 | 46 | 452283 | 24.1+-18.5 | Clostridiales | 3/3 | 1.0 | .09 | 7 | 0/0/0 | 573 |  |
| 236 | 13 | 27692 | 17.5+-9.9 | Ruminococcaceae | 3/3 | 1.0 | .09 | 2 | 0/0/0 | 41 |  |
| 237 | 22 | 50544 | 14.1+-12.7 | Cyanobacteria | 3/4 | 1.0 | .09 | 4 | 0/0/0 | 77 |  |
| 238 | 20 | 46494 | 61.1+-145.5 | Bacteroidales | 3/3 | 1.0 | .09 | 0 | 0/0/0 | 41 |  |
| 239 | 18 | 42513 | 9.7+-2.2 | Elusimicrobium | 3/4 | 1.0 | .09 | 0 | 0/0/0 | 68 |  |
| 240 | 18 | 45614 | 24.6+-31.2 | Ruminococcus | 3/8 | 1.0 | .09 | 0 | 0/0/0 | 58 |  |
| 241 | 171 | 1870702 | 48.0+-55.9 | Prevotella | 4/8 | 1.3 | .09 | 26 | 2/0/0 | 1836 |  |
| 242 | 38 | 689030 | 62.9+-24.0 | Prevotella | 3/4 | 1.0 | .09 | 14 | 0/0/0 | 557 |  |
| 243 | 11 | 30023 | 24.0+-27.1 | Fibrobacter | 3/3 | 1.0 | .09 | 0 | 0/0/0 | 23 |  |
| 244 | 16 | 42279 | 15.3+-5.7 | Clostridium | 3/6 | 1.0 | .09 | 0 | 0/0/0 | 42 |  |
| 245 | 20 | 66106 | 14.6+-9.6 | Clostridiales | 4/6 | 1.3 | .09 | 1 | 1/0/0 | 71 |  |
| 246 | 17 | 41453 | 27.0+-30.8 | Euryarchaeota | 9/11 | 1.0 | .08 | 0 | 0/0/0 | 45 |  |
| 247 | 79 | 243785 | 11.0+-2.3 | Treponema | 2/2 | 1.0 | .06 | 2 | 1/0/0 | 271 |  |
| 248 | 44 | 156180 | 10.4+-1.8 | Clostridium | 2/2 | 1.0 | .06 | 1 | 0/0/0 | 150 |  |
| 249 | 204 | 2207336 | 30.8+-23.8 | Odoribacter | 2/6 | 1.0 | .06 | 29 | 1/0/0 | 2106 |  |
| 250 | 65 | 210943 | 11.2+-2.6 | Treponema | 2/2 | 1.0 | .06 | 0 | 0/0/0 | 196 |  |
| 251 | 25 | 119591 | 18.9+-11.6 | Bacteroidales | 2/3 | 1.0 | .06 | 4 | 0/0/0 | 130 |  |
| 252 | 24 | 110010 | 20.2+-14.7 | Ruminococcaceae | 4/6 | 2.0 | .06 | 2 | 0/0/0 | 112 |  |
| 253 | 37 | 175857 | 19.4+-17.3 | Bacteroidales | 2/2 | 1.0 | .06 | 1 | 0/0/0 | 115 |  |
| 254 | 62 | 1389756 | 18.8+-1.9 | Paludibacter | 2/7 | 1.0 | .06 | 30 | 2/0/0 | 1287 |  |
| 255 | 59 | 280326 | 19.1+-17.1 | Eubacterium | 2/4 | 1.0 | .06 | 5 | 0/0/0 | 296 |  |
| 256 | 118 | 582824 | 29.5+-30.9 | Bacteroidales | 3/3 | 1.5 | .06 | 3 | 0/0/0 | 678 |  |
| 257 | 57 | 292131 | 12.4+-3.5 | Ruminococcus | 2/2 | 1.0 | .06 | 3 | 0/0/0 | 235 |  |
| 258 | 25 | 141052 | 58.1+-34.0 | Paludibacter | 2/3 | 1.0 | .06 | 0 | 0/0/0 | 85 |  |
| 259 | 51 | 309717 | 21.1+-10.3 | Ruminococcaceae | 2/3 | 1.0 | .06 | 10 | 1/0/0 | 375 |  |
| 260 | 108 | 619435 | 19.4+-10.0 | Verrucomicrobia | 2/2 | 1.0 | .06 | 0 | 0/0/0 | 567 |  |
| 261 | 71 | 518522 | 59.0+-40.6 | Porphyromonas | 2/3 | 1.0 | .06 | 6 | 1/0/0 | 482 |  |
| 262 | 177 | 2573162 | 24.5+-21.7 | Erysipelothrix | 3/6 | 1.5 | .06 | 26 | 0/0/0 | 2364 |  |
| 263 | 93 | 549182 | 88.5+-138.0 | Fibrobacter | 2/3 | 1.0 | .06 | 4 | 0/0/0 | 491 |  |
| 264 | 152 | 873743 | 31.2+-8.4 | Bacteroidales | 4/5 | 2.0 | .06 | 8 | 0/0/0 | 798 |  |
| 265 | 274 | 2480031 | 21.0+-8.8 | Verrucomicrobia | 2/5 | 1.0 | .06 | 19 | 0/0/0 | 2157 |  |
| 266 | 35 | 383857 | 22.0+-14.8 | Erysipelothrix | 2/2 | 1.0 | .06 | 5 | 0/0/0 | 239 |  |
| 267 | 87 | 938206 | 51.1+-19.4 | Bacteroides | 2/3 | 1.0 | .06 | 3 | 0/0/0 | 637 |  |
| 268 | 42 | 474461 | 73.6+-41.1 | Bacteroidales | 2/3 | 1.0 | .06 | 5 | 0/0/0 | 418 |  |
| 269 | 16 | 298121 | 30.3+-9.2 | Ruminococcaceae | 2/2 | 1.0 | .06 | 17 | 0/0/0 | 314 |  |
| 270 | 41 | 456663 | 28.6+-15.2 | Ruminococcaceae | 2/2 | 1.0 | .06 | 6 | 0/0/0 | 498 |  |
| 271 | 120 | 959628 | 32.6+-27.5 | Verrucomicrobia | 2/3 | 1.0 | .06 | 7 | 0/0/0 | 947 |  |
| 272 | 116 | 1522140 | 14.0+-1.5 | Clostridiales | 2/2 | 1.0 | .06 | 14 | 0/0/0 | 1424 |  |
| 273 | 7 | 14180 | 9.9+-2.3 | Clostridiales | 2/2 | 1.0 | .06 | 0 | 0/0/0 | 19 |  |
| 274 | 10 | 21259 | 11.5+-3.5 | Prevotella | 2/3 | 1.0 | .06 | 0 | 0/0/0 | 23 |  |
| 275 | 84 | 1009854 | 14.3+-2.0 | Ruminococcus | 2/4 | 1.0 | .06 | 30 | 0/0/0 | 961 |  |
| 276 | 81 | 1078032 | 29.2+-25.0 | Eubacterium | 3/7 | 1.5 | .06 | 6 | 1/0/0 | 935 |  |
| 277 | 19 | 56229 | 13.4+-3.7 | Odoribacter | 2/3 | 1.0 | .06 | 0 | 0/0/0 | 49 |  |
| 278 | 20 | 55822 | 12.6+-3.4 | Methanocorpusculum | 5/12 | 1.0 | .04 | 2 | 0/0/0 | 73 |  |
| 279 | 15 | 55002 | 13.2+-6.0 | Erysipelothrix | 1/1 | 1.0 | .03 | 0 | 0/0/0 | 49 |  |
| 280 | 21 | 70361 | 23.2+-27.0 | Clostridiales | 1/1 | 1.0 | .03 | 1 | 0/0/0 | 88 |  |
| 281 | 29 | 97269 | 9.9+-1.7 | Butyrivibrio | 1/1 | 1.0 | .03 | 0 | 0/0/0 | 104 |  |
| 282 | 35 | 114420 | 17.8+-17.2 | Cyanobacteria | 1/1 | 1.0 | .03 | 6 | 0/0/0 | 124 |  |
| 283 | 25 | 91383 | 14.8+-15.0 | Erysipelothrix | 1/1 | 1.0 | .03 | 2 | 0/0/0 | 102 |  |
| 284 | 55 | 172896 | 17.0+-20.1 | Clostridiales | 1/1 | 1.0 | .03 | 0 | 0/0/0 | 200 |  |
| 285 | 15 | 54116 | 182.1+-487.5 | Prevotella | 1/1 | 1.0 | .03 | 0 | 0/0/0 | 79 |  |
| 286 | 34 | 117190 | 18.3+-35.5 | Bacteroidales | 1/1 | 1.0 | .03 | 0 | 0/0/0 | 105 |  |
| 287 | 58 | 187795 | 21.6+-35.7 | Bacteroidales | 1/1 | 1.0 | .03 | 2 | 0/0/0 | 201 |  |
| 288 | 45 | 139952 | 15.4+-12.2 | Clostridiales | 1/1 | 1.0 | .03 | 0 | 0/0/0 | 155 |  |
| 289 | 15 | 60440 | 23.6+-20.7 | Porphyromonadaceae | 1/1 | 1.0 | .03 | 0 | 0/0/0 | 42 |  |
| 290 | 16 | 62029 | 30.6+-30.9 | Bacteroidales | 1/1 | 1.0 | .03 | 0 | 0/0/0 | 62 |  |
| 291 | 23 | 84371 | 34.1+-36.8 | Prevotella | 1/1 | 1.0 | .03 | 0 | 0/0/0 | 69 |  |
| 292 | 38 | 125988 | 21.0+-22.0 | Bacteroidales | 1/1 | 1.0 | .03 | 0 | 0/0/0 | 169 |  |
| 293 | 19 | 75133 | 33.7+-28.9 | Bacteroidales | 1/1 | 1.0 | .03 | 1 | 0/0/0 | 71 |  |
| 294 | 22 | 83350 | 13.8+-7.3 | Ruminococcaceae | 1/1 | 1.0 | .03 | 0 | 0/0/0 | 101 |  |
| 295 | 37 | 133265 | 15.7+-17.6 | Clostridiales | 1/1 | 1.0 | .03 | 0 | 0/0/0 | 142 |  |
| 296 | 17 | 69277 | 11.5+-2.9 | Mollicutes | 1/1 | 1.0 | .03 | 0 | 0/0/0 | 99 |  |
| 297 | 35 | 126961 | 16.5+-18.0 | Deinococci | 1/1 | 1.0 | .03 | 23 | 0/0/0 | 163 |  |
| 298 | 69 | 230829 | 14.7+-11.7 | Spirochaetaceae | 1/1 | 1.0 | .03 | 3 | 0/0/0 | 270 |  |
| 299 | 20 | 81550 | 15.9+-9.5 | Bacteroidales | 3/4 | 3.0 | .03 | 0 | 0/0/0 | 94 |  |
| 300 | 23 | 92929 | 16.4+-12.7 | Ethanoligenens | 1/1 | 1.0 | .03 | 1 | 0/0/0 | 120 |  |
| 301 | 27 | 114184 | 30.7+-32.5 | Ethanoligenens | 1/1 | 1.0 | .03 | 0 | 0/0/0 | 110 |  |
| 302 | 73 | 250880 | 17.9+-26.4 | Treponema | 1/1 | 1.0 | .03 | 2 | 0/0/0 | 291 |  |
| 303 | 16 | 70302 | 68.2+-151.3 | Bacteroidales | 1/1 | 1.0 | .03 | 0 | 0/0/0 | 70 |  |
| 304 | 61 | 213002 | 11.4+-2.5 | Prevotella | 1/1 | 1.0 | .03 | 0 | 0/0/0 | 159 |  |
| 305 | 38 | 163619 | 31.0+-28.2 | Prevotella | 1/1 | 1.0 | .03 | 0 | 0/0/0 | 200 |  |
| 306 | 28 | 112004 | 19.3+-9.9 | Bacteroidales | 1/1 | 1.0 | .03 | 0 | 1/0/0 | 117 |  |
| 307 | 52 | 211674 | 66.8+-66.6 | Bacteroidales | 1/1 | 1.0 | .03 | 1 | 0/0/0 | 196 |  |
| 308 | 67 | 253967 | 16.3+-14.1 | Bacteroidales | 1/1 | 1.0 | .03 | 6 | 0/0/0 | 281 |  |
| 309 | 28 | 111487 | 35.6+-64.4 | Porphyromonas | 1/1 | 1.0 | .03 | 5 | 0/0/0 | 145 |  |
| 310 | 40 | 167128 | 43.2+-35.5 | Epsilonproteobacteria | 1/1 | 1.0 | .03 | 24 | 1/0/0 | 180 |  |
| 311 | 25 | 113966 | 66.7+-45.5 | Bacteroidetes | 1/1 | 1.0 | .03 | 3 | 0/0/0 | 95 |  |
| 312 | 27 | 113139 | 16.9+-19.6 | Clostridiales | 1/1 | 1.0 | .03 | 0 | 0/0/0 | 127 |  |
| 313 | 65 | 254157 | 32.5+-31.5 | Coprothermobacter | 1/1 | 1.0 | .03 | 5 | 0/0/0 | 343 |  |
| 314 | 49 | 205751 | 23.7+-20.3 | Paludibacter | 1/1 | 1.0 | .03 | 2 | 0/0/0 | 192 |  |
| 315 | 37 | 152693 | 55.3+-49.7 | Paludibacter | 1/1 | 1.0 | .03 | 3 | 0/0/0 | 171 |  |
| 316 | 28 | 123410 | 23.6+-16.8 | Prevotella | 1/1 | 1.0 | .03 | 0 | 0/0/0 | 92 |  |
| 317 | 28 | 131807 | 24.6+-24.6 | Paludibacter | 1/1 | 1.0 | .03 | 1 | 0/0/0 | 78 |  |
| 318 | 61 | 255846 | 25.4+-23.9 | Coprothermobacter | 1/1 | 1.0 | .03 | 2 | 0/0/0 | 285 |  |
| 319 | 24 | 117021 | 33.1+-37.0 | Erysipelothrix | 2/3 | 2.0 | .03 | 0 | 0/0/0 | 90 |  |
| 320 | 45 | 192444 | 19.4+-18.4 | Prevotella | 2/2 | 2.0 | .03 | 0 | 0/0/0 | 151 |  |
| 321 | 68 | 302493 | 27.0+-32.5 | Bacteroidales | 1/1 | 1.0 | .03 | 2 | 0/0/0 | 330 |  |
| 322 | 14 | 73158 | 14.6+-7.5 | Ruminococcaceae | 2/2 | 2.0 | .03 | 0 | 0/0/0 | 67 |  |
| 323 | 98 | 370518 | 10.7+-2.4 | Clostridiales | 1/1 | 1.0 | .03 | 6 | 0/0/0 | 391 |  |
| 324 | 39 | 166111 | 19.6+-11.2 | Acholeplasmataceae | 1/1 | 1.0 | .03 | 1 | 0/0/0 | 196 |  |
| 325 | 12 | 74261 | 30.3+-24.7 | Verrucomicrobia | 1/1 | 1.0 | .03 | 1 | 0/0/0 | 66 |  |
| 326 | 22 | 101519 | 21.6+-18.6 | Prevotella | 1/1 | 1.0 | .03 | 1 | 0/0/0 | 135 |  |
| 327 | 25 | 134918 | 23.7+-19.5 | Butyrivibrio | 2/3 | 2.0 | .03 | 1 | 0/0/0 | 165 |  |
| 328 | 64 | 296976 | 42.7+-38.1 | Paludibacter | 2/2 | 2.0 | .03 | 1 | 0/0/0 | 271 |  |
| 329 | 54 | 247469 | 38.1+-30.0 | Paludibacter | 1/1 | 1.0 | .03 | 3 | 0/0/0 | 279 |  |
| 330 | 182 | 807761 | 16.0+-17.7 | Thermodesulfobiaceae | 1/1 | 1.0 | .03 | 7 | 0/0/0 | 808 |  |
| 331 | 11 | 70021 | 73.7+-69.9 | Bacteroidales | 1/1 | 1.0 | .03 | 0 | 0/0/0 | 56 |  |
| 332 | 27 | 122423 | 47.2+-98.1 | Bacteroidales | 1/1 | 1.0 | .03 | 0 | 0/0/0 | 69 |  |
| 333 | 31 | 131648 | 12.3+-12.3 | Erysipelothrix | 2/2 | 2.0 | .03 | 0 | 0/0/0 | 144 |  |
| 334 | 48 | 256167 | 17.6+-3.1 | Clostridiales | 1/1 | 1.0 | .03 | 3 | 0/0/0 | 263 |  |
| 335 | 50 | 222485 | 13.4+-10.1 | Clostridiales | 1/1 | 1.0 | .03 | 0 | 0/0/0 | 197 |  |
| 336 | 34 | 193930 | 67.6+-70.6 | Paludibacter | 1/1 | 1.0 | .03 | 1 | 0/0/0 | 185 |  |
| 337 | 122 | 621835 | 18.1+-14.0 | Paludibacter | 1/1 | 1.0 | .03 | 8 | 0/0/0 | 604 |  |
| 338 | 98 | 464208 | 16.4+-9.8 | Chlamydiales | 1/1 | 1.0 | .03 | 5 | 0/0/0 | 473 |  |
| 339 | 127 | 618220 | 14.8+-14.8 | Clostridiales | 1/1 | 1.0 | .03 | 11 | 0/0/0 | 648 |  |
| 340 | 46 | 266198 | 23.2+-20.4 | Chlorobiaceae | 1/1 | 1.0 | .03 | 0 | 0/0/0 | 231 |  |
| 341 | 42 | 203228 | 16.4+-9.9 | Ruminococcaceae | 1/1 | 1.0 | .03 | 4 | 0/0/0 | 230 |  |
| 342 | 82 | 434209 | 17.4+-12.0 | Verrucomicrobia | 1/1 | 1.0 | .03 | 8 | 0/0/0 | 456 |  |
| 343 | 199 | 874516 | 11.7+-2.4 | Treponema | 1/1 | 1.0 | .03 | 7 | 0/0/0 | 860 |  |
| 344 | 197 | 911751 | 15.6+-16.0 | Sphaerochaeta | 1/1 | 1.0 | .03 | 12 | 0/0/0 | 995 |  |
| 345 | 111 | 514439 | 21.1+-24.8 | Eubacterium | 2/5 | 2.0 | .03 | 1 | 0/0/0 | 488 |  |
| 346 | 4 | 23918 | 13.6+-3.2 | Clostridiales | 1/1 | 1.0 | .03 | 0 | 0/0/0 | 42 |  |
| 347 | 75 | 484871 | 42.5+-34.6 | Prevotella | 1/1 | 1.0 | .03 | 3 | 0/0/0 | 443 |  |
| 348 | 49 | 328443 | 28.1+-29.0 | Bacteroides | 1/1 | 1.0 | .03 | 0 | 1/0/0 | 347 |  |
| 349 | 60 | 284080 | 15.0+-22.3 | Butyrivibrio | 1/1 | 1.0 | .03 | 2 | 0/0/0 | 329 |  |
| 350 | 73 | 435395 | 27.1+-28.5 | Bacteroides | 2/2 | 2.0 | .03 | 1 | 2/0/0 | 305 |  |
| 351 | 82 | 457877 | 21.1+-19.7 | Prevotella | 1/1 | 1.0 | .03 | 0 | 1/0/0 | 467 |  |
| 352 | 38 | 215911 | 56.5+-21.8 | Bacteroidales | 1/1 | 1.0 | .03 | 0 | 0/0/0 | 164 |  |
| 353 | 42 | 236757 | 17.5+-16.7 | Bacteroidetes | 1/1 | 1.0 | .03 | 6 | 0/0/0 | 259 |  |
| 354 | 25 | 125055 | 15.9+-5.2 | Bacteroidales | 1/1 | 1.0 | .03 | 0 | 0/0/0 | 76 |  |
| 355 | 42 | 251651 | 25.6+-45.6 | Clostridiales | 1/1 | 1.0 | .03 | 3 | 0/0/0 | 291 |  |
| 356 | 100 | 571461 | 21.5+-16.1 | Cyanobacteria | 1/1 | 1.0 | .03 | 18 | 0/0/0 | 579 |  |
| 357 | 44 | 328573 | 20.3+-6.1 | Clostridiales | 1/1 | 1.0 | .03 | 7 | 0/0/0 | 341 |  |
| 358 | 92 | 467878 | 15.7+-13.9 | Clostridiales | 1/1 | 1.0 | .03 | 4 | 0/0/0 | 506 |  |
| 359 | 108 | 582817 | 15.2+-10.1 | Porphyromonas | 3/3 | 3.0 | .03 | 4 | 1/0/0 | 538 |  |
| 360 | 58 | 420318 | 16.2+-6.8 | Bacteroidales | 1/1 | 1.0 | .03 | 6 | 0/0/0 | 391 |  |
| 361 | 66 | 452739 | 41.5+-49.1 | Bacteroidales | 1/1 | 1.0 | .03 | 11 | 1/0/0 | 450 |  |
| 362 | 60 | 331488 | 12.3+-3.2 | Clostridiales | 1/1 | 1.0 | .03 | 19 | 0/0/0 | 356 |  |
| 363 | 59 | 374306 | 18.8+-17.1 | Geobacillus | 1/1 | 1.0 | .03 | 15 | 0/0/0 | 468 |  |
| 364 | 104 | 456335 | 14.2+-7.5 | Bacteroidales | 1/1 | 1.0 | .03 | 3 | 1/0/0 | 432 |  |
| 365 | 31 | 216430 | 17.2+-8.2 | Fibrobacter | 1/1 | 1.0 | .03 | 1 | 0/0/0 | 201 |  |
| 366 | 53 | 362852 | 17.2+-10.8 | Akkermansia | 1/1 | 1.0 | .03 | 4 | 0/0/0 | 321 |  |
| 367 | 78 | 596937 | 48.0+-44.7 | Porphyromonas | 1/1 | 1.0 | .03 | 8 | 0/0/0 | 542 |  |
| 368 | 103 | 695163 | 29.0+-20.2 | Prevotella | 1/1 | 1.0 | .03 | 7 | 0/0/0 | 650 |  |
| 369 | 13 | 70710 | 67.0+-95.3 | Cyanobacteria | 1/1 | 1.0 | .03 | 15 | 0/0/0 | 76 |  |
| 370 | 128 | 763749 | 16.7+-17.8 | Erysipelothrix | 3/7 | 3.0 | .03 | 22 | 0/0/0 | 786 |  |
| 371 | 79 | 999761 | 28.9+-18.6 | Thermodesulfobiaceae | 1/1 | 1.0 | .03 | 7 | 0/0/0 | 532 |  |
| 372 | 9 | 112171 | 142.7+-24.3 | Campylobacter | 2/2 | 2.0 | .03 | 1 | 0/0/0 | 85 |  |
| 373 | 47 | 398744 | 27.7+-21.1 | Bacteroidales | 1/1 | 1.0 | .03 | 1 | 0/0/0 | 248 |  |
| 374 | 61 | 377947 | 17.9+-9.5 | Clostridiales | 1/1 | 1.0 | .03 | 2 | 0/0/0 | 448 |  |
| 375 | 90 | 648301 | 25.3+-37.3 | Acholeplasmataceae | 1/1 | 1.0 | .03 | 22 | 1/0/0 | 654 |  |
| 376 | 12 | 103135 | 20.5+-16.8 | Clostridium | 1/1 | 1.0 | .03 | 0 | 0/0/0 | 41 |  |
| 377 | 34 | 223655 | 160.5+-67.0 | Campylobacter | 1/1 | 1.0 | .03 | 4 | 1/0/0 | 356 |  |
| 378 | 76 | 531768 | 16.2+-8.5 | Clostridia | 1/1 | 1.0 | .03 | 2 | 0/0/0 | 549 |  |
| 379 | 29 | 223630 | 12.3+-3.2 | Proteobacteria | 1/1 | 1.0 | .03 | 1 | 0/0/0 | 423 |  |
| 380 | 129 | 909240 | 18.8+-7.4 | Gemmatimonas | 1/1 | 1.0 | .03 | 5 | 0/0/0 | 676 |  |
| 381 | 67 | 526389 | 22.3+-24.5 | Bacteroides | 1/1 | 1.0 | .03 | 0 | 0/0/0 | 572 |  |
| 382 | 47 | 392633 | 16.7+-11.2 | Ruminococcaceae | 1/1 | 1.0 | .03 | 3 | 1/0/0 | 323 |  |
| 383 | 154 | 1305216 | 24.1+-19.7 | Odoribacter | 2/3 | 2.0 | .03 | 14 | 0/0/0 | 1190 |  |
| 384 | 64 | 530275 | 15.3+-2.2 | Elusimicrobium | 1/1 | 1.0 | .03 | 10 | 1/0/0 | 467 |  |
| 385 | 178 | 1236718 | 62.6+-24.1 | Prevotella | 2/4 | 2.0 | .03 | 11 | 0/0/0 | 1134 |  |
| 386 | 54 | 552653 | 19.1+-11.0 | Bacteroidales | 2/2 | 2.0 | .03 | 0 | 0/0/0 | 388 |  |
| 387 | 8 | 118402 | 43.4+-26.9 | Ruminococcaceae | 1/1 | 1.0 | .03 | 1 | 0/0/0 | 116 |  |
| 388 | 78 | 831096 | 17.1+-15.1 | Bacteroides | 1/1 | 1.0 | .03 | 21 | 1/0/0 | 731 |  |
| 389 | 15 | 159827 | 39.8+-28.7 | Bacteroidales | 1/1 | 1.0 | .03 | 0 | 0/0/0 | 143 |  |
| 390 | 28 | 291268 | 44.6+-43.3 | Clostridium | 1/1 | 1.0 | .03 | 3 | 0/0/0 | 270 |  |
| 391 | 47 | 402869 | 17.8+-4.7 | Eubacterium | 4/5 | 4.0 | .03 | 1 | 0/0/0 | 279 |  |
| 392 | 22 | 247793 | 17.5+-5.6 | Bacteroidales | 1/1 | 1.0 | .03 | 2 | 1/0/0 | 298 |  |
| 393 | 32 | 343267 | 23.9+-4.9 | Bacteroidetes | 1/1 | 1.0 | .03 | 23 | 0/0/0 | 377 |  |
| 394 | 130 | 1077459 | 18.6+-14.0 | Clostridiales | 1/1 | 1.0 | .03 | 18 | 0/0/0 | 1008 |  |
| 395 | 30 | 315546 | 28.1+-35.3 | Odoribacter | 2/2 | 2.0 | .03 | 7 | 0/0/0 | 339 |  |
| 396 | 69 | 696154 | 18.7+-17.0 | Treponema | 2/16 | 2.0 | .03 | 18 | 0/0/0 | 984 |  |
| 397 | 38 | 516085 | 20.0+-2.5 | Ethanoligenens | 1/1 | 1.0 | .03 | 5 | 0/0/0 | 489 |  |
| 398 | 87 | 714696 | 19.7+-18.7 | Fibrobacter | 2/3 | 2.0 | .03 | 8 | 0/0/0 | 668 |  |
| 399 | 126 | 920565 | 31.4+-38.3 | Bacteroidales | 1/1 | 1.0 | .03 | 8 | 0/0/0 | 982 |  |
| 400 | 23 | 374868 | 67.2+-6.0 | Opitutae | 1/1 | 1.0 | .03 | 2 | 0/0/0 | 249 |  |
| 401 | 31 | 248441 | 15.4+-2.3 | Aquificales | 1/1 | 1.0 | .03 | 0 | 0/0/0 | 653 |  |
| 402 | 16 | 147877 | 13.4+-4.6 | Neorickettsia | 1/1 | 1.0 | .03 | 2 | 0/0/0 | 200 |  |
| 403 | 10 | 150598 | 73.0+-92.7 | Ethanoligenens | 1/1 | 1.0 | .03 | 5 | 0/0/0 | 128 |  |
| 404 | 34 | 603112 | 20.8+-6.4 | Bacteroidales | 1/1 | 1.0 | .03 | 5 | 1/0/0 | 496 |  |
| 405 | 27 | 451004 | 17.3+-2.5 | Bacteroidales | 1/1 | 1.0 | .03 | 3 | 0/0/0 | 287 |  |
| 406 | 7 | 14119 | 13.3+-6.7 | Clostridiales | 1/1 | 1.0 | .03 | 0 | 0/0/0 | 15 |  |
| 407 | 11 | 22888 | 12.9+-5.8 | Erysipelothrix | 1/1 | 1.0 | .03 | 0 | 0/0/0 | 23 |  |
| 408 | 17 | 35704 | 57.1+-132.2 | Bacteroidetes | 1/1 | 1.0 | .03 | 0 | 0/0/0 | 45 |  |
| 409 | 19 | 40406 | 16.9+-14.5 | Odoribacter | 1/1 | 1.0 | .03 | 0 | 0/0/0 | 48 |  |
| 410 | 16 | 34775 | 21.2+-21.0 | Methanocorpusculum | 4/4 | 1.0 | .03 | 0 | 0/0/0 | 40 |  |
| 411 | 20 | 43645 | 53.6+-104.5 | Bacteroidetes | 1/1 | 1.0 | .03 | 1 | 0/0/0 | 46 |  |
| 412 | 18 | 39489 | 10.0+-2.7 | Elusimicrobium | 1/1 | 1.0 | .03 | 0 | 0/0/0 | 48 |  |
| 413 | 23 | 49190 | 17.4+-7.2 | Treponema | 1/1 | 1.0 | .03 | 2 | 1/0/0 | 62 |  |
| 414 | 19 | 41432 | 13.4+-10.1 | Thermodesulfovibrio | 1/1 | 1.0 | .03 | 0 | 0/0/0 | 41 |  |
| 415 | 21 | 47093 | 46.5+-56.5 | Bacteroidales | 1/1 | 1.0 | .03 | 3 | 1/0/0 | 67 |  |
| 416 | 23 | 51960 | 10.0+-3.7 | Clostridiales | 1/1 | 1.0 | .03 | 0 | 0/0/0 | 70 |  |
| 417 | 21 | 49162 | 16.9+-15.4 | Clostridiales | 1/1 | 1.0 | .03 | 2 | 0/0/0 | 58 |  |
| 418 | 20 | 45240 | 13.1+-5.6 | Prevotella | 1/1 | 1.0 | .03 | 0 | 0/0/0 | 53 |  |
| 419 | 21 | 50029 | 44.6+-112.8 | Thermodesulfobiaceae | 1/1 | 1.0 | .03 | 2 | 0/0/0 | 61 |  |
| 420 | 18 | 44673 | 27.5+-39.9 | Bacteroidales | 1/1 | 1.0 | .03 | 1 | 0/0/0 | 45 |  |
| 421 | 21 | 53190 | 41.1+-37.0 | Mycoplasmataceae | 1/1 | 1.0 | .03 | 2 | 1/0/0 | 82 |  |
| 422 | 18 | 44399 | 17.9+-17.9 | Verrucomicrobia | 1/1 | 1.0 | .03 | 1 | 0/0/0 | 51 |  |
| 423 | 33 | 82440 | 12.4+-6.0 | Butyrivibrio | 1/1 | 1.0 | .03 | 5 | 0/0/0 | 111 |  |
| 424 | 15 | 38901 | 24.0+-29.3 | Fibrobacter | 1/1 | 1.0 | .03 | 0 | 0/0/0 | 35 |  |
| 425 | 15 | 38223 | 12.0+-2.7 | Methanocorpusculum | 4/11 | 1.0 | .03 | 2 | 0/0/0 | 54 |  |
| 426 | 17 | 45933 | 12.3+-5.1 | Sphingomonadaceae | 1/1 | 1.0 | .03 | 0 | 0/0/0 | 58 |  |
| 427 | 18 | 46808 | 25.6+-26.3 | Prevotella | 1/1 | 1.0 | .03 | 0 | 0/0/0 | 47 |  |
| 428 | 31 | 80733 | 52.3+-81.1 | Bacteroidales | 1/1 | 1.0 | .03 | 0 | 0/0/0 | 85 |  |
| 429 | 21 | 59173 | 18.4+-20.6 | Bacteroidales | 1/1 | 1.0 | .03 | 0 | 0/0/0 | 62 |  |
| 430 | 27 | 75273 | 16.9+-10.9 | Clostridiales | 1/1 | 1.0 | .03 | 12 | 1/0/0 | 106 |  |
| 431 | 27 | 80041 | 20.5+-12.8 | Erysipelothrix | 1/1 | 1.0 | .03 | 0 | 0/0/0 | 119 |  |
| 432 | 38 | 101090 | 14.4+-13.4 | Erysipelothrix | 1/1 | 1.0 | .03 | 0 | 0/0/0 | 138 |  |
| 433 | 29 | 85826 | 15.7+-11.5 | Erysipelothrix | 1/1 | 1.0 | .03 | 3 | 0/0/0 | 98 |  |
| 434 | 21 | 61899 | 13.1+-7.3 | Butyrivibrio | 2/2 | 2.0 | .03 | 0 | 0/0/0 | 51 |  |
| 435 | 35 | 103949 | 11.1+-2.7 | Treponema | 1/1 | 1.0 | .03 | 2 | 0/0/0 | 110 |  |
| 436 | 31 | 92117 | 19.0+-18.6 | Bacteroidetes | 1/1 | 1.0 | .03 | 1 | 0/0/0 | 142 |  |
| 437 | 20 | 61257 | 13.3+-2.7 | Bacteroidetes | 1/1 | 1.0 | .03 | 1 | 0/0/0 | 77 |  |
| 438 | 28 | 82538 | 36.9+-45.8 | Paludibacter | 1/1 | 1.0 | .03 | 0 | 2/0/0 | 94 |  |
| 439 | 33 | 100352 | 14.8+-8.1 | Clostridiales | 2/3 | 2.0 | .03 | 1 | 0/0/0 | 120 |  |
| 440 | 81 | 921033 | 43.6+-43.2 | Bacteroidales | 1/1 | 1.0 | .03 | 4 | 2/0/0 | 813 |  |
| 441 | 22 | 68109 | 36.1+-75.7 | Odoribacter | 1/1 | 1.0 | .03 | 1 | 0/0/0 | 70 |  |
| 442 | 35 | 106313 | 42.8+-55.8 | Bacteroidetes | 1/1 | 1.0 | .03 | 0 | 0/0/0 | 96 |  |
| 443 | 33 | 98598 | 15.3+-8.9 | Treponema | 1/1 | 1.0 | .03 | 0 | 0/0/0 | 116 |  |
| 444 | 96 | 268762 | 10.2+-3.6 | Clostridium | 1/1 | 1.0 | .03 | 0 | 0/0/0 | 326 |  |
| 445 | 35 | 110051 | 15.1+-10.0 | Porphyromonas | 1/1 | 1.0 | .03 | 1 | 0/0/0 | 132 |  |
| 446 | 31 | 97080 | 19.4+-16.0 | Heliobacterium | 1/1 | 1.0 | .03 | 7 | 0/0/0 | 134 |  |
| 447 | 24 | 80176 | 29.3+-40.1 | Bacteroidales | 1/1 | 1.0 | .03 | 0 | 0/0/0 | 89 |  |
| 448 | 34 | 104977 | 12.0+-8.2 | Erysipelothrix | 1/1 | 1.0 | .03 | 0 | 0/0/0 | 118 |  |
| 449 | 28 | 94891 | 26.0+-24.4 | Porphyromonas | 1/1 | 1.0 | .03 | 5 | 0/0/0 | 116 |  |
| 450 | 83 | 655134 | 13.1+-7.3 | Archaea | 4/6 | 1.3 | .02 | 11 | 0/0/0 | 1290 |  |
| 451 | 8 | 16138 | 10.5+-4.8 | Methanocorpusculum | 3/4 | 1.0 | .02 | 0 | 0/0/0 | 22 |  |
| 452 | 31 | 111344 | 25.2+-37.0 | Methanocorpusculum | 2/3 | 1.0 | .01 | 5 | 0/0/0 | 135 |  |
| 453 | 28 | 124114 | 24.3+-25.5 | Methanocorpusculum | 2/2 | 1.0 | .01 | 0 | 1/0/0 | 155 |  |
| 454 | 21 | 71587 | 40.2+-36.4 | - | 0/0 | 0 | - | 0 | 0/0/0 | 70 |  |
| 455 | 31 | 100450 | 17.8+-17.1 | - | 0/0 | 0 | - | 0 | 0/0/0 | 70 |  |
| 456 | 24 | 81438 | 16.5+-6.0 | - | 0/0 | 0 | - | 0 | 0/0/0 | 64 |  |
| 457 | 42 | 134342 | 18.8+-19.6 | - | 0/0 | 0 | - | 4 | 0/0/0 | 142 |  |
| 458 | 35 | 99131 | 9.9+-3.5 | - | 0/0 | 0 | - | 2 | 0/0/0 | 98 |  |
| 459 | 15 | 50444 | 32.3+-43.7 | - | 0/0 | 0 | - | 3 | 1/0/0 | 33 |  |
| 460 | 25 | 81317 | 13.1+-6.5 | - | 0/0 | 0 | - | 0 | 0/0/0 | 132 |  |
| 461 | 14 | 45376 | 17.0+-5.7 | - | 0/0 | 0 | - | 3 | 0/0/0 | 68 |  |
| 462 | 34 | 116573 | 16.3+-10.0 | - | 0/0 | 0 | - | 0 | 0/0/0 | 135 |  |
| 463 | 18 | 63012 | 24.6+-20.7 | - | 0/0 | 0 | - | 5 | 0/0/0 | 84 |  |
| 464 | 15 | 47307 | 14.0+-7.6 | - | 0/0 | 0 | - | 0 | 0/0/0 | 36 |  |
| 465 | 26 | 80763 | 25.1+-24.8 | - | 0/0 | 0 | - | 0 | 0/0/0 | 100 |  |
| 466 | 27 | 88528 | 62.1+-229.2 | - | 0/0 | 0 | - | 0 | 0/0/0 | 93 |  |
| 467 | 51 | 155430 | 17.9+-24.2 | - | 0/0 | 0 | - | 0 | 0/0/0 | 205 |  |
| 468 | 34 | 113993 | 28.8+-54.8 | - | 0/0 | 0 | - | 5 | 0/0/0 | 122 |  |
| 469 | 16 | 51481 | 25.1+-18.0 | - | 0/0 | 0 | - | 0 | 0/0/0 | 40 |  |
| 470 | 19 | 66117 | 46.1+-55.3 | - | 0/0 | 0 | - | 3 | 1/0/1 | 104 |  |
| 471 | 36 | 120876 | 23.8+-29.4 | - | 0/0 | 0 | - | 1 | 1/0/0 | 153 |  |
| 472 | 32 | 104694 | 24.3+-16.4 | - | 0/0 | 0 | - | 0 | 0/0/0 | 96 |  |
| 473 | 37 | 125041 | 50.0+-94.6 | - | 0/0 | 0 | - | 0 | 0/0/0 | 153 |  |
| 474 | 46 | 149623 | 15.5+-7.6 | - | 0/0 | 0 | - | 1 | 0/0/0 | 135 |  |
| 475 | 27 | 88919 | 19.3+-34.6 | - | 0/0 | 0 | - | 1 | 0/0/0 | 75 |  |
| 476 | 25 | 87369 | 17.2+-11.1 | - | 0/0 | 0 | - | 5 | 0/0/0 | 105 |  |
| 477 | 9 | 29324 | 12.1+-6.1 | - | 0/0 | 0 | - | 0 | 0/0/0 | 40 |  |
| 478 | 17 | 63849 | 29.7+-29.7 | - | 0/0 | 0 | - | 2 | 0/0/0 | 70 |  |
| 479 | 15 | 49756 | 32.3+-56.1 | - | 0/0 | 0 | - | 2 | 0/0/0 | 82 |  |
| 480 | 31 | 100218 | 13.5+-5.3 | - | 0/0 | 0 | - | 0 | 0/0/0 | 72 |  |
| 481 | 48 | 148510 | 14.2+-11.6 | - | 0/0 | 0 | - | 2 | 0/0/0 | 201 |  |
| 482 | 30 | 100230 | 14.2+-7.5 | - | 0/0 | 0 | - | 2 | 1/0/0 | 122 |  |
| 483 | 19 | 65424 | 31.8+-30.4 | - | 0/0 | 0 | - | 0 | 0/0/0 | 44 |  |
| 484 | 32 | 519454 | 16.0+-1.5 | - | 0/0 | 0 | - | 8 | 0/0/0 | 409 |  |
| 485 | 16 | 49810 | 10.6+-2.1 | - | 0/0 | 0 | - | 0 | 0/0/0 | 69 |  |
| 486 | 30 | 105683 | 15.4+-10.9 | - | 0/0 | 0 | - | 0 | 0/0/0 | 142 |  |
| 487 | 1 | 4229 | 30.8+-0.0 | - | 0/0 | 0 | - | 0 | 0/0/0 | 4 |  |
| 488 | 31 | 108995 | 19.9+-14.0 | - | 0/0 | 0 | - | 1 | 0/0/0 | 124 |  |
| 489 | 23 | 83101 | 22.2+-27.7 | - | 0/0 | 0 | - | 0 | 0/0/0 | 87 |  |
| 490 | 39 | 128029 | 22.6+-17.6 | - | 0/0 | 0 | - | 0 | 0/0/0 | 173 |  |
| 491 | 22 | 81529 | 27.5+-28.6 | - | 0/0 | 0 | - | 1 | 0/0/0 | 115 |  |
| 492 | 14 | 49463 | 17.4+-10.0 | - | 0/0 | 0 | - | 0 | 0/0/0 | 29 |  |
| 493 | 39 | 128056 | 22.3+-31.0 | - | 0/0 | 0 | - | 2 | 0/0/0 | 190 |  |
| 494 | 114 | 1462516 | 43.0+-36.2 | - | 0/0 | 0 | - | 9 | 3/0/0 | 1299 |  |
| 495 | 15 | 50803 | 20.5+-14.2 | - | 0/0 | 0 | - | 0 | 0/0/0 | 55 |  |
| 496 | 21 | 74225 | 13.3+-3.9 | - | 0/0 | 0 | - | 0 | 0/0/0 | 27 |  |
| 497 | 35 | 117069 | 18.5+-27.2 | - | 0/0 | 0 | - | 2 | 0/0/0 | 127 |  |
| 498 | 29 | 104458 | 18.6+-9.5 | - | 0/0 | 0 | - | 0 | 0/0/0 | 113 |  |
| 499 | 91 | 276987 | 10.7+-2.1 | - | 0/0 | 0 | - | 3 | 0/0/0 | 329 |  |
| 500 | 21 | 75460 | 21.0+-16.3 | - | 0/0 | 0 | - | 0 | 0/0/0 | 61 |  |
| 501 | 37 | 128181 | 14.6+-10.8 | - | 0/0 | 0 | - | 2 | 0/0/0 | 181 |  |
| 502 | 20 | 70112 | 23.4+-18.2 | - | 0/0 | 0 | - | 0 | 0/0/0 | 91 |  |
| 503 | 21 | 75659 | 56.7+-49.0 | - | 0/0 | 0 | - | 0 | 0/0/0 | 88 |  |
| 504 | 14 | 51056 | 13.9+-6.7 | - | 0/0 | 0 | - | 2 | 0/0/0 | 52 |  |
| 505 | 39 | 137638 | 17.6+-15.3 | - | 0/0 | 0 | - | 13 | 0/0/0 | 178 |  |
| 506 | 33 | 106941 | 23.2+-20.4 | - | 0/0 | 0 | - | 0 | 0/0/0 | 122 |  |
| 507 | 103 | 317456 | 11.3+-6.7 | - | 0/0 | 0 | - | 5 | 0/0/0 | 354 |  |
| 508 | 25 | 95009 | 14.6+-20.2 | - | 0/0 | 0 | - | 0 | 0/0/0 | 94 |  |
| 509 | 64 | 209740 | 23.2+-26.4 | - | 0/0 | 0 | - | 2 | 1/0/0 | 233 |  |
| 510 | 29 | 103364 | 15.7+-4.6 | - | 0/0 | 0 | - | 1 | 0/0/0 | 117 |  |
| 511 | 42 | 146391 | 16.3+-7.8 | - | 0/0 | 0 | - | 3 | 0/0/0 | 195 |  |
| 512 | 7 | 24301 | 12.7+-1.8 | - | 0/0 | 0 | - | 0 | 0/0/0 | 39 |  |
| 513 | 19 | 59105 | 22.9+-24.4 | - | 0/0 | 0 | - | 0 | 0/0/0 | 67 |  |
| 514 | 21 | 75349 | 41.6+-56.6 | - | 0/0 | 0 | - | 5 | 0/0/0 | 109 |  |
| 515 | 22 | 78106 | 11.5+-5.3 | - | 0/0 | 0 | - | 0 | 0/0/0 | 50 |  |
| 516 | 23 | 83836 | 20.9+-13.5 | - | 0/0 | 0 | - | 16 | 0/0/0 | 103 |  |
| 517 | 22 | 79672 | 14.6+-12.7 | - | 0/0 | 0 | - | 0 | 0/0/0 | 80 |  |
| 518 | 22 | 84565 | 14.3+-5.5 | - | 0/0 | 0 | - | 2 | 0/0/0 | 101 |  |
| 519 | 28 | 102143 | 28.6+-34.7 | - | 0/0 | 0 | - | 0 | 0/0/0 | 49 |  |
| 520 | 44 | 162106 | 32.1+-30.5 | - | 0/0 | 0 | - | 4 | 1/0/0 | 202 |  |
| 521 | 30 | 88665 | 19.4+-7.6 | - | 0/0 | 0 | - | 6 | 1/0/0 | 117 |  |
| 522 | 42 | 145293 | 22.0+-18.8 | Euryarchaeota | 1/1 | 1.0 | 0 | 4 | 0/0/0 | 155 |  |
| 523 | 32 | 119526 | 17.6+-13.7 | - | 0/0 | 0 | - | 0 | 0/0/0 | 113 |  |
| 524 | 6 | 209442 | 18.6+-0.9 | - | 0/0 | 0 | - | 3 | 0/0/0 | 221 |  |
| 525 | 20 | 75662 | 29.1+-38.0 | - | 0/0 | 0 | - | 0 | 0/0/0 | 70 |  |
| 526 | 53 | 170067 | 16.6+-17.9 | - | 0/0 | 0 | - | 2 | 0/0/0 | 175 |  |
| 527 | 23 | 86872 | 15.9+-12.8 | - | 0/0 | 0 | - | 2 | 0/0/0 | 74 |  |
| 528 | 19 | 70748 | 36.1+-69.2 | - | 0/0 | 0 | - | 1 | 0/0/0 | 86 |  |
| 529 | 24 | 92431 | 16.8+-9.2 | - | 0/0 | 0 | - | 5 | 0/0/0 | 102 |  |
| 530 | 24 | 92938 | 15.7+-8.0 | - | 0/0 | 0 | - | 0 | 0/0/0 | 96 |  |
| 531 | 17 | 60211 | 14.2+-4.0 | - | 0/0 | 0 | - | 0 | 0/0/0 | 49 |  |
| 532 | 20 | 78846 | 49.5+-116.4 | - | 0/0 | 0 | - | 2 | 0/0/0 | 111 |  |
| 533 | 22 | 87089 | 22.4+-27.9 | - | 0/0 | 0 | - | 0 | 0/0/0 | 95 |  |
| 534 | 23 | 89102 | 13.4+-5.1 | - | 0/0 | 0 | - | 0 | 0/0/0 | 82 |  |
| 535 | 93 | 279135 | 10.2+-1.8 | - | 0/0 | 0 | - | 0 | 0/0/0 | 316 |  |
| 536 | 32 | 122790 | 34.7+-35.7 | - | 0/0 | 0 | - | 6 | 0/0/0 | 152 |  |
| 537 | 31 | 111340 | 16.4+-7.7 | - | 0/0 | 0 | - | 2 | 0/0/0 | 136 |  |
| 538 | 32 | 112371 | 16.4+-16.7 | - | 0/0 | 0 | - | 0 | 0/0/0 | 104 |  |
| 539 | 24 | 87385 | 12.6+-5.8 | - | 0/0 | 0 | - | 1 | 0/0/0 | 99 |  |
| 540 | 40 | 149313 | 19.0+-26.1 | - | 0/0 | 0 | - | 0 | 0/0/0 | 173 |  |
| 541 | 41 | 154638 | 29.8+-100.8 | - | 0/0 | 0 | - | 3 | 0/0/0 | 174 |  |
| 542 | 11 | 38876 | 128.0+-263.4 | - | 0/0 | 0 | - | 3 | 0/0/0 | 44 |  |
| 543 | 47 | 151332 | 15.4+-5.0 | - | 0/0 | 0 | - | 1 | 1/0/0 | 170 |  |
| 544 | 16 | 63420 | 17.6+-12.9 | - | 0/0 | 0 | - | 0 | 0/0/0 | 62 |  |
| 545 | 27 | 105981 | 17.5+-7.6 | - | 0/0 | 0 | - | 1 | 0/0/0 | 76 |  |
| 546 | 24 | 78717 | 16.2+-17.8 | - | 0/0 | 0 | - | 3 | 0/0/0 | 92 |  |
| 547 | 34 | 118491 | 17.5+-17.0 | - | 0/0 | 0 | - | 11 | 0/0/0 | 138 |  |
| 548 | 50 | 180270 | 17.4+-13.0 | - | 0/0 | 0 | - | 1 | 0/0/0 | 136 |  |
| 549 | 31 | 107456 | 16.1+-9.8 | - | 0/0 | 0 | - | 0 | 0/0/0 | 70 |  |
| 550 | 17 | 60195 | 13.3+-6.0 | - | 0/0 | 0 | - | 0 | 0/0/0 | 32 |  |
| 551 | 13 | 52363 | 84.9+-60.4 | - | 0/0 | 0 | - | 1 | 0/0/0 | 70 |  |
| 552 | 24 | 98178 | 18.7+-16.9 | - | 0/0 | 0 | - | 0 | 0/0/0 | 105 |  |
| 553 | 34 | 113965 | 15.9+-10.0 | - | 0/0 | 0 | - | 12 | 1/0/0 | 170 |  |
| 554 | 20 | 85605 | 16.1+-11.3 | - | 0/0 | 0 | - | 1 | 0/0/0 | 121 |  |
| 555 | 16 | 60870 | 18.7+-19.2 | - | 0/0 | 0 | - | 5 | 0/0/0 | 66 |  |
| 556 | 6 | 102834 | 46.2+-18.2 | Euryarchaeota | 1/1 | 1.0 | 0 | 1 | 0/0/0 | 202 |  |
| 557 | 20 | 78961 | 18.2+-16.1 | - | 0/0 | 0 | - | 0 | 0/0/0 | 72 |  |
| 558 | 5 | 23540 | 14.2+-4.6 | - | 0/0 | 0 | - | 0 | 0/0/0 | 22 |  |
| 559 | 36 | 129185 | 94.2+-69.7 | Candidatus Azobacteroides pseudotrichonymphae | 1/1 | 1.0 | 0 | 0 | 1/0/0 | 143 |  |
| 560 | 25 | 97969 | 27.5+-24.5 | - | 0/0 | 0 | - | 0 | 0/0/0 | 104 |  |
| 561 | 59 | 209240 | 18.6+-26.2 | - | 0/0 | 0 | - | 12 | 0/0/0 | 253 |  |
| 562 | 25 | 98358 | 19.3+-18.1 | - | 0/0 | 0 | - | 2 | 0/0/0 | 92 |  |
| 563 | 43 | 149311 | 23.5+-44.2 | - | 0/0 | 0 | - | 0 | 0/0/0 | 256 |  |
| 564 | 51 | 184978 | 21.8+-32.6 | - | 0/0 | 0 | - | 0 | 0/0/0 | 199 |  |
| 565 | 13 | 53502 | 28.7+-29.7 | - | 0/0 | 0 | - | 0 | 0/0/0 | 74 |  |
| 566 | 94 | 1803266 | 32.2+-20.7 | - | 0/0 | 0 | - | 18 | 5/0/0 | 1527 |  |
| 567 | 19 | 81357 | 39.7+-45.0 | - | 0/0 | 0 | - | 0 | 0/0/0 | 80 |  |
| 568 | 17 | 71191 | 150.7+-490.3 | - | 0/0 | 0 | - | 0 | 0/0/0 | 95 |  |
| 569 | 37 | 147778 | 32.6+-36.3 | - | 0/0 | 0 | - | 0 | 0/0/0 | 180 |  |
| 570 | 34 | 121162 | 25.8+-19.7 | - | 0/0 | 0 | - | 2 | 0/0/0 | 137 |  |
| 571 | 76 | 254438 | 14.4+-4.2 | - | 0/0 | 0 | - | 0 | 0/0/0 | 290 |  |
| 572 | 70 | 247053 | 28.0+-34.5 | - | 0/0 | 0 | - | 9 | 0/0/0 | 288 |  |
| 573 | 21 | 93079 | 20.8+-17.6 | - | 0/0 | 0 | - | 1 | 0/0/0 | 106 |  |
| 574 | 27 | 91830 | 11.2+-1.9 | Euryarchaeota | 1/1 | 1.0 | 0 | 6 | 0/0/0 | 106 |  |
| 575 | 26 | 105788 | 15.0+-13.7 | - | 0/0 | 0 | - | 0 | 0/0/0 | 134 |  |
| 576 | 13 | 56748 | 23.3+-24.3 | - | 0/0 | 0 | - | 0 | 0/0/0 | 60 |  |
| 577 | 5 | 18044 | 11.6+-2.3 | - | 0/0 | 0 | - | 0 | 0/0/0 | 23 |  |
| 578 | 40 | 139069 | 23.8+-25.2 | - | 0/0 | 0 | - | 0 | 0/0/0 | 157 |  |
| 579 | 17 | 56963 | 13.7+-5.8 | - | 0/0 | 0 | - | 0 | 0/0/0 | 46 |  |
| 580 | 49 | 168390 | 14.4+-8.7 | - | 0/0 | 0 | - | 0 | 0/0/0 | 173 |  |
| 581 | 28 | 125294 | 21.1+-21.6 | - | 0/0 | 0 | - | 3 | 0/0/0 | 133 |  |
| 582 | 44 | 170601 | 13.0+-6.0 | - | 0/0 | 0 | - | 4 | 0/0/0 | 207 |  |
| 583 | 45 | 165607 | 18.7+-28.2 | - | 0/0 | 0 | - | 2 | 0/0/0 | 209 |  |
| 584 | 22 | 102395 | 31.2+-38.7 | - | 0/0 | 0 | - | 2 | 0/0/0 | 95 |  |
| 585 | 37 | 141438 | 21.7+-17.0 | - | 0/0 | 0 | - | 0 | 0/0/0 | 166 |  |
| 586 | 20 | 84921 | 12.7+-2.5 | - | 0/0 | 0 | - | 0 | 0/0/0 | 75 |  |
| 587 | 30 | 134176 | 24.9+-29.6 | - | 0/0 | 0 | - | 4 | 0/0/0 | 152 |  |
| 588 | 29 | 97938 | 116.9+-175.8 | - | 0/0 | 0 | - | 12 | 10/6/22 | 65 |  |
| 589 | 3 | 12982 | 13.5+-1.3 | - | 0/0 | 0 | - | 0 | 0/0/0 | 22 |  |
| 590 | 33 | 119302 | 15.2+-18.4 | - | 0/0 | 0 | - | 0 | 0/0/0 | 192 |  |
| 591 | 13 | 46875 | 17.2+-9.2 | - | 0/0 | 0 | - | 2 | 0/0/0 | 87 |  |
| 592 | 23 | 91869 | 23.1+-14.4 | - | 0/0 | 0 | - | 0 | 0/0/0 | 49 |  |
| 593 | 34 | 129780 | 15.0+-23.0 | - | 0/0 | 0 | - | 0 | 0/0/0 | 124 |  |
| 594 | 48 | 187676 | 32.2+-45.1 | - | 0/0 | 0 | - | 1 | 1/0/0 | 237 |  |
| 595 | 24 | 96114 | 19.4+-17.2 | - | 0/0 | 0 | - | 0 | 0/0/0 | 84 |  |
| 596 | 77 | 276150 | 14.2+-4.8 | - | 0/0 | 0 | - | 1 | 0/0/0 | 269 |  |
| 597 | 22 | 91934 | 30.0+-30.0 | - | 0/0 | 0 | - | 3 | 0/0/0 | 133 |  |
| 598 | 14 | 55704 | 343.5+-956.9 | - | 0/0 | 0 | - | 0 | 0/0/0 | 64 |  |
| 599 | 32 | 143699 | 35.1+-49.0 | - | 0/0 | 0 | - | 2 | 0/0/0 | 144 |  |
| 600 | 67 | 190791 | 10.0+-3.4 | - | 0/0 | 0 | - | 0 | 0/0/0 | 214 |  |
| 601 | 29 | 128265 | 40.5+-52.0 | - | 0/0 | 0 | - | 3 | 1/0/0 | 148 |  |
| 602 | 29 | 126046 | 18.9+-11.5 | - | 0/0 | 0 | - | 2 | 0/0/0 | 136 |  |
| 603 | 54 | 158481 | 48.0+-39.8 | - | 0/0 | 0 | - | 50 | 8/7/34 | 212 |  |
| 604 | 18 | 61744 | 15.9+-9.7 | - | 0/0 | 0 | - | 2 | 0/0/0 | 80 |  |
| 605 | 16 | 70900 | 25.1+-23.9 | - | 0/0 | 0 | - | 0 | 0/0/0 | 70 |  |
| 606 | 77 | 297623 | 14.6+-8.6 | - | 0/0 | 0 | - | 2 | 2/0/0 | 331 |  |
| 607 | 74 | 266437 | 15.5+-10.3 | Methanocorpusculum | 1/1 | 1.0 | 0 | 2 | 0/0/0 | 324 |  |
| 608 | 97 | 378458 | 17.3+-7.3 | - | 0/0 | 0 | - | 6 | 0/0/0 | 416 |  |
| 609 | 44 | 164081 | 11.2+-2.1 | - | 0/0 | 0 | - | 3 | 0/0/0 | 163 |  |
| 610 | 14 | 62448 | 53.0+-102.4 | - | 0/0 | 0 | - | 0 | 0/0/0 | 93 |  |
| 611 | 45 | 156581 | 18.8+-15.6 | - | 0/0 | 0 | - | 4 | 0/0/0 | 182 |  |
| 612 | 89 | 323605 | 17.1+-9.4 | - | 0/0 | 0 | - | 0 | 0/0/0 | 231 |  |
| 613 | 169 | 2383382 | 31.5+-15.9 | - | 0/0 | 0 | - | 76 | 0/0/0 | 2267 |  |
| 614 | 17 | 71988 | 122.7+-22.6 | - | 0/0 | 0 | - | 0 | 0/0/0 | 109 |  |
| 615 | 44 | 184777 | 19.3+-10.6 | - | 0/0 | 0 | - | 1 | 0/0/0 | 92 |  |
| 616 | 36 | 148459 | 19.5+-24.0 | - | 0/0 | 0 | - | 0 | 0/0/0 | 135 |  |
| 617 | 46 | 1090874 | 30.0+-3.1 | - | 0/0 | 0 | - | 22 | 0/0/0 | 984 |  |
| 618 | 17 | 84732 | 47.9+-49.2 | - | 0/0 | 0 | - | 3 | 0/0/0 | 87 |  |
| 619 | 35 | 157342 | 18.4+-9.7 | - | 0/0 | 0 | - | 0 | 1/0/0 | 163 |  |
| 620 | 59 | 238942 | 13.2+-7.7 | - | 0/0 | 0 | - | 2 | 1/0/0 | 279 |  |
| 621 | 20 | 94316 | 34.7+-32.5 | - | 0/0 | 0 | - | 0 | 0/0/0 | 89 |  |
| 622 | 55 | 205977 | 12.2+-2.6 | - | 0/0 | 0 | - | 0 | 0/0/0 | 405 |  |
| 623 | 31 | 141192 | 20.0+-18.8 | - | 0/0 | 0 | - | 1 | 0/0/0 | 117 |  |
| 624 | 10 | 49562 | 61.1+-21.5 | - | 0/0 | 0 | - | 0 | 0/0/0 | 62 |  |
| 625 | 50 | 192893 | 11.2+-1.9 | - | 0/0 | 0 | - | 1 | 0/0/0 | 195 |  |
| 626 | 24 | 111963 | 13.8+-5.2 | - | 0/0 | 0 | - | 4 | 0/0/0 | 99 |  |
| 627 | 12 | 56527 | 30.6+-80.5 | - | 0/0 | 0 | - | 0 | 0/0/0 | 83 |  |
| 628 | 18 | 92531 | 38.7+-51.9 | - | 0/0 | 0 | - | 1 | 0/0/0 | 90 |  |
| 629 | 32 | 147570 | 13.1+-4.2 | - | 0/0 | 0 | - | 0 | 0/2/0 | 177 |  |
| 630 | 48 | 202598 | 48.9+-50.0 | - | 0/0 | 0 | - | 0 | 0/0/0 | 238 |  |
| 631 | 6 | 23145 | 12.5+-1.9 | - | 0/0 | 0 | - | 0 | 0/0/0 | 8 |  |
| 632 | 18 | 86216 | 19.1+-8.5 | - | 0/0 | 0 | - | 9 | 1/0/0 | 105 |  |
| 633 | 61 | 265991 | 18.2+-24.8 | - | 0/0 | 0 | - | 0 | 0/0/0 | 287 |  |
| 634 | 23 | 111374 | 26.2+-24.6 | - | 0/0 | 0 | - | 0 | 0/0/0 | 96 |  |
| 635 | 27 | 106709 | 28.2+-42.5 | - | 0/0 | 0 | - | 0 | 0/0/0 | 73 |  |
| 636 | 13 | 59570 | 14.3+-5.5 | - | 0/0 | 0 | - | 0 | 0/0/0 | 30 |  |
| 637 | 31 | 137628 | 22.1+-18.7 | - | 0/0 | 0 | - | 0 | 1/0/0 | 175 |  |
| 638 | 39 | 154395 | 17.8+-12.2 | - | 0/0 | 0 | - | 3 | 0/0/0 | 188 |  |
| 639 | 16 | 73589 | 16.9+-6.2 | - | 0/0 | 0 | - | 0 | 0/0/0 | 78 |  |
| 640 | 15 | 62976 | 13.3+-6.9 | - | 0/0 | 0 | - | 1 | 0/0/0 | 103 |  |
| 641 | 21 | 81225 | 25.5+-19.1 | - | 0/0 | 0 | - | 1 | 0/0/0 | 101 |  |
| 642 | 23 | 110593 | 20.3+-15.6 | - | 0/0 | 0 | - | 1 | 0/0/0 | 165 |  |
| 643 | 24 | 116254 | 52.7+-46.0 | - | 0/0 | 0 | - | 6 | 0/0/0 | 119 |  |
| 644 | 47 | 196264 | 26.6+-35.8 | - | 0/0 | 0 | - | 1 | 0/0/0 | 175 |  |
| 645 | 20 | 85605 | 12.4+-5.6 | - | 0/0 | 0 | - | 9 | 0/0/0 | 227 |  |
| 646 | 10 | 49497 | 22.4+-26.6 | - | 0/0 | 0 | - | 0 | 0/0/0 | 12 |  |
| 647 | 114 | 462086 | 21.1+-7.3 | - | 0/0 | 0 | - | 15 | 0/0/0 | 527 |  |
| 648 | 23 | 93175 | 32.7+-26.5 | - | 0/0 | 0 | - | 0 | 0/0/0 | 109 |  |
| 649 | 112 | 459117 | 14.5+-4.8 | - | 0/0 | 0 | - | 1 | 0/0/0 | 435 |  |
| 650 | 41 | 163658 | 17.9+-30.4 | - | 0/0 | 0 | - | 1 | 0/0/0 | 171 |  |
| 651 | 33 | 160001 | 64.2+-18.9 | - | 0/0 | 0 | - | 1 | 0/0/0 | 144 |  |
| 652 | 27 | 116773 | 28.1+-12.1 | - | 0/0 | 0 | - | 0 | 0/0/0 | 67 |  |
| 653 | 78 | 300589 | 19.5+-36.5 | - | 0/0 | 0 | - | 2 | 0/0/0 | 358 |  |
| 654 | 15 | 77064 | 69.1+-103.9 | - | 0/0 | 0 | - | 0 | 0/0/0 | 73 |  |
| 655 | 28 | 127250 | 11.7+-4.2 | - | 0/0 | 0 | - | 0 | 0/0/0 | 243 |  |
| 656 | 34 | 161955 | 34.0+-36.3 | - | 0/0 | 0 | - | 1 | 0/0/0 | 184 |  |
| 657 | 208 | 775926 | 13.9+-7.0 | - | 0/0 | 0 | - | 2 | 1/0/0 | 689 |  |
| 658 | 43 | 608682 | 117.8+-140.8 | - | 0/0 | 0 | - | 2 | 0/0/0 | 813 |  |
| 659 | 11 | 49418 | 35.5+-30.5 | - | 0/0 | 0 | - | 0 | 0/0/0 | 69 |  |
| 660 | 53 | 215785 | 19.8+-16.0 | - | 0/0 | 0 | - | 0 | 0/0/0 | 98 |  |
| 661 | 115 | 459024 | 15.9+-5.1 | - | 0/0 | 0 | - | 4 | 0/0/0 | 472 |  |
| 662 | 23 | 107281 | 64.7+-53.4 | - | 0/0 | 0 | - | 0 | 0/0/0 | 56 |  |
| 663 | 27 | 117879 | 15.1+-12.7 | - | 0/0 | 0 | - | 2 | 0/0/0 | 130 |  |
| 664 | 31 | 159187 | 16.4+-5.2 | - | 0/0 | 0 | - | 0 | 0/0/0 | 117 |  |
| 665 | 9 | 38106 | 21.8+-11.0 | - | 0/0 | 0 | - | 0 | 0/0/0 | 38 |  |
| 666 | 28 | 116917 | 13.0+-5.3 | - | 0/0 | 0 | - | 0 | 0/0/0 | 162 |  |
| 667 | 25 | 113735 | 20.8+-14.4 | - | 0/0 | 0 | - | 2 | 0/0/0 | 139 |  |
| 668 | 43 | 187749 | 22.6+-17.1 | - | 0/0 | 0 | - | 9 | 0/0/0 | 230 |  |
| 669 | 38 | 162573 | 157.0+-617.9 | - | 0/0 | 0 | - | 1 | 0/0/0 | 194 |  |
| 670 | 75 | 290996 | 21.4+-16.1 | - | 0/0 | 0 | - | 5 | 0/0/0 | 310 |  |
| 671 | 71 | 262725 | 21.9+-18.4 | - | 0/0 | 0 | - | 0 | 0/0/0 | 271 |  |
| 672 | 7 | 165514 | 24.1+-5.9 | - | 0/0 | 0 | - | 0 | 0/0/0 | 94 |  |
| 673 | 31 | 144579 | 22.2+-17.1 | - | 0/0 | 0 | - | 1 | 0/0/0 | 114 |  |
| 674 | 8 | 35731 | 39.2+-6.9 | - | 0/0 | 0 | - | 0 | 0/0/0 | 50 |  |
| 675 | 22 | 84206 | 28.3+-20.1 | - | 0/0 | 0 | - | 0 | 0/0/0 | 59 |  |
| 676 | 41 | 203712 | 24.9+-24.9 | - | 0/0 | 0 | - | 13 | 2/0/0 | 216 |  |
| 677 | 18 | 107941 | 38.2+-57.1 | - | 0/0 | 0 | - | 2 | 0/0/0 | 96 |  |
| 678 | 29 | 163697 | 56.9+-66.4 | - | 0/0 | 0 | - | 0 | 0/0/0 | 171 |  |
| 679 | 67 | 290812 | 19.3+-21.7 | - | 0/0 | 0 | - | 2 | 0/0/0 | 198 |  |
| 680 | 13 | 62059 | 21.3+-20.1 | - | 0/0 | 0 | - | 0 | 0/0/0 | 75 |  |
| 681 | 23 | 132723 | 23.6+-23.8 | - | 0/0 | 0 | - | 3 | 0/0/0 | 128 |  |
| 682 | 24 | 115013 | 47.2+-67.1 | - | 0/0 | 0 | - | 1 | 0/0/0 | 169 |  |
| 683 | 13 | 68617 | 23.9+-25.8 | - | 0/0 | 0 | - | 2 | 0/0/0 | 27 |  |
| 684 | 97 | 312098 | 10.6+-4.8 | - | 0/0 | 0 | - | 0 | 0/0/0 | 363 |  |
| 685 | 54 | 250762 | 19.6+-20.3 | - | 0/0 | 0 | - | 3 | 0/0/0 | 270 |  |
| 686 | 59 | 230391 | 11.0+-1.7 | - | 0/0 | 0 | - | 0 | 0/0/0 | 200 |  |
| 687 | 36 | 178969 | 21.7+-21.7 | - | 0/0 | 0 | - | 1 | 1/0/0 | 207 |  |
| 688 | 183 | 647245 | 10.1+-3.6 | - | 0/0 | 0 | - | 5 | 0/0/0 | 690 |  |
| 689 | 38 | 176557 | 26.1+-37.7 | - | 0/0 | 0 | - | 6 | 0/0/0 | 182 |  |
| 690 | 38 | 182871 | 21.6+-16.9 | - | 0/0 | 0 | - | 0 | 0/0/0 | 193 |  |
| 691 | 29 | 148128 | 107.0+-79.2 | - | 0/0 | 0 | - | 0 | 0/0/0 | 122 |  |
| 692 | 40 | 194427 | 18.5+-10.3 | - | 0/0 | 0 | - | 1 | 0/0/0 | 70 |  |
| 693 | 18 | 95590 | 19.5+-13.4 | - | 0/0 | 0 | - | 1 | 0/0/0 | 92 |  |
| 694 | 50 | 243651 | 26.5+-22.1 | - | 0/0 | 0 | - | 5 | 0/0/0 | 279 |  |
| 695 | 22 | 122325 | 60.7+-47.1 | - | 0/0 | 0 | - | 0 | 0/0/0 | 128 |  |
| 696 | 55 | 257637 | 19.2+-11.9 | - | 0/0 | 0 | - | 7 | 0/0/0 | 331 |  |
| 697 | 11 | 51784 | 52.3+-85.4 | - | 0/0 | 0 | - | 0 | 0/0/0 | 81 |  |
| 698 | 36 | 178768 | 18.0+-15.9 | - | 0/0 | 0 | - | 5 | 0/0/0 | 222 |  |
| 699 | 36 | 191525 | 34.3+-33.5 | Thermofilum | 1/1 | 1.0 | 0 | 4 | 0/0/0 | 245 |  |
| 700 | 10 | 248405 | 31.8+-3.6 | - | 0/0 | 0 | - | 0 | 0/0/0 | 348 |  |
| 701 | 29 | 150539 | 40.5+-51.6 | - | 0/0 | 0 | - | 0 | 0/0/0 | 133 |  |
| 702 | 13 | 85212 | 54.6+-93.1 | - | 0/0 | 0 | - | 0 | 0/0/0 | 120 |  |
| 703 | 24 | 107019 | 15.7+-19.6 | - | 0/0 | 0 | - | 0 | 0/0/0 | 54 |  |
| 704 | 2 | 11564 | 47.4+-1.3 | - | 0/0 | 0 | - | 0 | 0/0/0 | 18 |  |
| 705 | 51 | 226941 | 11.7+-2.9 | - | 0/0 | 0 | - | 1 | 0/0/0 | 234 |  |
| 706 | 140 | 580250 | 23.9+-24.7 | - | 0/0 | 0 | - | 18 | 0/0/0 | 700 |  |
| 707 | 32 | 170764 | 23.7+-12.8 | - | 0/0 | 0 | - | 1 | 0/0/0 | 155 |  |
| 708 | 31 | 182214 | 44.7+-37.4 | - | 0/0 | 0 | - | 0 | 0/0/0 | 184 |  |
| 709 | 16 | 93408 | 63.9+-49.1 | - | 0/0 | 0 | - | 0 | 0/0/0 | 49 |  |
| 710 | 14 | 70000 | 21.0+-13.6 | - | 0/0 | 0 | - | 0 | 0/0/0 | 54 |  |
| 711 | 17 | 99197 | 14.5+-4.1 | - | 0/0 | 0 | - | 0 | 0/0/0 | 32 |  |
| 712 | 1 | 8323 | 22.5+-0.0 | - | 0/0 | 0 | - | 0 | 0/0/0 | 7 |  |
| 713 | 50 | 238399 | 25.8+-7.7 | - | 0/0 | 0 | - | 3 | 0/0/0 | 216 |  |
| 714 | 30 | 163097 | 12.1+-3.8 | - | 0/0 | 0 | - | 2 | 0/0/0 | 192 |  |
| 715 | 2 | 14520 | 40.4+-13.1 | - | 0/0 | 0 | - | 0 | 0/0/0 | 1 |  |
| 716 | 163 | 663957 | 11.2+-6.2 | - | 0/0 | 0 | - | 5 | 0/0/0 | 767 |  |
| 717 | 14 | 63342 | 14.9+-3.4 | - | 0/0 | 0 | - | 0 | 0/0/0 | 158 |  |
| 718 | 3 | 12810 | 40.6+-20.8 | - | 0/0 | 0 | - | 0 | 0/0/0 | 16 |  |
| 719 | 15 | 84915 | 18.1+-9.5 | - | 0/0 | 0 | - | 0 | 0/0/0 | 117 |  |
| 720 | 45 | 211445 | 36.5+-38.6 | - | 0/0 | 0 | - | 0 | 0/0/0 | 241 |  |
| 721 | 1 | 8566 | 11.0+-0.0 | - | 0/0 | 0 | - | 0 | 0/0/0 | 11 |  |
| 722 | 49 | 270439 | 42.3+-48.0 | - | 0/0 | 0 | - | 9 | 0/0/0 | 286 |  |
| 723 | 52 | 231344 | 17.0+-24.3 | - | 0/0 | 0 | - | 0 | 0/0/0 | 234 |  |
| 724 | 49 | 220598 | 12.4+-5.4 | - | 0/0 | 0 | - | 0 | 0/0/0 | 239 |  |
| 725 | 15 | 91932 | 17.0+-10.3 | - | 0/0 | 0 | - | 4 | 0/0/0 | 67 |  |
| 726 | 39 | 132933 | 10.7+-8.0 | - | 0/0 | 0 | - | 1 | 0/0/0 | 142 |  |
| 727 | 60 | 302004 | 50.7+-195.3 | - | 0/0 | 0 | - | 4 | 0/0/0 | 319 |  |
| 728 | 1 | 8825 | 12.2+-0.0 | - | 0/0 | 0 | - | 0 | 0/0/0 | 15 |  |
| 729 | 34 | 182046 | 58.0+-46.6 | - | 0/0 | 0 | - | 1 | 0/0/0 | 163 |  |
| 730 | 9 | 48918 | 26.3+-20.2 | - | 0/0 | 0 | - | 0 | 0/0/0 | 77 |  |
| 731 | 46 | 219760 | 23.7+-11.7 | - | 0/0 | 0 | - | 2 | 0/0/0 | 254 |  |
| 732 | 27 | 148920 | 23.1+-15.8 | - | 0/0 | 0 | - | 3 | 1/0/0 | 202 |  |
| 733 | 13 | 90856 | 18.0+-13.5 | - | 0/0 | 0 | - | 2 | 0/0/0 | 103 |  |
| 734 | 54 | 271198 | 35.1+-43.8 | - | 0/0 | 0 | - | 1 | 0/0/0 | 320 |  |
| 735 | 151 | 644750 | 16.2+-8.7 | - | 0/0 | 0 | - | 19 | 0/0/0 | 720 |  |
| 736 | 30 | 180720 | 38.1+-33.5 | - | 0/0 | 0 | - | 1 | 0/0/0 | 161 |  |
| 737 | 18 | 115559 | 63.0+-66.1 | - | 0/0 | 0 | - | 8 | 0/0/0 | 134 |  |
| 738 | 28 | 167875 | 22.7+-33.5 | - | 0/0 | 0 | - | 2 | 0/0/0 | 122 |  |
| 739 | 30 | 139048 | 14.0+-4.9 | - | 0/0 | 0 | - | 0 | 0/0/0 | 159 |  |
| 740 | 41 | 183334 | 18.6+-8.8 | - | 0/0 | 0 | - | 0 | 0/0/0 | 110 |  |
| 741 | 73 | 348498 | 22.0+-26.7 | - | 0/0 | 0 | - | 4 | 0/0/0 | 347 |  |
| 742 | 26 | 161896 | 65.4+-66.3 | - | 0/0 | 0 | - | 3 | 0/0/0 | 174 |  |
| 743 | 19 | 274085 | 56.2+-21.3 | - | 0/0 | 0 | - | 1 | 0/0/0 | 231 |  |
| 744 | 1 | 9238 | 34.0+-0.0 | - | 0/0 | 0 | - | 0 | 0/0/0 | 10 |  |
| 745 | 111 | 530350 | 34.8+-42.9 | - | 0/0 | 0 | - | 2 | 0/0/0 | 566 |  |
| 746 | 55 | 236342 | 16.8+-13.9 | - | 0/0 | 0 | - | 1 | 0/0/0 | 487 |  |
| 747 | 41 | 205444 | 30.4+-44.4 | - | 0/0 | 0 | - | 1 | 0/0/0 | 232 |  |
| 748 | 6 | 37132 | 45.0+-63.4 | - | 0/0 | 0 | - | 0 | 0/1/0 | 29 |  |
| 749 | 33 | 213241 | 19.3+-8.0 | - | 0/0 | 0 | - | 6 | 0/0/0 | 207 |  |
| 750 | 2 | 18318 | 10.6+-1.8 | - | 0/0 | 0 | - | 0 | 0/0/0 | 21 |  |
| 751 | 22 | 151473 | 32.2+-25.0 | - | 0/0 | 0 | - | 0 | 0/0/0 | 94 |  |
| 752 | 76 | 319688 | 11.1+-5.5 | - | 0/0 | 0 | - | 4 | 0/0/0 | 308 |  |
| 753 | 51 | 263172 | 28.1+-31.6 | - | 0/0 | 0 | - | 1 | 0/0/0 | 313 |  |
| 754 | 15 | 93099 | 168.4+-410.8 | - | 0/0 | 0 | - | 0 | 0/0/0 | 121 |  |
| 755 | 29 | 156078 | 29.9+-21.6 | - | 0/0 | 0 | - | 0 | 0/0/0 | 179 |  |
| 756 | 25 | 149378 | 18.3+-10.5 | - | 0/0 | 0 | - | 11 | 0/0/0 | 139 |  |
| 757 | 17 | 103144 | 54.2+-80.1 | - | 0/0 | 0 | - | 0 | 0/0/0 | 119 |  |
| 758 | 20 | 126784 | 18.5+-6.2 | - | 0/0 | 0 | - | 0 | 0/0/0 | 114 |  |
| 759 | 22 | 127600 | 19.7+-21.2 | - | 0/0 | 0 | - | 8 | 0/0/0 | 183 |  |
| 760 | 2 | 19672 | 22.4+-6.4 | - | 0/0 | 0 | - | 0 | 0/0/0 | 28 |  |
| 761 | 39 | 261661 | 39.6+-26.6 | - | 0/0 | 0 | - | 3 | 0/0/0 | 219 |  |
| 762 | 90 | 478888 | 16.5+-4.9 | - | 0/0 | 0 | - | 1 | 0/0/0 | 270 |  |
| 763 | 1 | 10165 | 27.0+-0.0 | - | 0/0 | 0 | - | 0 | 0/0/0 | 11 |  |
| 764 | 37 | 207966 | 83.6+-193.8 | - | 0/0 | 0 | - | 0 | 0/0/0 | 218 |  |
| 765 | 62 | 285435 | 12.8+-3.0 | - | 0/0 | 0 | - | 14 | 0/0/0 | 512 |  |
| 766 | 20 | 124655 | 22.1+-25.4 | - | 0/0 | 0 | - | 0 | 0/0/0 | 212 |  |
| 767 | 82 | 329854 | 16.9+-17.8 | - | 0/0 | 0 | - | 1 | 0/0/0 | 355 |  |
| 768 | 39 | 250765 | 43.7+-59.5 | - | 0/0 | 0 | - | 3 | 1/0/0 | 251 |  |
| 769 | 50 | 283411 | 17.9+-9.8 | - | 0/0 | 0 | - | 1 | 0/0/0 | 252 |  |
| 770 | 46 | 264845 | 13.1+-2.0 | - | 0/0 | 0 | - | 2 | 0/0/0 | 261 |  |
| 771 | 113 | 586032 | 31.0+-12.3 | - | 0/0 | 0 | - | 2 | 0/0/0 | 538 |  |
| 772 | 13 | 96400 | 21.7+-12.0 | - | 0/0 | 0 | - | 0 | 0/0/0 | 41 |  |
| 773 | 2 | 16718 | 18.8+-6.6 | - | 0/0 | 0 | - | 0 | 0/0/0 | 18 |  |
| 774 | 27 | 145546 | 33.6+-15.7 | - | 0/0 | 0 | - | 0 | 0/0/0 | 129 |  |
| 775 | 122 | 2350193 | 44.3+-12.8 | - | 0/0 | 0 | - | 37 | 0/0/0 | 2137 |  |
| 776 | 1 | 10747 | 18.0+-0.0 | - | 0/0 | 0 | - | 0 | 0/0/0 | 12 |  |
| 777 | 34 | 200840 | 59.6+-180.6 | - | 0/0 | 0 | - | 0 | 0/0/0 | 204 |  |
| 778 | 33 | 195744 | 37.7+-29.8 | - | 0/0 | 0 | - | 0 | 0/0/0 | 84 |  |
| 779 | 22 | 145347 | 14.4+-3.4 | - | 0/0 | 0 | - | 5 | 0/0/0 | 149 |  |
| 780 | 22 | 178150 | 67.3+-55.3 | - | 0/0 | 0 | - | 0 | 0/0/0 | 108 |  |
| 781 | 57 | 346941 | 36.3+-25.4 | Candidatus Azobacteroides pseudotrichonymphae | 1/1 | 1.0 | 0 | 7 | 0/0/0 | 348 |  |
| 782 | 1 | 98831 | 31.3+-0.0 | - | 0/0 | 0 | - | 0 | 0/0/0 | 113 |  |
| 783 | 15 | 109435 | 34.5+-26.8 | - | 0/0 | 0 | - | 0 | 0/0/0 | 132 |  |
| 784 | 63 | 310210 | 27.2+-26.4 | - | 0/0 | 0 | - | 5 | 0/0/0 | 316 |  |
| 785 | 25 | 171475 | 40.7+-33.7 | - | 0/0 | 0 | - | 0 | 0/0/0 | 89 |  |
| 786 | 33 | 188073 | 45.8+-19.1 | - | 0/0 | 0 | - | 1 | 0/0/0 | 209 |  |
| 787 | 15 | 83401 | 64.3+-41.5 | - | 0/0 | 0 | - | 0 | 0/0/0 | 54 |  |
| 788 | 103 | 480185 | 13.2+-15.8 | - | 0/0 | 0 | - | 4 | 0/0/0 | 448 |  |
| 789 | 11 | 91598 | 25.5+-15.2 | - | 0/0 | 0 | - | 7 | 0/0/0 | 95 |  |
| 790 | 22 | 442475 | 31.3+-27.5 | - | 0/0 | 0 | - | 1 | 0/0/0 | 417 |  |
| 791 | 116 | 589418 | 21.4+-15.6 | - | 0/0 | 0 | - | 0 | 0/0/0 | 644 |  |
| 792 | 58 | 303034 | 30.8+-37.8 | - | 0/0 | 0 | - | 2 | 0/0/0 | 223 |  |
| 793 | 26 | 157194 | 68.5+-19.5 | - | 0/0 | 0 | - | 0 | 0/0/0 | 152 |  |
| 794 | 16 | 138211 | 70.3+-21.7 | - | 0/0 | 0 | - | 5 | 0/0/0 | 109 |  |
| 795 | 112 | 580292 | 20.3+-16.6 | - | 0/0 | 0 | - | 6 | 0/0/0 | 650 |  |
| 796 | 68 | 328763 | 16.1+-9.8 | - | 0/0 | 0 | - | 4 | 0/0/0 | 314 |  |
| 797 | 4 | 25694 | 14.5+-1.1 | - | 0/0 | 0 | - | 0 | 0/0/0 | 37 |  |
| 798 | 92 | 451942 | 13.6+-6.9 | - | 0/0 | 0 | - | 0 | 0/0/0 | 375 |  |
| 799 | 8 | 33101 | 11.8+-4.7 | - | 0/0 | 0 | - | 0 | 0/0/0 | 48 |  |
| 800 | 34 | 178032 | 14.1+-5.5 | - | 0/0 | 0 | - | 0 | 0/0/0 | 88 |  |
| 801 | 7 | 40859 | 84.9+-133.5 | - | 0/0 | 0 | - | 2 | 0/0/0 | 44 |  |
| 802 | 7 | 48368 | 32.6+-30.2 | - | 0/0 | 0 | - | 0 | 0/0/0 | 70 |  |
| 803 | 168 | 778890 | 26.7+-22.6 | - | 0/0 | 0 | - | 2 | 0/0/0 | 808 |  |
| 804 | 72 | 390767 | 25.1+-26.9 | - | 0/0 | 0 | - | 2 | 1/0/0 | 448 |  |
| 805 | 61 | 438612 | 87.6+-108.5 | - | 0/0 | 0 | - | 1 | 0/0/0 | 459 |  |
| 806 | 31 | 221025 | 19.4+-3.1 | - | 0/0 | 0 | - | 5 | 0/0/0 | 199 |  |
| 807 | 2 | 23759 | 20.5+-2.1 | - | 0/0 | 0 | - | 0 | 0/0/0 | 42 |  |
| 808 | 53 | 310654 | 22.5+-20.2 | - | 0/0 | 0 | - | 12 | 0/0/0 | 334 |  |
| 809 | 1 | 12026 | 74.0+-0.0 | - | 0/0 | 0 | - | 0 | 0/0/0 | 13 |  |
| 810 | 7 | 61433 | 33.4+-25.7 | - | 0/0 | 0 | - | 0 | 0/0/0 | 105 |  |
| 811 | 28 | 166373 | 16.6+-7.2 | - | 0/0 | 0 | - | 0 | 0/0/0 | 127 |  |
| 812 | 23 | 178666 | 27.0+-21.6 | - | 0/0 | 0 | - | 9 | 0/0/0 | 167 |  |
| 813 | 215 | 1013011 | 10.7+-1.4 | - | 0/0 | 0 | - | 5 | 2/0/0 | 961 |  |
| 814 | 21 | 92832 | 47.7+-94.7 | - | 0/0 | 0 | - | 0 | 0/0/0 | 98 |  |
| 815 | 144 | 810743 | 13.1+-6.4 | - | 0/0 | 0 | - | 3 | 0/0/0 | 712 |  |
| 816 | 1 | 12277 | 29.3+-0.0 | - | 0/0 | 0 | - | 0 | 0/0/0 | 14 |  |
| 817 | 14 | 108862 | 21.4+-15.1 | - | 0/0 | 0 | - | 1 | 0/0/0 | 85 |  |
| 818 | 46 | 250757 | 54.0+-41.5 | - | 0/0 | 0 | - | 0 | 0/0/0 | 298 |  |
| 819 | 12 | 95631 | 54.9+-41.9 | - | 0/0 | 0 | - | 0 | 0/0/0 | 121 |  |
| 820 | 17 | 120326 | 50.3+-80.9 | - | 0/0 | 0 | - | 2 | 0/0/0 | 157 |  |
| 821 | 16 | 121578 | 18.2+-14.9 | - | 0/0 | 0 | - | 1 | 0/0/0 | 157 |  |
| 822 | 115 | 662298 | 47.0+-60.3 | - | 0/0 | 0 | - | 1 | 0/0/0 | 652 |  |
| 823 | 67 | 419900 | 13.7+-4.8 | - | 0/0 | 0 | - | 8 | 2/0/0 | 389 |  |
| 824 | 18 | 86622 | 12.9+-2.8 | - | 0/0 | 0 | - | 0 | 0/0/0 | 239 |  |
| 825 | 137 | 670730 | 24.0+-38.6 | - | 0/0 | 0 | - | 9 | 0/0/0 | 634 |  |
| 826 | 34 | 202528 | 28.9+-30.7 | - | 0/0 | 0 | - | 0 | 0/0/0 | 208 |  |
| 827 | 23 | 149120 | 60.6+-114.9 | - | 0/0 | 0 | - | 0 | 0/0/0 | 240 |  |
| 828 | 34 | 257257 | 20.1+-29.5 | - | 0/0 | 0 | - | 3 | 0/0/0 | 369 |  |
| 829 | 5 | 55173 | 50.9+-19.3 | - | 0/0 | 0 | - | 23 | 0/0/0 | 98 |  |
| 830 | 10 | 87982 | 37.9+-64.8 | - | 0/0 | 0 | - | 3 | 0/0/0 | 63 |  |
| 831 | 3 | 27620 | 28.6+-2.1 | - | 0/0 | 0 | - | 0 | 0/0/0 | 26 |  |
| 832 | 3 | 20238 | 910.0+-1023.2 | - | 0/0 | 0 | - | 0 | 0/0/0 | 19 |  |
| 833 | 166 | 916113 | 15.6+-10.9 | - | 0/0 | 0 | - | 10 | 0/0/0 | 896 |  |
| 834 | 49 | 281620 | 14.1+-3.1 | - | 0/0 | 0 | - | 1 | 0/0/0 | 245 |  |
| 835 | 7 | 61798 | 34.6+-21.6 | - | 0/0 | 0 | - | 0 | 0/0/0 | 71 |  |
| 836 | 17 | 140556 | 15.4+-4.6 | - | 0/0 | 0 | - | 1 | 0/0/0 | 157 |  |
| 837 | 5 | 37411 | 37.3+-24.1 | - | 0/0 | 0 | - | 0 | 0/0/0 | 14 |  |
| 838 | 6 | 37536 | 20.2+-9.3 | - | 0/0 | 0 | - | 0 | 0/0/0 | 41 |  |
| 839 | 46 | 309889 | 18.3+-22.0 | - | 0/0 | 0 | - | 8 | 0/0/0 | 344 |  |
| 840 | 9 | 90453 | 68.4+-95.5 | - | 0/0 | 0 | - | 0 | 0/0/0 | 41 |  |
| 841 | 40 | 236892 | 22.1+-12.2 | - | 0/0 | 0 | - | 0 | 0/0/0 | 99 |  |
| 842 | 31 | 205317 | 15.5+-4.4 | - | 0/0 | 0 | - | 0 | 0/0/0 | 53 |  |
| 843 | 87 | 397431 | 12.5+-3.6 | - | 0/0 | 0 | - | 2 | 0/0/0 | 430 |  |
| 844 | 40 | 257351 | 12.7+-2.2 | - | 0/0 | 0 | - | 0 | 0/0/0 | 324 |  |
| 845 | 18 | 125231 | 26.4+-35.8 | - | 0/0 | 0 | - | 0 | 0/0/0 | 194 |  |
| 846 | 40 | 261326 | 14.8+-8.6 | - | 0/0 | 0 | - | 2 | 0/0/0 | 215 |  |
| 847 | 9 | 43188 | 16.1+-2.2 | - | 0/0 | 0 | - | 0 | 0/0/0 | 65 |  |
| 848 | 19 | 158916 | 37.4+-31.9 | - | 0/0 | 0 | - | 0 | 0/0/0 | 125 |  |
| 849 | 3 | 29864 | 417.8+-436.0 | - | 0/0 | 0 | - | 0 | 0/0/0 | 39 |  |
| 850 | 1 | 14199 | 18.0+-0.0 | - | 0/0 | 0 | - | 0 | 0/0/0 | 16 |  |
| 851 | 5 | 25717 | 37.6+-19.7 | - | 0/0 | 0 | - | 1 | 0/0/0 | 30 |  |
| 852 | 34 | 214632 | 15.1+-7.0 | - | 0/0 | 0 | - | 0 | 0/0/0 | 114 |  |
| 853 | 126 | 715722 | 24.8+-19.5 | - | 0/0 | 0 | - | 0 | 0/0/0 | 734 |  |
| 854 | 20 | 195575 | 74.9+-75.6 | - | 0/0 | 0 | - | 1 | 0/0/0 | 169 |  |
| 855 | 82 | 468121 | 31.1+-38.1 | - | 0/0 | 0 | - | 2 | 0/0/0 | 474 |  |
| 856 | 38 | 204879 | 45.1+-30.7 | - | 0/0 | 0 | - | 0 | 0/0/0 | 80 |  |
| 857 | 87 | 467000 | 29.0+-61.0 | - | 0/0 | 0 | - | 2 | 0/0/0 | 574 |  |
| 858 | 67 | 1840329 | 22.8+-5.7 | - | 0/0 | 0 | - | 23 | 2/0/0 | 1415 |  |
| 859 | 114 | 682057 | 23.5+-15.5 | - | 0/0 | 0 | - | 9 | 0/0/0 | 570 |  |
| 860 | 14 | 131892 | 25.5+-18.1 | - | 0/0 | 0 | - | 0 | 0/0/0 | 198 |  |
| 861 | 167 | 886537 | 40.4+-47.6 | - | 0/0 | 0 | - | 7 | 1/0/0 | 938 |  |
| 862 | 15 | 140198 | 17.3+-7.6 | - | 0/0 | 0 | - | 23 | 0/0/0 | 177 |  |
| 863 | 44 | 370333 | 32.7+-25.2 | - | 0/0 | 0 | - | 1 | 0/0/0 | 377 |  |
| 864 | 32 | 223629 | 20.5+-11.2 | - | 0/0 | 0 | - | 1 | 0/0/0 | 252 |  |
| 865 | 57 | 384117 | 32.4+-23.2 | - | 0/0 | 0 | - | 5 | 0/0/0 | 355 |  |
| 866 | 5 | 39275 | 13.1+-3.5 | - | 0/0 | 0 | - | 0 | 0/0/0 | 52 |  |
| 867 | 54 | 2045699 | 46.7+-3.3 | - | 0/0 | 0 | - | 42 | 0/0/0 | 1910 |  |
| 868 | 83 | 422441 | 24.0+-18.7 | - | 0/0 | 0 | - | 3 | 1/0/0 | 439 |  |
| 869 | 96 | 468451 | 13.3+-4.5 | - | 0/0 | 0 | - | 2 | 0/0/0 | 549 |  |
| 870 | 15 | 122634 | 36.8+-34.8 | - | 0/0 | 0 | - | 0 | 0/0/0 | 155 |  |
| 871 | 16 | 155265 | 86.7+-95.9 | - | 0/0 | 0 | - | 4 | 0/0/0 | 230 |  |
| 872 | 26 | 208459 | 20.6+-9.8 | - | 0/0 | 0 | - | 0 | 0/0/0 | 243 |  |
| 873 | 54 | 424846 | 61.7+-33.4 | - | 0/0 | 0 | - | 5 | 0/0/0 | 315 |  |
| 874 | 22 | 142624 | 16.5+-5.6 | - | 0/0 | 0 | - | 0 | 0/0/0 | 194 |  |
| 875 | 64 | 537082 | 37.8+-38.6 | - | 0/0 | 0 | - | 16 | 0/0/0 | 528 |  |
| 876 | 22 | 255648 | 69.0+-6.5 | - | 0/0 | 0 | - | 3 | 0/0/0 | 202 |  |
| 877 | 80 | 568478 | 46.1+-88.9 | - | 0/0 | 0 | - | 6 | 0/0/0 | 615 |  |
| 878 | 5 | 63232 | 15.3+-3.8 | - | 0/0 | 0 | - | 49 | 0/0/0 | 105 |  |
| 879 | 72 | 442140 | 22.0+-19.2 | - | 0/0 | 0 | - | 4 | 1/0/0 | 462 |  |
| 880 | 35 | 229799 | 20.3+-18.4 | - | 0/0 | 0 | - | 2 | 0/0/0 | 322 |  |
| 881 | 11 | 87030 | 41.9+-32.5 | - | 0/0 | 0 | - | 0 | 0/0/0 | 45 |  |
| 882 | 28 | 259351 | 37.0+-27.4 | - | 0/0 | 0 | - | 0 | 0/0/0 | 200 |  |
| 883 | 18 | 147441 | 16.0+-6.1 | - | 0/0 | 0 | - | 0 | 0/0/0 | 246 |  |
| 884 | 61 | 433839 | 44.5+-46.4 | - | 0/0 | 0 | - | 2 | 0/0/0 | 432 |  |
| 885 | 36 | 302977 | 58.1+-57.4 | - | 0/0 | 0 | - | 11 | 1/0/0 | 308 |  |
| 886 | 21 | 199185 | 26.0+-19.3 | - | 0/0 | 0 | - | 1 | 0/0/0 | 229 |  |
| 887 | 84 | 441737 | 15.6+-5.4 | - | 0/0 | 0 | - | 0 | 0/0/0 | 189 |  |
| 888 | 51 | 370824 | 38.4+-32.4 | - | 0/0 | 0 | - | 0 | 0/0/0 | 194 |  |
| 889 | 83 | 507729 | 11.7+-2.1 | - | 0/0 | 0 | - | 4 | 0/0/0 | 465 |  |
| 890 | 22 | 189641 | 14.5+-3.4 | - | 0/0 | 0 | - | 0 | 0/0/0 | 337 |  |
| 891 | 35 | 285565 | 21.0+-8.9 | Methanobacteriaceae | 1/1 | 1.0 | 0 | 2 | 0/0/0 | 315 |  |
| 892 | 42 | 309812 | 38.4+-69.7 | - | 0/0 | 0 | - | 2 | 0/0/0 | 371 |  |
| 893 | 45 | 375207 | 51.0+-44.0 | - | 0/0 | 0 | - | 5 | 0/0/0 | 389 |  |
| 894 | 16 | 119731 | 128.9+-39.1 | - | 0/0 | 0 | - | 0 | 0/0/0 | 87 |  |
| 895 | 87 | 603889 | 49.5+-37.9 | - | 0/0 | 0 | - | 4 | 0/0/0 | 656 |  |
| 896 | 74 | 415945 | 20.9+-14.0 | - | 0/0 | 0 | - | 0 | 0/0/0 | 146 |  |
| 897 | 8 | 64804 | 15.5+-5.6 | - | 0/0 | 0 | - | 0 | 0/0/0 | 105 |  |
| 898 | 19 | 150017 | 20.6+-11.8 | - | 0/0 | 0 | - | 0 | 0/0/0 | 177 |  |
| 899 | 15 | 156807 | 39.7+-13.6 | - | 0/0 | 0 | - | 1 | 0/0/0 | 156 |  |
| 900 | 20 | 270839 | 25.0+-13.8 | - | 0/0 | 0 | - | 13 | 0/0/0 | 467 |  |
| 901 | 71 | 454997 | 16.7+-11.7 | - | 0/0 | 0 | - | 4 | 0/0/0 | 513 |  |
| 902 | 6 | 41185 | 19.1+-4.3 | - | 0/0 | 0 | - | 1 | 0/0/0 | 71 |  |
| 903 | 5 | 41926 | 12.7+-1.7 | - | 0/0 | 0 | - | 0 | 0/0/0 | 60 |  |
| 904 | 4 | 46305 | 41.2+-32.4 | - | 0/0 | 0 | - | 0 | 0/0/0 | 63 |  |
| 905 | 22 | 205427 | 17.1+-9.4 | - | 0/0 | 0 | - | 3 | 0/0/0 | 210 |  |
| 906 | 143 | 575466 | 21.2+-16.0 | - | 0/0 | 0 | - | 1 | 0/0/0 | 665 |  |
| 907 | 38 | 244129 | 32.1+-43.2 | - | 0/0 | 0 | - | 1 | 0/0/0 | 291 |  |
| 908 | 3 | 28285 | 145.8+-15.0 | - | 0/0 | 0 | - | 0 | 0/0/0 | 39 |  |
| 909 | 19 | 166222 | 60.3+-36.8 | - | 0/0 | 0 | - | 1 | 0/0/0 | 59 |  |
| 910 | 6 | 43211 | 17.4+-7.1 | - | 0/0 | 0 | - | 0 | 0/0/0 | 9 |  |
| 911 | 46 | 276489 | 17.7+-4.0 | - | 0/0 | 0 | - | 2 | 0/0/0 | 225 |  |
| 912 | 205 | 1315991 | 18.6+-12.2 | Archaea | 1/1 | 1.0 | 0 | 4 | 0/0/0 | 1176 |  |
| 913 | 2 | 28311 | 48.1+-1.9 | - | 0/0 | 0 | - | 0 | 0/0/0 | 48 |  |
| 914 | 4 | 53918 | 18.3+-3.0 | - | 0/0 | 0 | - | 0 | 0/0/0 | 67 |  |
| 915 | 144 | 1000415 | 26.1+-26.7 | - | 0/0 | 0 | - | 7 | 0/0/0 | 1071 |  |
| 916 | 96 | 693385 | 20.1+-8.7 | - | 0/0 | 0 | - | 3 | 0/0/0 | 270 |  |
| 917 | 108 | 720063 | 16.4+-15.0 | - | 0/0 | 0 | - | 10 | 0/0/0 | 731 |  |
| 918 | 106 | 653641 | 18.9+-10.8 | - | 0/0 | 0 | - | 8 | 1/0/0 | 762 |  |
| 919 | 76 | 564166 | 12.3+-1.8 | - | 0/0 | 0 | - | 10 | 0/0/0 | 556 |  |
| 920 | 4 | 45198 | 13.1+-1.5 | - | 0/0 | 0 | - | 0 | 0/0/0 | 73 |  |
| 921 | 27 | 228468 | 38.9+-62.6 | - | 0/0 | 0 | - | 12 | 0/0/0 | 274 |  |
| 922 | 44 | 401323 | 36.4+-38.9 | - | 0/0 | 0 | - | 0 | 0/0/0 | 258 |  |
| 923 | 136 | 829114 | 11.8+-3.0 | - | 0/0 | 0 | - | 4 | 0/0/0 | 695 |  |
| 924 | 26 | 305952 | 68.2+-58.2 | - | 0/0 | 0 | - | 1 | 0/0/0 | 154 |  |
| 925 | 70 | 492035 | 28.6+-29.4 | - | 0/0 | 0 | - | 7 | 0/0/0 | 544 |  |
| 926 | 31 | 315207 | 14.5+-1.6 | - | 0/0 | 0 | - | 9 | 0/0/0 | 297 |  |
| 927 | 2 | 29980 | 27.2+-1.8 | - | 0/0 | 0 | - | 0 | 0/0/0 | 28 |  |
| 928 | 9 | 125509 | 51.0+-13.3 | - | 0/0 | 0 | - | 0 | 0/0/0 | 47 |  |
| 929 | 12 | 122148 | 18.8+-9.4 | - | 0/0 | 0 | - | 0 | 0/0/0 | 145 |  |
| 930 | 80 | 689087 | 28.8+-20.3 | - | 0/0 | 0 | - | 7 | 1/0/0 | 744 |  |
| 931 | 106 | 688866 | 27.4+-20.0 | - | 0/0 | 0 | - | 12 | 1/0/0 | 686 |  |
| 932 | 11 | 157321 | 22.9+-15.6 | - | 0/0 | 0 | - | 2 | 0/0/0 | 148 |  |
| 933 | 44 | 506827 | 14.1+-1.8 | - | 0/0 | 0 | - | 5 | 3/0/0 | 416 |  |
| 934 | 29 | 233986 | 27.3+-18.7 | - | 0/0 | 0 | - | 0 | 0/0/0 | 297 |  |
| 935 | 8 | 90293 | 18.3+-3.4 | - | 0/0 | 0 | - | 0 | 0/0/0 | 15 |  |
| 936 | 2 | 42645 | 17.4+-2.5 | - | 0/0 | 0 | - | 0 | 0/0/0 | 40 |  |
| 937 | 121 | 778588 | 20.3+-10.3 | - | 0/0 | 0 | - | 7 | 0/0/0 | 846 |  |
| 938 | 48 | 330495 | 17.3+-10.2 | - | 0/0 | 0 | - | 0 | 0/0/0 | 225 |  |
| 939 | 2 | 39389 | 33.6+-6.5 | - | 0/0 | 0 | - | 0 | 0/0/0 | 67 |  |
| 940 | 57 | 413037 | 27.3+-32.8 | - | 0/0 | 0 | - | 1 | 0/0/0 | 276 |  |
| 941 | 10 | 124476 | 19.1+-8.1 | - | 0/0 | 0 | - | 1 | 0/0/0 | 123 |  |
| 942 | 1 | 22635 | 43.0+-0.0 | - | 0/0 | 0 | - | 0 | 0/0/0 | 34 |  |
| 943 | 27 | 332502 | 16.6+-6.0 | - | 0/0 | 0 | - | 22 | 0/0/0 | 391 |  |
| 944 | 15 | 151226 | 24.3+-17.4 | - | 0/0 | 0 | - | 0 | 0/0/0 | 46 |  |
| 945 | 27 | 238468 | 39.1+-53.8 | - | 0/0 | 0 | - | 2 | 0/0/0 | 289 |  |
| 946 | 11 | 111559 | 23.2+-7.5 | - | 0/0 | 0 | - | 1 | 0/0/0 | 149 |  |
| 947 | 10 | 102344 | 36.3+-41.7 | - | 0/0 | 0 | - | 0 | 0/0/0 | 124 |  |
| 948 | 157 | 1244501 | 22.1+-17.6 | - | 0/0 | 0 | - | 16 | 0/0/0 | 1290 |  |
| 949 | 1 | 23046 | 22.0+-0.0 | - | 0/0 | 0 | - | 0 | 0/0/0 | 46 |  |
| 950 | 40 | 368569 | 49.1+-75.0 | - | 0/0 | 0 | - | 3 | 0/0/0 | 388 |  |
| 951 | 116 | 2550055 | 49.9+-22.4 | - | 0/0 | 0 | - | 42 | 0/0/0 | 2148 |  |
| 952 | 34 | 349639 | 28.9+-23.4 | - | 0/0 | 0 | - | 0 | 1/0/0 | 341 |  |
| 953 | 2 | 26330 | 12.7+-1.3 | - | 0/0 | 0 | - | 0 | 0/0/0 | 40 |  |
| 954 | 2 | 33863 | 54.3+-5.9 | - | 0/0 | 0 | - | 0 | 0/0/0 | 52 |  |
| 955 | 16 | 141200 | 19.9+-17.3 | - | 0/0 | 0 | - | 1 | 0/0/0 | 116 |  |
| 956 | 7 | 98997 | 96.1+-84.0 | - | 0/0 | 0 | - | 0 | 0/0/0 | 89 |  |
| 957 | 63 | 543047 | 64.9+-52.5 | Euryarchaeota | 1/1 | 1.0 | 0 | 4 | 1/0/0 | 425 |  |
| 958 | 20 | 175260 | 32.5+-40.6 | - | 0/0 | 0 | - | 14 | 0/0/0 | 276 |  |
| 959 | 187 | 1025850 | 21.8+-20.4 | - | 0/0 | 0 | - | 41 | 1/0/1 | 1115 |  |
| 960 | 110 | 1027535 | 53.1+-62.2 | - | 0/0 | 0 | - | 6 | 2/0/0 | 884 |  |
| 961 | 94 | 950440 | 22.5+-27.5 | - | 0/0 | 0 | - | 26 | 0/0/0 | 883 |  |
| 962 | 4 | 40269 | 21.4+-2.6 | - | 0/0 | 0 | - | 0 | 0/0/0 | 36 |  |
| 963 | 1 | 25685 | 17.0+-0.0 | - | 0/0 | 0 | - | 0 | 0/0/0 | 24 |  |
| 964 | 14 | 200983 | 36.5+-7.1 | - | 0/0 | 0 | - | 6 | 0/0/0 | 183 |  |
| 965 | 37 | 375872 | 20.8+-11.7 | - | 0/0 | 0 | - | 0 | 0/0/0 | 102 |  |
| 966 | 65 | 490063 | 34.2+-22.1 | - | 0/0 | 0 | - | 1 | 0/0/0 | 477 |  |
| 967 | 168 | 2000703 | 41.9+-34.5 | - | 0/0 | 0 | - | 77 | 1/0/0 | 1981 |  |
| 968 | 6 | 76172 | 57.5+-46.5 | - | 0/0 | 0 | - | 0 | 0/0/0 | 99 |  |
| 969 | 7 | 301129 | 63.7+-27.6 | - | 0/0 | 0 | - | 8 | 0/0/0 | 395 |  |
| 970 | 13 | 216239 | 61.5+-28.8 | - | 0/0 | 0 | - | 2 | 0/0/0 | 104 |  |
| 971 | 149 | 958152 | 26.7+-21.3 | - | 0/0 | 0 | - | 6 | 0/0/0 | 1094 |  |
| 972 | 6 | 108017 | 25.7+-6.3 | - | 0/0 | 0 | - | 0 | 0/0/0 | 147 |  |
| 973 | 41 | 280020 | 39.1+-35.0 | - | 0/0 | 0 | - | 2 | 0/0/0 | 320 |  |
| 974 | 72 | 630638 | 21.6+-33.0 | - | 0/0 | 0 | - | 10 | 0/0/0 | 568 |  |
| 975 | 14 | 163577 | 40.0+-41.3 | - | 0/0 | 0 | - | 0 | 0/0/0 | 142 |  |
| 976 | 46 | 446690 | 28.9+-16.1 | - | 0/0 | 0 | - | 2 | 0/0/0 | 403 |  |
| 977 | 40 | 329649 | 50.4+-85.9 | - | 0/0 | 0 | - | 6 | 0/0/0 | 383 |  |
| 978 | 4 | 75556 | 55.1+-22.0 | - | 0/0 | 0 | - | 0 | 0/0/0 | 105 |  |
| 979 | 35 | 273347 | 16.7+-7.8 | - | 0/0 | 0 | - | 1 | 0/0/0 | 408 |  |
| 980 | 8 | 91157 | 20.2+-5.4 | - | 0/0 | 0 | - | 0 | 0/0/0 | 123 |  |
| 981 | 21 | 219727 | 36.4+-16.0 | Sulfolobaceae | 1/1 | 1.0 | 0 | 7 | 0/0/0 | 238 |  |
| 982 | 49 | 556765 | 64.8+-26.6 | - | 0/0 | 0 | - | 1 | 0/0/0 | 333 |  |
| 983 | 72 | 689984 | 33.9+-37.5 | - | 0/0 | 0 | - | 1 | 0/0/0 | 725 |  |
| 984 | 9 | 131577 | 54.7+-47.2 | - | 0/0 | 0 | - | 1 | 0/0/0 | 193 |  |
| 985 | 76 | 626738 | 21.1+-29.9 | - | 0/0 | 0 | - | 20 | 0/0/0 | 616 |  |
| 986 | 12 | 148006 | 24.9+-8.5 | - | 0/0 | 0 | - | 3 | 0/0/0 | 149 |  |
| 987 | 46 | 514259 | 22.2+-15.1 | Thermoprotei | 1/1 | 1.0 | 0 | 5 | 0/0/0 | 535 |  |
| 988 | 162 | 1364608 | 38.4+-42.6 | - | 0/0 | 0 | - | 5 | 0/0/0 | 1341 |  |
| 989 | 28 | 487167 | 73.9+-12.8 | - | 0/0 | 0 | - | 5 | 0/0/0 | 395 |  |
| 990 | 3 | 72390 | 25.8+-5.8 | - | 0/0 | 0 | - | 0 | 0/0/0 | 117 |  |
| 991 | 72 | 655638 | 82.1+-91.7 | - | 0/0 | 0 | - | 3 | 0/0/0 | 704 |  |
| 992 | 1 | 32998 | 45.0+-0.0 | - | 0/0 | 0 | - | 0 | 0/0/0 | 50 |  |
| 993 | 108 | 827581 | 18.7+-25.9 | - | 0/0 | 0 | - | 13 | 0/0/0 | 849 |  |
| 994 | 135 | 1041722 | 21.7+-21.8 | - | 0/0 | 0 | - | 7 | 1/0/0 | 915 |  |
| 995 | 90 | 754686 | 16.9+-11.7 | - | 0/0 | 0 | - | 19 | 1/0/0 | 803 |  |
| 996 | 1 | 34376 | 55.9+-0.0 | - | 0/0 | 0 | - | 0 | 0/0/0 | 41 |  |
| 997 | 1 | 35058 | 77.7+-0.0 | - | 0/0 | 0 | - | 0 | 0/0/0 | 54 |  |
| 998 | 12 | 154194 | 42.9+-35.1 | - | 0/0 | 0 | - | 0 | 0/0/0 | 233 |  |
| 999 | 58 | 544941 | 30.9+-22.8 | - | 0/0 | 0 | - | 4 | 0/0/0 | 550 |  |
| 1000 | 18 | 188341 | 15.0+-7.2 | - | 0/0 | 0 | - | 2 | 0/0/0 | 216 |  |
| 1001 | 77 | 889972 | 147.3+-19.4 | - | 0/0 | 0 | - | 4 | 1/0/0 | 733 |  |
| 1002 | 129 | 1555640 | 63.2+-39.1 | - | 0/0 | 0 | - | 23 | 2/0/0 | 1276 |  |
| 1003 | 2 | 40674 | 22.9+-4.4 | - | 0/0 | 0 | - | 0 | 0/0/0 | 55 |  |
| 1004 | 40 | 399998 | 52.5+-57.2 | - | 0/0 | 0 | - | 0 | 0/0/0 | 211 |  |
| 1005 | 170 | 1701044 | 25.6+-21.3 | - | 0/0 | 0 | - | 17 | 1/0/0 | 1659 |  |
| 1006 | 1 | 37437 | 27.2+-0.0 | - | 0/0 | 0 | - | 0 | 0/0/0 | 48 |  |
| 1007 | 160 | 1293353 | 17.1+-4.5 | - | 0/0 | 0 | - | 17 | 0/0/0 | 1211 |  |
| 1008 | 104 | 1119442 | 45.1+-65.2 | - | 0/0 | 0 | - | 7 | 2/0/0 | 1132 |  |
| 1009 | 11 | 103321 | 18.3+-8.3 | - | 0/0 | 0 | - | 2 | 0/0/0 | 163 |  |
| 1010 | 2 | 186858 | 27.0+-1.4 | - | 0/0 | 0 | - | 31 | 0/0/0 | 229 |  |
| 1011 | 158 | 1335137 | 26.1+-16.5 | - | 0/0 | 0 | - | 11 | 0/0/0 | 1302 |  |
| 1012 | 16 | 204320 | 52.1+-32.2 | - | 0/0 | 0 | - | 2 | 0/0/0 | 295 |  |
| 1013 | 123 | 1134445 | 85.5+-163.4 | - | 0/0 | 0 | - | 18 | 1/0/0 | 1095 |  |
| 1014 | 1 | 38825 | 48.0+-0.0 | - | 0/0 | 0 | - | 0 | 0/0/0 | 71 |  |
| 1015 | 1 | 38897 | 15.0+-0.0 | - | 0/0 | 0 | - | 0 | 0/0/0 | 64 |  |
| 1016 | 1 | 39059 | 21.0+-0.0 | - | 0/0 | 0 | - | 0 | 0/0/0 | 44 |  |
| 1017 | 58 | 822809 | 56.2+-60.5 | - | 0/0 | 0 | - | 7 | 0/0/0 | 721 |  |
| 1018 | 2 | 78365 | 72.9+-7.1 | - | 0/0 | 0 | - | 0 | 0/0/0 | 137 |  |
| 1019 | 2 | 41927 | 50.0+-11.7 | - | 0/0 | 0 | - | 0 | 0/0/0 | 63 |  |
| 1020 | 89 | 929095 | 57.0+-42.7 | - | 0/0 | 0 | - | 3 | 0/0/0 | 681 |  |
| 1021 | 39 | 459247 | 39.0+-27.6 | - | 0/0 | 0 | - | 0 | 0/0/0 | 239 |  |
| 1022 | 2 | 78829 | 23.4+-4.0 | - | 0/0 | 0 | - | 0 | 0/0/0 | 119 |  |
| 1023 | 2 | 77742 | 49.5+-28.3 | - | 0/0 | 0 | - | 1 | 0/0/0 | 122 |  |
| 1024 | 2 | 45116 | 38.0+-3.3 | - | 0/0 | 0 | - | 0 | 0/0/0 | 56 |  |
| 1025 | 326 | 2618889 | 19.7+-9.4 | - | 0/0 | 0 | - | 19 | 0/0/0 | 2554 |  |
| 1026 | 14 | 189451 | 18.2+-3.6 | - | 0/0 | 0 | - | 11 | 0/0/0 | 309 |  |
| 1027 | 38 | 464977 | 13.6+-1.9 | - | 0/0 | 0 | - | 7 | 0/0/0 | 454 |  |
| 1028 | 122 | 874401 | 25.8+-25.0 | - | 0/0 | 0 | - | 6 | 1/0/0 | 887 |  |
| 1029 | 149 | 970089 | 28.7+-23.8 | - | 0/0 | 0 | - | 4 | 2/0/0 | 1083 |  |
| 1030 | 28 | 312458 | 22.0+-6.5 | - | 0/0 | 0 | - | 21 | 0/0/0 | 426 |  |
| 1031 | 108 | 901352 | 28.7+-18.1 | - | 0/0 | 0 | - | 8 | 0/0/0 | 988 |  |
| 1032 | 50 | 697256 | 46.8+-34.1 | - | 0/0 | 0 | - | 10 | 1/0/0 | 541 |  |
| 1033 | 5 | 69518 | 19.7+-14.6 | - | 0/0 | 0 | - | 0 | 0/0/0 | 90 |  |
| 1034 | 48 | 413245 | 24.2+-16.7 | - | 0/0 | 0 | - | 0 | 0/0/0 | 414 |  |
| 1035 | 1 | 48183 | 23.0+-0.0 | - | 0/0 | 0 | - | 0 | 0/0/0 | 45 |  |
| 1036 | 1 | 48331 | 61.0+-0.0 | - | 0/0 | 0 | - | 0 | 0/0/0 | 76 |  |
| 1037 | 38 | 373996 | 21.7+-14.5 | - | 0/0 | 0 | - | 4 | 0/0/0 | 356 |  |
| 1038 | 29 | 219378 | 22.7+-13.5 | - | 0/0 | 0 | - | 17 | 0/0/0 | 440 |  |
| 1039 | 2 | 54297 | 19.2+-3.8 | - | 0/0 | 0 | - | 0 | 0/0/0 | 83 |  |
| 1040 | 353 | 3344087 | 16.9+-8.3 | - | 6/16 | - | - | 28 | 0/0/0 | 2787 |  |
| 1041 | 3 | 6000 | 21.7+-17.0 | - | 0/0 | 0 | - | 0 | 0/0/0 | 9 |  |
| 1042 | 4 | 8012 | 13.0+-6.8 | - | 0/0 | 0 | - | 0 | 0/0/0 | 14 |  |
| 1043 | 4 | 8019 | 20.5+-21.7 | - | 0/0 | 0 | - | 0 | 0/0/0 | 9 |  |
| 1044 | 3 | 6014 | 11.3+-2.1 | - | 0/0 | 0 | - | 0 | 0/0/0 | 6 |  |
| 1045 | 10 | 20072 | 11.9+-2.7 | - | 0/0 | 0 | - | 1 | 0/0/0 | 32 |  |
| 1046 | 6 | 12091 | 15.0+-12.8 | - | 0/0 | 0 | - | 0 | 0/0/0 | 17 |  |
| 1047 | 5 | 10093 | 11.4+-4.0 | - | 0/0 | 0 | - | 0 | 0/0/0 | 14 |  |
| 1048 | 24 | 210865 | 73.7+-57.9 | - | 0/0 | 0 | - | 9 | 0/0/0 | 218 |  |
| 1049 | 9 | 18164 | 13.4+-7.0 | - | 0/0 | 0 | - | 0 | 0/0/0 | 23 |  |
| 1050 | 2 | 4048 | 77.1+-77.8 | - | 0/0 | 0 | - | 0 | 0/0/0 | 4 |  |
| 1051 | 9 | 18192 | 11.5+-4.3 | - | 0/0 | 0 | - | 0 | 0/0/0 | 20 |  |
| 1052 | 9 | 18224 | 16.0+-10.5 | - | 0/0 | 0 | - | 0 | 0/0/0 | 24 |  |
| 1053 | 11 | 22261 | 10.0+-1.7 | - | 0/0 | 0 | - | 0 | 0/0/0 | 27 |  |
| 1054 | 9 | 18199 | 60.4+-113.2 | - | 0/0 | 0 | - | 0 | 0/0/0 | 30 |  |
| 1055 | 14 | 28336 | 20.4+-28.8 | - | 0/0 | 0 | - | 0 | 0/0/0 | 39 |  |
| 1056 | 4 | 8120 | 15.5+-11.4 | - | 0/0 | 0 | - | 0 | 0/0/0 | 7 |  |
| 1057 | 11 | 22315 | 16.4+-12.0 | - | 0/0 | 0 | - | 0 | 0/0/0 | 23 |  |
| 1058 | 8 | 16280 | 17.4+-10.1 | - | 0/0 | 0 | - | 0 | 0/0/0 | 22 |  |
| 1059 | 7 | 14257 | 20.6+-18.7 | - | 0/0 | 0 | - | 0 | 0/0/0 | 21 |  |
| 1060 | 7 | 14228 | 10.0+-2.4 | - | 0/0 | 0 | - | 0 | 0/0/0 | 20 |  |
| 1061 | 11 | 22487 | 22.8+-18.1 | - | 0/0 | 0 | - | 0 | 0/0/0 | 37 |  |
| 1062 | 4 | 8186 | 26.1+-32.1 | - | 0/0 | 0 | - | 0 | 0/0/0 | 14 |  |
| 1063 | 10 | 20412 | 16.0+-8.4 | - | 0/0 | 0 | - | 0 | 0/0/0 | 13 |  |
| 1064 | 12 | 24561 | 10.0+-1.1 | - | 0/0 | 0 | - | 1 | 0/0/0 | 40 |  |
| 1065 | 49 | 431775 | 29.0+-22.6 | - | 0/0 | 0 | - | 13 | 1/0/0 | 433 |  |
| 1066 | 7 | 14303 | 12.5+-6.3 | - | 0/0 | 0 | - | 0 | 0/0/0 | 14 |  |
| 1067 | 13 | 26629 | 14.3+-11.1 | - | 0/0 | 0 | - | 6 | 0/0/0 | 38 |  |
| 1068 | 10 | 20547 | 11.4+-3.4 | - | 0/0 | 0 | - | 0 | 0/0/0 | 20 |  |
| 1069 | 13 | 26624 | 65.0+-144.4 | - | 0/0 | 0 | - | 0 | 0/0/0 | 30 |  |
| 1070 | 8 | 16429 | 31.4+-61.9 | - | 0/0 | 0 | - | 0 | 0/0/0 | 18 |  |
| 1071 | 9 | 18546 | 24.0+-20.4 | - | 0/0 | 0 | - | 0 | 0/0/0 | 16 |  |
| 1072 | 11 | 22676 | 20.8+-25.1 | - | 0/0 | 0 | - | 0 | 1/0/0 | 32 |  |
| 1073 | 9 | 18611 | 15.7+-9.4 | - | 0/0 | 0 | - | 0 | 0/0/0 | 25 |  |
| 1074 | 11 | 22673 | 18.5+-19.6 | - | 0/0 | 0 | - | 2 | 0/0/0 | 30 |  |
| 1075 | 12 | 24811 | 18.5+-19.7 | - | 0/0 | 0 | - | 4 | 0/0/0 | 30 |  |
| 1076 | 11 | 22721 | 96.0+-254.6 | - | 0/0 | 0 | - | 0 | 0/0/0 | 23 |  |
| 1077 | 37 | 527704 | 15.1+-4.4 | - | 0/0 | 0 | - | 11 | 2/0/0 | 504 |  |
| 1078 | 17 | 35096 | 15.5+-12.7 | - | 0/0 | 0 | - | 0 | 0/0/0 | 39 |  |
| 1079 | 11 | 22867 | 13.4+-12.0 | - | 0/0 | 0 | - | 0 | 0/0/0 | 29 |  |
| 1080 | 15 | 31137 | 26.0+-17.3 | - | 0/0 | 0 | - | 1 | 1/0/0 | 34 |  |
| 1081 | 7 | 14610 | 11.4+-3.0 | - | 0/0 | 0 | - | 0 | 0/0/0 | 18 |  |
| 1082 | 11 | 22944 | 14.0+-8.9 | - | 0/0 | 0 | - | 0 | 0/0/0 | 28 |  |
| 1083 | 2 | 4167 | 13.1+-7.1 | - | 0/0 | 0 | - | 0 | 0/0/0 | 3 |  |
| 1084 | 16 | 33370 | 15.2+-17.4 | - | 0/0 | 0 | - | 0 | 1/0/0 | 44 |  |
| 1085 | 17 | 35328 | 23.2+-32.3 | - | 0/0 | 0 | - | 1 | 0/0/0 | 49 |  |
| 1086 | 13 | 27027 | 14.9+-13.1 | - | 0/0 | 0 | - | 0 | 0/0/0 | 35 |  |
| 1087 | 19 | 39622 | 23.9+-47.0 | - | 0/0 | 0 | - | 0 | 0/0/0 | 48 |  |
| 1088 | 14 | 29290 | 12.8+-8.6 | - | 0/0 | 0 | - | 2 | 0/0/0 | 35 |  |
| 1089 | 13 | 27081 | 22.9+-21.2 | - | 0/0 | 0 | - | 1 | 0/0/0 | 30 |  |
| 1090 | 19 | 39440 | 34.9+-37.3 | - | 0/0 | 0 | - | 2 | 0/0/0 | 48 |  |
| 1091 | 14 | 29443 | 15.1+-6.2 | - | 0/0 | 0 | - | 0 | 0/0/0 | 39 |  |
| 1092 | 4 | 8422 | 39.4+-37.2 | - | 0/0 | 0 | - | 0 | 0/0/0 | 4 |  |
| 1093 | 18 | 37729 | 15.0+-11.7 | - | 0/0 | 0 | - | 0 | 0/0/0 | 44 |  |
| 1094 | 56 | 1161048 | 35.9+-4.5 | - | 0/0 | 0 | - | 25 | 1/0/0 | 1067 |  |
| 1095 | 10 | 21012 | 32.8+-59.4 | - | 0/0 | 0 | - | 0 | 0/0/0 | 22 |  |
| 1096 | 13 | 27580 | 14.3+-7.4 | - | 0/0 | 0 | - | 0 | 0/0/0 | 42 |  |
| 1097 | 19 | 39949 | 28.1+-19.2 | - | 0/0 | 0 | - | 1 | 0/0/0 | 49 |  |
| 1098 | 3 | 6224 | 62.9+-93.9 | - | 0/0 | 0 | - | 0 | 0/0/0 | 6 |  |
| 1099 | 3 | 6293 | 19.0+-16.6 | - | 0/0 | 0 | - | 0 | 0/0/0 | 3 |  |
| 1100 | 13 | 27461 | 15.4+-11.3 | - | 0/0 | 0 | - | 0 | 0/0/0 | 35 |  |
| 1101 | 18 | 37850 | 11.5+-4.6 | - | 0/0 | 0 | - | 1 | 0/0/0 | 46 |  |
| 1102 | 9 | 18973 | 12.4+-3.1 | - | 0/0 | 0 | - | 0 | 0/0/0 | 16 |  |
| 1103 | 19 | 40042 | 14.1+-11.3 | - | 0/0 | 0 | - | 0 | 0/0/0 | 54 |  |
| 1104 | 19 | 40045 | 13.9+-10.0 | - | 0/0 | 0 | - | 0 | 1/0/0 | 44 |  |
| 1105 | 20 | 42230 | 13.8+-7.9 | - | 0/0 | 0 | - | 0 | 0/0/0 | 43 |  |
| 1106 | 20 | 42967 | 22.7+-20.4 | - | 0/0 | 0 | - | 0 | 0/0/0 | 66 |  |
| 1107 | 21 | 44824 | 10.9+-3.4 | - | 0/0 | 0 | - | 0 | 0/0/0 | 61 |  |
| 1108 | 20 | 42578 | 28.7+-40.6 | - | 0/0 | 0 | - | 0 | 0/0/0 | 43 |  |
| 1109 | 7 | 15054 | 12.6+-4.0 | - | 0/0 | 0 | - | 0 | 0/0/0 | 10 |  |
| 1110 | 20 | 43202 | 27.7+-33.6 | - | 0/0 | 0 | - | 1 | 0/0/0 | 59 |  |
| 1111 | 15 | 31978 | 24.4+-21.7 | - | 0/0 | 0 | - | 0 | 0/0/0 | 29 |  |
| 1112 | 20 | 42869 | 15.5+-15.3 | - | 0/0 | 0 | - | 3 | 0/0/0 | 65 |  |
| 1113 | 23 | 49329 | 14.0+-8.9 | - | 0/0 | 0 | - | 1 | 0/0/0 | 59 |  |
| 1114 | 22 | 47352 | 23.9+-23.0 | - | 0/0 | 0 | - | 3 | 0/1/0 | 61 |  |
| 1115 | 16 | 34563 | 12.8+-7.6 | - | 0/0 | 0 | - | 0 | 0/0/0 | 43 |  |
| 1116 | 16 | 34833 | 16.9+-8.1 | - | 0/0 | 0 | - | 0 | 0/0/0 | 48 |  |
| 1117 | 16 | 34389 | 15.4+-11.4 | - | 0/0 | 0 | - | 0 | 0/0/0 | 44 |  |
| 1118 | 1 | 2221 | 15.0+-0.0 | - | 0/0 | 0 | - | 0 | 0/0/0 | 0 |  |
| 1119 | 78 | 1279258 | 30.0+-24.8 | - | 0/0 | 0 | - | 6 | 0/0/0 | 954 |  |
| 1120 | 19 | 40980 | 12.1+-4.6 | - | 0/0 | 0 | - | 1 | 0/0/0 | 49 |  |
| 1121 | 25 | 53724 | 28.5+-48.5 | - | 0/0 | 0 | - | 5 | 0/0/0 | 79 |  |
| 1122 | 11 | 23648 | 14.3+-6.3 | - | 0/0 | 0 | - | 0 | 0/0/0 | 30 |  |
| 1123 | 7 | 14961 | 18.0+-16.4 | - | 0/0 | 0 | - | 0 | 0/0/0 | 14 |  |
| 1124 | 17 | 36949 | 15.0+-6.5 | - | 0/0 | 0 | - | 0 | 0/0/0 | 49 |  |
| 1125 | 19 | 41712 | 11.4+-3.7 | - | 0/0 | 0 | - | 0 | 0/0/0 | 43 |  |
| 1126 | 18 | 39179 | 23.4+-26.1 | - | 0/0 | 0 | - | 3 | 0/0/0 | 47 |  |
| 1127 | 20 | 43423 | 16.1+-7.1 | - | 0/0 | 0 | - | 0 | 0/0/0 | 57 |  |
| 1128 | 2 | 4468 | 10.5+-2.1 | - | 0/0 | 0 | - | 0 | 0/0/0 | 2 |  |
| 1129 | 14 | 30268 | 16.1+-8.4 | - | 0/0 | 0 | - | 0 | 0/0/0 | 23 |  |
| 1130 | 20 | 43989 | 23.4+-39.3 | - | 0/0 | 0 | - | 0 | 0/0/0 | 57 |  |
| 1131 | 13 | 28497 | 13.6+-7.5 | - | 0/0 | 0 | - | 0 | 0/0/0 | 23 |  |
| 1132 | 19 | 41814 | 151.4+-505.1 | - | 0/0 | 0 | - | 1 | 0/0/0 | 58 |  |
| 1133 | 19 | 41602 | 22.0+-40.0 | - | 0/0 | 0 | - | 6 | 1/0/0 | 55 |  |
| 1134 | 10 | 21783 | 54.8+-136.8 | - | 0/0 | 0 | - | 0 | 0/0/0 | 31 |  |
| 1135 | 13 | 28267 | 29.6+-35.5 | - | 0/0 | 0 | - | 1 | 0/0/0 | 21 |  |
| 1136 | 124 | 1127653 | 33.2+-23.4 | - | 0/0 | 0 | - | 7 | 0/0/0 | 1197 |  |
| 1137 | 19 | 41128 | 21.6+-18.0 | - | 0/0 | 0 | - | 0 | 0/0/0 | 48 |  |
| 1138 | 19 | 41614 | 18.0+-21.2 | - | 0/0 | 0 | - | 0 | 0/0/0 | 21 |  |
| 1139 | 11 | 24485 | 27.1+-41.8 | - | 0/0 | 0 | - | 7 | 0/0/0 | 24 |  |
| 1140 | 73 | 953532 | 14.2+-1.6 | - | 0/0 | 0 | - | 11 | 0/0/0 | 801 |  |
| 1141 | 26 | 57330 | 16.3+-11.3 | - | 0/0 | 0 | - | 3 | 0/0/0 | 73 |  |
| 1142 | 27 | 59286 | 15.7+-12.3 | - | 0/0 | 0 | - | 0 | 0/0/0 | 77 |  |
| 1143 | 18 | 39998 | 12.8+-7.1 | - | 0/0 | 0 | - | 0 | 0/0/0 | 39 |  |
| 1144 | 4 | 8982 | 12.6+-9.1 | - | 0/0 | 0 | - | 0 | 0/0/0 | 4 |  |
| 1145 | 16 | 35749 | 18.3+-15.0 | - | 0/0 | 0 | - | 1 | 0/0/0 | 43 |  |
| 1146 | 15 | 32584 | 11.4+-4.3 | - | 0/0 | 0 | - | 0 | 0/0/0 | 47 |  |
| 1147 | 14 | 31253 | 73.5+-153.5 | - | 0/0 | 0 | - | 0 | 0/0/0 | 35 |  |
| 1148 | 12 | 26504 | 44.2+-76.2 | - | 0/0 | 0 | - | 0 | 0/0/0 | 34 |  |
| 1149 | 10 | 21866 | 41.1+-48.8 | - | 0/0 | 0 | - | 0 | 0/0/0 | 12 |  |
| 1150 | 19 | 42710 | 21.7+-19.3 | - | 0/0 | 0 | - | 0 | 0/0/0 | 54 |  |
| 1151 | 12 | 26253 | 13.6+-5.0 | - | 0/0 | 0 | - | 0 | 0/0/0 | 14 |  |
| 1152 | 19 | 41240 | 17.2+-23.2 | - | 0/0 | 0 | - | 2 | 0/0/0 | 39 |  |
| 1153 | 8 | 17922 | 9.5+-2.8 | - | 0/0 | 0 | - | 0 | 0/0/0 | 22 |  |
| 1154 | 205 | 1857109 | 41.1+-56.3 | - | 0/0 | 0 | - | 8 | 3/0/0 | 1822 |  |
| 1155 | 19 | 42572 | 16.6+-10.8 | - | 0/0 | 0 | - | 1 | 0/0/0 | 50 |  |
| 1156 | 19 | 41861 | 9.6+-2.1 | - | 0/0 | 0 | - | 1 | 0/0/0 | 43 |  |
| 1157 | 23 | 50999 | 11.0+-3.9 | - | 0/0 | 0 | - | 0 | 0/0/0 | 58 |  |
| 1158 | 16 | 35699 | 20.8+-30.6 | - | 0/0 | 0 | - | 0 | 0/0/0 | 40 |  |
| 1159 | 15 | 34380 | 12.5+-5.8 | - | 0/0 | 0 | - | 0 | 0/0/0 | 51 |  |
| 1160 | 22 | 49439 | 26.9+-41.9 | - | 0/0 | 0 | - | 0 | 0/0/0 | 68 |  |
| 1161 | 22 | 50580 | 20.3+-15.1 | - | 0/0 | 0 | - | 0 | 1/0/0 | 65 |  |
| 1162 | 19 | 41627 | 14.6+-14.0 | - | 0/0 | 0 | - | 0 | 0/0/0 | 34 |  |
| 1163 | 20 | 45173 | 17.7+-12.9 | - | 0/0 | 0 | - | 0 | 0/0/0 | 48 |  |
| 1164 | 20 | 45723 | 30.1+-40.5 | - | 0/0 | 0 | - | 0 | 0/0/0 | 42 |  |
| 1165 | 18 | 40974 | 10.6+-1.9 | - | 0/0 | 0 | - | 0 | 0/0/0 | 46 |  |
| 1166 | 19 | 43818 | 14.3+-5.9 | - | 0/0 | 0 | - | 3 | 0/0/0 | 59 |  |
| 1167 | 18 | 40876 | 18.8+-13.6 | - | 0/0 | 0 | - | 0 | 0/0/0 | 50 |  |
| 1168 | 23 | 52681 | 13.0+-6.5 | - | 0/0 | 0 | - | 0 | 0/0/0 | 73 |  |
| 1169 | 16 | 36939 | 41.2+-54.9 | - | 0/0 | 0 | - | 0 | 0/0/0 | 48 |  |
| 1170 | 17 | 38090 | 15.8+-18.5 | - | 0/0 | 0 | - | 0 | 0/0/0 | 41 |  |
| 1171 | 20 | 46014 | 19.3+-14.7 | - | 0/0 | 0 | - | 0 | 0/0/0 | 66 |  |
| 1172 | 16 | 36675 | 20.4+-21.4 | - | 0/0 | 0 | - | 0 | 0/0/0 | 40 |  |
| 1173 | 19 | 43649 | 12.2+-4.2 | - | 0/0 | 0 | - | 0 | 0/0/0 | 65 |  |
| 1174 | 15 | 33997 | 27.5+-60.5 | - | 0/0 | 0 | - | 0 | 0/0/0 | 32 |  |
| 1175 | 26 | 58751 | 13.9+-4.0 | - | 0/0 | 0 | - | 0 | 0/0/0 | 68 |  |
| 1176 | 24 | 56032 | 43.3+-113.1 | Nanoarchaeum equitans | 1/1 | 1.0 | 0 | 3 | 0/0/0 | 80 |  |
| 1177 | 31 | 419125 | 59.0+-84.2 | - | 0/0 | 0 | - | 14 | 0/0/0 | 495 |  |
| 1178 | 21 | 49633 | 15.8+-5.6 | - | 0/0 | 0 | - | 0 | 0/0/0 | 69 |  |
| 1179 | 14 | 32880 | 14.3+-5.5 | - | 0/0 | 0 | - | 0 | 0/0/0 | 25 |  |
| 1180 | 20 | 46453 | 20.8+-22.8 | - | 0/0 | 0 | - | 2 | 0/0/0 | 19 |  |
| 1181 | 23 | 53329 | 12.7+-3.2 | - | 0/0 | 0 | - | 0 | 0/0/0 | 45 |  |
| 1182 | 75 | 959671 | 17.7+-4.0 | - | 0/0 | 0 | - | 4 | 0/0/0 | 975 |  |
| 1183 | 20 | 47892 | 16.8+-6.9 | - | 0/0 | 0 | - | 0 | 0/0/0 | 64 |  |
| 1184 | 23 | 53278 | 28.9+-34.3 | - | 0/0 | 0 | - | 0 | 0/0/0 | 69 |  |
| 1185 | 22 | 52728 | 19.9+-24.8 | - | 0/0 | 0 | - | 0 | 0/0/0 | 75 |  |
| 1186 | 19 | 45204 | 25.5+-27.4 | - | 0/0 | 0 | - | 0 | 0/0/0 | 53 |  |
| 1187 | 10 | 23774 | 27.4+-22.9 | - | 0/0 | 0 | - | 0 | 0/0/0 | 21 |  |
| 1188 | 15 | 35006 | 23.0+-16.1 | - | 0/0 | 0 | - | 5 | 0/0/0 | 41 |  |
| 1189 | 20 | 46061 | 33.4+-31.0 | - | 0/0 | 0 | - | 0 | 0/0/0 | 29 |  |
| 1190 | 16 | 37710 | 14.8+-8.9 | - | 0/0 | 0 | - | 0 | 0/0/0 | 39 |  |
| 1191 | 25 | 59829 | 15.0+-11.1 | - | 0/0 | 0 | - | 8 | 0/0/0 | 78 |  |
| 1192 | 20 | 48617 | 23.8+-16.9 | - | 0/0 | 0 | - | 1 | 0/0/0 | 75 |  |
| 1193 | 24 | 55281 | 11.2+-4.9 | - | 0/0 | 0 | - | 0 | 0/0/0 | 69 |  |
| 1194 | 16 | 36853 | 11.9+-4.6 | - | 0/0 | 0 | - | 0 | 0/0/0 | 54 |  |
| 1195 | 17 | 40806 | 29.6+-48.7 | - | 0/0 | 0 | - | 1 | 0/0/0 | 44 |  |
| 1196 | 27 | 64933 | 13.1+-4.6 | - | 0/0 | 0 | - | 0 | 0/0/0 | 70 |  |
| 1197 | 19 | 45425 | 12.2+-5.1 | - | 0/0 | 0 | - | 1 | 0/0/0 | 49 |  |
| 1198 | 33 | 75776 | 14.1+-27.1 | - | 0/0 | 0 | - | 1 | 0/0/0 | 108 |  |
| 1199 | 21 | 51334 | 50.2+-142.3 | - | 0/0 | 0 | - | 1 | 0/0/0 | 62 |  |
| 1200 | 27 | 62963 | 38.3+-62.8 | - | 0/0 | 0 | - | 1 | 0/0/0 | 64 |  |
| 1201 | 5 | 11624 | 10.5+-1.2 | - | 0/0 | 0 | - | 0 | 0/0/0 | 10 |  |
| 1202 | 4 | 9477 | 17.0+-11.8 | - | 0/0 | 0 | - | 4 | 0/0/0 | 11 |  |
| 1203 | 20 | 47856 | 26.8+-28.6 | - | 0/0 | 0 | - | 0 | 0/0/0 | 51 |  |
| 1204 | 19 | 47206 | 44.0+-90.7 | - | 0/0 | 0 | - | 1 | 0/0/0 | 55 |  |
| 1205 | 11 | 26168 | 43.3+-58.0 | - | 0/0 | 0 | - | 0 | 0/0/0 | 30 |  |
| 1206 | 12 | 29309 | 17.7+-21.2 | - | 0/0 | 0 | - | 0 | 0/0/0 | 31 |  |
| 1207 | 3 | 6986 | 12.9+-4.1 | - | 0/0 | 0 | - | 0 | 0/0/0 | 9 |  |
| 1208 | 107 | 1103482 | 32.8+-22.1 | - | 0/0 | 0 | - | 15 | 0/0/0 | 1082 |  |
| 1209 | 28 | 67331 | 35.8+-81.1 | - | 0/0 | 0 | - | 0 | 0/0/0 | 86 |  |
| 1210 | 12 | 28548 | 26.5+-26.1 | - | 0/0 | 0 | - | 0 | 0/0/0 | 17 |  |
| 1211 | 18 | 42851 | 15.0+-8.0 | - | 0/0 | 0 | - | 0 | 0/0/0 | 67 |  |
| 1212 | 35 | 84883 | 21.8+-30.4 | - | 0/0 | 0 | - | 1 | 0/0/0 | 102 |  |
| 1213 | 22 | 52135 | 18.8+-25.5 | - | 0/0 | 0 | - | 0 | 0/0/0 | 48 |  |
| 1214 | 60 | 862919 | 14.1+-1.8 | - | 0/0 | 0 | - | 19 | 0/0/0 | 819 |  |
| 1215 | 22 | 53809 | 50.9+-153.3 | - | 0/0 | 0 | - | 2 | 0/0/1 | 55 |  |
| 1216 | 15 | 35601 | 34.2+-37.4 | - | 0/0 | 0 | - | 0 | 0/0/0 | 28 |  |
| 1217 | 18 | 42655 | 17.4+-33.8 | - | 0/0 | 0 | - | 2 | 0/0/0 | 53 |  |
| 1218 | 24 | 59122 | 22.7+-29.9 | - | 0/0 | 0 | - | 4 | 0/0/0 | 81 |  |
| 1219 | 18 | 44512 | 10.8+-2.3 | - | 0/0 | 0 | - | 0 | 0/0/0 | 46 |  |
| 1220 | 20 | 49805 | 19.1+-13.4 | - | 0/0 | 0 | - | 0 | 0/0/0 | 61 |  |
| 1221 | 18 | 44978 | 24.3+-32.0 | - | 0/0 | 0 | - | 0 | 0/0/0 | 55 |  |
| 1222 | 19 | 47280 | 13.1+-6.9 | - | 0/0 | 0 | - | 1 | 0/0/0 | 51 |  |
| 1223 | 8 | 19934 | 38.5+-41.6 | - | 0/0 | 0 | - | 0 | 0/0/0 | 21 |  |
| 1224 | 19 | 47316 | 29.0+-21.6 | - | 0/0 | 0 | - | 0 | 0/0/0 | 58 |  |
| 1225 | 25 | 61463 | 23.1+-24.5 | - | 0/0 | 0 | - | 0 | 0/0/0 | 74 |  |
| 1226 | 22 | 55315 | 17.3+-17.3 | - | 0/0 | 0 | - | 0 | 0/0/0 | 63 |  |
| 1227 | 167 | 1252795 | 14.0+-2.0 | - | 0/0 | 0 | - | 18 | 2/0/0 | 1050 |  |
| 1228 | 18 | 44121 | 60.3+-69.4 | - | 0/0 | 0 | - | 2 | 0/0/0 | 61 |  |
| 1229 | 20 | 48977 | 16.9+-11.1 | - | 0/0 | 0 | - | 0 | 0/0/0 | 31 |  |
| 1230 | 21 | 54130 | 22.8+-20.4 | - | 0/0 | 0 | - | 0 | 0/0/0 | 76 |  |
| 1231 | 22 | 56004 | 15.7+-9.7 | - | 0/0 | 0 | - | 1 | 0/0/0 | 84 |  |
| 1232 | 22 | 55205 | 53.0+-133.7 | - | 0/0 | 0 | - | 0 | 0/0/0 | 68 |  |
| 1233 | 20 | 48533 | 16.0+-21.5 | - | 0/0 | 0 | - | 0 | 0/0/0 | 56 |  |
| 1234 | 20 | 51197 | 16.8+-9.5 | - | 0/0 | 0 | - | 0 | 0/0/0 | 75 |  |
| 1235 | 24 | 59711 | 18.2+-18.5 | - | 0/0 | 0 | - | 0 | 0/0/0 | 75 |  |
| 1236 | 18 | 45360 | 46.2+-90.0 | - | 0/0 | 0 | - | 4 | 0/0/0 | 85 |  |
| 1237 | 31 | 78198 | 14.2+-9.4 | - | 0/0 | 0 | - | 0 | 0/0/0 | 101 |  |
| 1238 | 25 | 63182 | 15.5+-12.5 | - | 0/0 | 0 | - | 4 | 0/0/0 | 80 |  |
| 1239 | 26 | 65590 | 11.4+-5.5 | - | 0/0 | 0 | - | 0 | 0/0/0 | 88 |  |
| 1240 | 27 | 67336 | 14.0+-7.5 | - | 0/0 | 0 | - | 0 | 0/0/0 | 65 |  |
| 1241 | 25 | 64183 | 14.3+-9.7 | - | 0/0 | 0 | - | 2 | 0/0/0 | 74 |  |
| 1242 | 22 | 58477 | 16.2+-17.7 | - | 0/0 | 0 | - | 1 | 0/0/0 | 53 |  |
| 1243 | 12 | 31085 | 30.1+-36.1 | - | 0/0 | 0 | - | 0 | 0/0/0 | 25 |  |
| 1244 | 17 | 44918 | 15.4+-6.3 | - | 0/0 | 0 | - | 0 | 0/0/0 | 45 |  |
| 1245 | 22 | 58933 | 22.5+-26.7 | - | 0/0 | 0 | - | 0 | 0/0/0 | 73 |  |
| 1246 | 23 | 60373 | 18.6+-17.7 | - | 0/0 | 0 | - | 0 | 0/0/0 | 72 |  |
| 1247 | 26 | 67540 | 28.8+-35.8 | - | 0/0 | 0 | - | 0 | 1/0/0 | 92 |  |
| 1248 | 76 | 2299268 | 60.3+-61.1 | - | 0/0 | 0 | - | 28 | 3/0/0 | 1863 |  |
| 1249 | 14 | 33322 | 36.7+-32.8 | - | 0/0 | 0 | - | 2 | 0/0/0 | 36 |  |
| 1250 | 13 | 33395 | 14.5+-6.1 | - | 0/0 | 0 | - | 0 | 0/0/0 | 21 |  |
| 1251 | 19 | 50632 | 12.2+-4.3 | - | 0/0 | 0 | - | 0 | 0/0/0 | 61 |  |
| 1252 | 29 | 76858 | 14.1+-8.6 | - | 0/0 | 0 | - | 0 | 0/0/0 | 111 |  |
| 1253 | 114 | 1088923 | 33.3+-46.6 | - | 0/0 | 0 | - | 27 | 0/0/0 | 1102 |  |
| 1254 | 20 | 53121 | 17.8+-19.4 | - | 0/0 | 0 | - | 0 | 0/0/0 | 40 |  |
| 1255 | 13 | 31643 | 9.2+-1.6 | - | 0/0 | 0 | - | 0 | 0/0/0 | 46 |  |
| 1256 | 19 | 51581 | 45.3+-53.0 | - | 0/0 | 0 | - | 0 | 0/0/0 | 74 |  |
| 1257 | 34 | 89351 | 22.4+-27.1 | - | 0/0 | 0 | - | 0 | 0/0/0 | 114 |  |
| 1258 | 17 | 43251 | 17.4+-10.9 | - | 0/0 | 0 | - | 0 | 0/0/0 | 30 |  |
| 1259 | 17 | 44934 | 77.7+-119.1 | - | 0/0 | 0 | - | 0 | 0/0/0 | 58 |  |
| 1260 | 26 | 68888 | 28.8+-30.4 | - | 0/0 | 0 | - | 0 | 0/0/0 | 70 |  |
| 1261 | 26 | 70514 | 16.7+-6.9 | - | 0/0 | 0 | - | 1 | 0/0/0 | 94 |  |
| 1262 | 30 | 79850 | 16.1+-9.2 | - | 0/0 | 0 | - | 0 | 0/0/0 | 84 |  |
| 1263 | 27 | 71811 | 16.1+-7.0 | - | 0/0 | 0 | - | 0 | 0/0/0 | 83 |  |
| 1264 | 25 | 63037 | 14.9+-6.8 | - | 0/0 | 0 | - | 0 | 0/0/0 | 35 |  |
| 1265 | 25 | 67266 | 15.0+-12.9 | - | 0/0 | 0 | - | 1 | 0/0/0 | 97 |  |
| 1266 | 21 | 57957 | 19.0+-16.6 | - | 0/0 | 0 | - | 0 | 0/0/0 | 52 |  |
| 1267 | 27 | 73359 | 16.2+-12.5 | - | 0/0 | 0 | - | 0 | 0/0/0 | 99 |  |
| 1268 | 19 | 48481 | 10.5+-2.8 | - | 0/0 | 0 | - | 0 | 0/0/0 | 40 |  |
| 1269 | 17 | 42507 | 9.5+-2.9 | - | 0/0 | 0 | - | 0 | 0/0/0 | 49 |  |
| 1270 | 15 | 41698 | 27.8+-49.4 | - | 0/0 | 0 | - | 0 | 0/0/0 | 49 |  |
| 1271 | 38 | 99997 | 22.7+-27.3 | - | 0/0 | 0 | - | 1 | 0/0/0 | 113 |  |
| 1272 | 22 | 59646 | 12.2+-5.9 | - | 0/0 | 0 | - | 0 | 0/0/0 | 78 |  |
| 1273 | 21 | 56810 | 56.7+-48.9 | - | 0/0 | 0 | - | 1 | 0/0/0 | 67 |  |
| 1274 | 19 | 51821 | 11.3+-3.8 | - | 0/0 | 0 | - | 2 | 0/0/0 | 45 |  |
| 1275 | 27 | 75420 | 59.9+-100.7 | - | 0/0 | 0 | - | 0 | 0/0/0 | 94 |  |
| 1276 | 128 | 1933011 | 14.3+-1.6 | - | 0/0 | 0 | - | 26 | 0/0/0 | 1755 |  |
| 1277 | 24 | 65006 | 15.8+-11.6 | - | 0/0 | 0 | - | 0 | 0/0/0 | 76 |  |
| 1278 | 20 | 54024 | 18.0+-11.1 | - | 0/0 | 0 | - | 3 | 0/0/0 | 68 |  |
| 1279 | 20 | 53764 | 13.8+-7.4 | - | 0/0 | 0 | - | 0 | 0/0/0 | 46 |  |
| 1280 | 16 | 44092 | 18.7+-15.4 | - | 0/0 | 0 | - | 0 | 0/0/0 | 48 |  |
| 1281 | 27 | 71870 | 29.2+-47.5 | - | 0/0 | 0 | - | 0 | 0/0/0 | 86 |  |
| 1282 | 28 | 72879 | 25.4+-40.8 | - | 0/0 | 0 | - | 0 | 0/0/0 | 106 |  |
| 1283 | 52 | 636505 | 33.3+-24.3 | - | 0/0 | 0 | - | 11 | 0/0/0 | 645 |  |
| 1284 | 19 | 53522 | 16.8+-17.4 | - | 0/0 | 0 | - | 0 | 0/0/0 | 66 |  |
| 1285 | 30 | 76936 | 11.1+-2.4 | - | 0/0 | 0 | - | 1 | 0/0/0 | 97 |  |
| 1286 | 24 | 65321 | 15.1+-10.4 | - | 0/0 | 0 | - | 0 | 0/0/0 | 80 |  |
| 1287 | 17 | 45766 | 16.2+-13.8 | Thermoprotei | 1/1 | 1.0 | 0 | 0 | 0/0/0 | 40 |  |
| 1288 | 21 | 59041 | 17.2+-18.6 | - | 0/0 | 0 | - | 1 | 0/0/0 | 64 |  |
| 1289 | 20 | 58784 | 16.3+-7.2 | - | 0/0 | 0 | - | 2 | 1/0/0 | 71 |  |
| 1290 | 22 | 62936 | 12.3+-3.7 | - | 0/0 | 0 | - | 0 | 0/0/0 | 42 |  |
| 1291 | 7 | 18906 | 220.7+-118.4 | - | 0/0 | 0 | - | 0 | 0/0/0 | 23 |  |
| 1292 | 18 | 50935 | 11.8+-3.8 | - | 0/0 | 0 | - | 0 | 0/0/0 | 39 |  |
| 1293 | 33 | 87607 | 11.5+-6.4 | - | 0/0 | 0 | - | 0 | 0/0/0 | 106 |  |
| 1294 | 29 | 77294 | 11.4+-3.3 | - | 0/0 | 0 | - | 0 | 0/0/0 | 131 |  |
| 1295 | 3 | 132252 | 41.4+-4.6 | - | 0/0 | 0 | - | 2 | 0/0/0 | 177 |  |
| 1296 | 18 | 52076 | 24.7+-14.7 | - | 0/0 | 0 | - | 2 | 0/0/0 | 46 |  |
| 1297 | 21 | 60322 | 35.0+-46.4 | - | 0/0 | 0 | - | 0 | 0/0/0 | 76 |  |
| 1298 | 24 | 68789 | 43.3+-55.0 | - | 0/0 | 0 | - | 0 | 0/0/0 | 52 |  |
| 1299 | 36 | 100029 | 19.7+-15.4 | - | 0/0 | 0 | - | 0 | 0/0/0 | 120 |  |
| 1300 | 37 | 96915 | 12.4+-5.6 | - | 0/0 | 0 | - | 0 | 0/0/0 | 104 |  |
| 1301 | 32 | 90612 | 31.5+-45.9 | - | 0/0 | 0 | - | 0 | 0/0/0 | 127 |  |
| 1302 | 28 | 80826 | 13.2+-5.8 | - | 0/0 | 0 | - | 0 | 0/0/0 | 91 |  |
| 1303 | 20 | 58347 | 20.9+-14.4 | - | 0/0 | 0 | - | 3 | 2/0/0 | 88 |  |
| 1304 | 19 | 56176 | 17.5+-16.2 | - | 0/0 | 0 | - | 0 | 0/0/0 | 61 |  |
| 1305 | 30 | 79041 | 43.8+-40.3 | - | 0/0 | 0 | - | 2 | 0/0/0 | 68 |  |
| 1306 | 25 | 195896 | 33.0+-16.5 | - | 0/0 | 0 | - | 1 | 0/0/0 | 264 |  |
| 1307 | 21 | 58914 | 11.3+-3.7 | - | 0/0 | 0 | - | 2 | 0/0/0 | 67 |  |
| 1308 | 24 | 65615 | 22.8+-29.2 | - | 0/0 | 0 | - | 1 | 0/0/0 | 73 |  |
| 1309 | 31 | 89794 | 46.1+-123.7 | - | 0/0 | 0 | - | 0 | 0/0/0 | 115 |  |
| 1310 | 23 | 67479 | 20.4+-27.1 | - | 0/0 | 0 | - | 1 | 0/0/0 | 80 |  |
| 1311 | 9 | 173834 | 158.7+-160.7 | - | 0/0 | 0 | - | 4 | 0/0/0 | 219 |  |
| 1312 | 10 | 30176 | 27.8+-33.4 | - | 0/0 | 0 | - | 0 | 0/0/0 | 28 |  |
| 1313 | 14 | 38794 | 39.8+-43.5 | - | 0/0 | 0 | - | 0 | 0/0/0 | 29 |  |
| 1314 | 20 | 58984 | 52.4+-89.4 | - | 0/0 | 0 | - | 0 | 0/0/0 | 71 |  |
| 1315 | 27 | 76152 | 30.5+-41.8 | - | 0/0 | 0 | - | 0 | 0/0/0 | 79 |  |
| 1316 | 25 | 74029 | 16.0+-6.1 | - | 0/0 | 0 | - | 0 | 0/0/0 | 76 |  |
| 1317 | 16 | 47841 | 18.2+-14.2 | - | 0/0 | 0 | - | 1 | 0/0/0 | 55 |  |
| 1318 | 17 | 49323 | 16.7+-19.4 | - | 0/0 | 0 | - | 0 | 0/0/0 | 65 |  |
| 1319 | 28 | 77091 | 18.0+-18.4 | Thermoprotei | 1/1 | 1.0 | 0 | 0 | 0/0/0 | 97 |  |
| 1320 | 19 | 56993 | 14.0+-7.6 | - | 0/0 | 0 | - | 0 | 0/0/0 | 49 |  |
| 1321 | 15 | 40542 | 29.5+-21.2 | - | 0/0 | 0 | - | 0 | 0/0/0 | 50 |  |
| 1322 | 32 | 86783 | 21.7+-30.7 | - | 0/0 | 0 | - | 0 | 0/0/0 | 92 |  |
| 1323 | 29 | 81752 | 25.8+-27.2 | - | 0/0 | 0 | - | 0 | 0/0/0 | 96 |  |
| 1324 | 16 | 46789 | 12.3+-4.5 | - | 0/0 | 0 | - | 0 | 0/0/0 | 49 |  |
| 1325 | 17 | 51369 | 27.9+-29.1 | - | 0/0 | 0 | - | 0 | 0/0/0 | 51 |  |
| 1326 | 48 | 130349 | 15.2+-6.6 | - | 0/0 | 0 | - | 0 | 0/0/0 | 125 |  |
| 1327 | 21 | 65095 | 20.0+-16.9 | - | 0/0 | 0 | - | 1 | 0/0/0 | 93 |  |
| 1328 | 27 | 71553 | 13.5+-9.7 | - | 0/0 | 0 | - | 0 | 0/0/0 | 80 |  |
| 1329 | 51 | 134713 | 10.0+-1.8 | - | 0/0 | 0 | - | 2 | 0/0/0 | 147 |  |
| 1330 | 11 | 32184 | 29.0+-44.0 | - | 0/0 | 0 | - | 0 | 0/0/0 | 32 |  |
| 1331 | 36 | 98953 | 13.0+-10.8 | - | 0/0 | 0 | - | 0 | 0/0/0 | 91 |  |
| 1332 | 13 | 38373 | 14.0+-6.3 | - | 0/0 | 0 | - | 0 | 0/0/0 | 28 |  |
| 1333 | 36 | 103927 | 18.9+-9.6 | - | 0/0 | 0 | - | 0 | 0/0/0 | 147 |  |
| 1334 | 23 | 72984 | 18.6+-12.0 | - | 0/0 | 0 | - | 2 | 0/0/0 | 70 |  |
| 1335 | 18 | 54383 | 30.8+-44.4 | - | 0/0 | 0 | - | 0 | 0/0/0 | 53 |  |
| 1336 | 20 | 61989 | 19.1+-15.9 | - | 0/0 | 0 | - | 0 | 0/0/0 | 78 |  |
| 1337 | 21 | 67657 | 20.9+-39.2 | - | 0/0 | 0 | - | 2 | 0/0/0 | 81 |  |
| 1338 | 31 | 87392 | 16.8+-17.3 | - | 0/0 | 0 | - | 0 | 0/0/0 | 105 |  |
| 1339 | 20 | 61734 | 20.3+-17.8 | - | 0/0 | 0 | - | 0 | 0/0/0 | 33 |  |
| 1340 | 13 | 35704 | 27.4+-40.3 | - | 0/0 | 0 | - | 0 | 0/0/0 | 43 |  |
| 1341 | 15 | 44836 | 30.7+-35.9 | - | 0/0 | 0 | - | 0 | 0/0/0 | 44 |  |
| 1342 | 28 | 80905 | 18.0+-29.4 | - | 0/0 | 0 | - | 0 | 0/0/0 | 83 |  |
| 1343 | 30 | 90560 | 16.9+-11.9 | - | 0/0 | 0 | - | 3 | 1/0/0 | 118 |  |
| 1344 | 15 | 45168 | 16.7+-4.4 | - | 0/0 | 0 | - | 1 | 0/0/0 | 46 |  |
| 1345 | 23 | 72116 | 22.4+-22.4 | - | 0/0 | 0 | - | 0 | 0/0/0 | 89 |  |
| 1346 | 25 | 78075 | 20.3+-18.1 | - | 0/0 | 0 | - | 4 | 1/0/0 | 78 |  |
| 1347 | 16 | 46953 | 49.6+-65.3 | - | 0/0 | 0 | - | 0 | 0/0/0 | 40 |  |
| 1348 | 24 | 78744 | 25.3+-24.6 | - | 0/0 | 0 | - | 0 | 0/0/0 | 97 |  |
| 1349 | 11 | 33677 | 12.4+-6.4 | - | 0/0 | 0 | - | 0 | 0/0/0 | 27 |  |
| 1350 | 21 | 66739 | 12.2+-2.9 | - | 0/0 | 0 | - | 0 | 0/0/0 | 80 |  |
| 1351 | 17 | 49001 | 16.7+-13.1 | - | 0/0 | 0 | - | 0 | 0/0/0 | 29 |  |
| 1352 | 17 | 47727 | 13.6+-6.5 | - | 0/0 | 0 | - | 0 | 0/0/0 | 28 |  |
| 1353 | 27 | 85344 | 24.2+-26.5 | - | 0/0 | 0 | - | 0 | 1/0/0 | 111 |  |
| 1354 | 17 | 55381 | 17.3+-18.4 | - | 0/0 | 0 | - | 0 | 0/0/0 | 56 |  |
| 1355 | 17 | 50415 | 12.4+-4.3 | - | 0/0 | 0 | - | 0 | 0/0/0 | 53 |  |
| 1356 | 16 | 479796 | 111.7+-21.3 | - | 0/0 | 0 | - | 9 | 0/0/0 | 430 |  |
| 1357 | 34 | 98886 | 19.3+-24.4 | - | 0/0 | 0 | - | 0 | 0/0/0 | 126 |  |
| 1358 | 21 | 63498 | 14.9+-12.3 | - | 0/0 | 0 | - | 0 | 0/0/0 | 61 |  |
| 1359 | 36 | 110469 | 25.3+-24.0 | - | 0/0 | 0 | - | 6 | 0/0/0 | 145 |  |
| 1360 | 20 | 59946 | 36.7+-20.4 | - | 0/0 | 0 | - | 1 | 0/0/0 | 58 |  |
| 1361 | 56 | 165128 | 26.3+-28.3 | - | 0/0 | 0 | - | 0 | 0/0/0 | 214 |  |
| 1362 | 20 | 62887 | 20.4+-28.4 | - | 0/0 | 0 | - | 0 | 0/0/0 | 75 |  |
| 1363 | 20 | 62747 | 12.1+-8.5 | - | 0/0 | 0 | - | 0 | 0/0/0 | 94 |  |
| 1364 | 14 | 40735 | 76.6+-113.8 | - | 0/0 | 0 | - | 1 | 0/0/0 | 60 |  |
| 1365 | 25 | 78553 | 17.9+-20.8 | - | 0/0 | 0 | - | 0 | 0/0/0 | 75 |  |
| 1366 | 30 | 89838 | 15.8+-9.4 | - | 0/0 | 0 | - | 1 | 0/0/0 | 137 |  |
| 1367 | 28 | 83509 | 17.8+-13.6 | - | 0/0 | 0 | - | 8 | 0/0/0 | 70 |  |
| 1368 | 28 | 84375 | 13.9+-12.1 | - | 0/0 | 0 | - | 0 | 0/0/0 | 112 |  |
| 1369 | 30 | 95691 | 16.6+-9.2 | - | 0/0 | 0 | - | 1 | 0/0/0 | 109 |  |
| 1370 | 14 | 46541 | 28.5+-27.0 | - | 0/0 | 0 | - | 0 | 0/0/0 | 26 |  |
| 1371 | 30 | 92392 | 23.8+-34.5 | - | 0/0 | 0 | - | 0 | 0/0/0 | 113 |  |
| 1372 | 23 | 72164 | 20.7+-14.7 | - | 0/0 | 0 | - | 2 | 0/0/0 | 78 |  |
| 1373 | 19 | 64425 | 25.4+-23.2 | - | 0/0 | 0 | - | 0 | 2/0/0 | 70 |  |
| 1374 | 15 | 44234 | 46.2+-101.1 | - | 0/0 | 0 | - | 0 | 0/0/0 | 58 |  |
| 1375 | 19 | 65415 | 42.1+-37.5 | - | 0/0 | 0 | - | 0 | 0/0/0 | 75 |  |
| 1376 | 8 | 25826 | 35.0+-51.8 | - | 0/0 | 0 | - | 1 | 0/0/0 | 28 |  |
| 1377 | 20 | 64540 | 15.1+-10.2 | - | 0/0 | 0 | - | 0 | 0/0/0 | 70 |  |
| 1378 | 26 | 86400 | 19.9+-15.2 | - | 0/0 | 0 | - | 2 | 0/0/0 | 126 |  |
| 1379 | 20 | 61155 | 12.0+-5.0 | - | 0/0 | 0 | - | 0 | 0/0/0 | 47 |  |
| 1380 | 17 | 58279 | 17.0+-12.5 | - | 0/0 | 0 | - | 0 | 0/0/0 | 34 |  |
| 1381 | 11 | 218801 | 1176.7+-290.1 | - | 0/0 | 0 | - | 0 | 0/0/0 | 291 |  |
| 1382 | 23 | 76493 | 25.5+-30.7 | - | 0/0 | 0 | - | 0 | 0/0/0 | 117 |  |
| 1383 | 44 | 120960 | 11.2+-4.1 | - | 0/0 | 0 | - | 1 | 0/0/0 | 146 |  |
| 1384 | 3 | 8493 | 7.5+-1.1 | - | 0/0 | 0 | - | 0 | 0/0/0 | 10 |  |
| 1385 | 22 | 63836 | 18.0+-21.3 | - | 0/0 | 0 | - | 0 | 0/0/0 | 74 |  |
| 1386 | 7 | 164023 | 36.1+-11.9 | - | 0/0 | 0 | - | 1 | 0/0/0 | 183 |  |
| 1387 | 47 | 139135 | 15.2+-10.5 | Thermoprotei | 2/2 | 2.0 | 0 | 3 | 1/0/0 | 185 |  |
| 1388 | 18 | 63243 | 20.1+-17.7 | Methanosarcinales | 1/1 | 1.0 | 0 | 1 | 0/0/0 | 73 |  |
| 1389 | 24 | 81027 | 24.2+-35.3 | - | 0/0 | 0 | - | 0 | 0/0/0 | 75 |  |
| 1390 | 37 | 123525 | 17.1+-9.5 | - | 0/0 | 0 | - | 1 | 0/0/0 | 151 |  |
| 1391 | 29 | 96307 | 34.0+-48.2 | - | 0/0 | 0 | - | 1 | 0/0/0 | 123 |  |
| 1392 | 48 | 144935 | 21.9+-16.7 | - | 0/0 | 0 | - | 0 | 0/0/0 | 194 |  |
| 1393 | 29 | 95492 | 16.8+-6.8 | - | 0/0 | 0 | - | 0 | 0/0/0 | 65 |  |
| 1394 | 20 | 71145 | 13.7+-14.3 | - | 0/0 | 0 | - | 0 | 0/0/0 | 82 |  |
| 1395 | 19 | 63020 | 20.8+-26.3 | - | 0/0 | 0 | - | 0 | 0/0/0 | 68 |  |
| 1396 | 38 | 118940 | 16.4+-14.1 | - | 0/0 | 0 | - | 2 | 0/0/0 | 155 |  |
| 1397 | 38 | 123809 | 50.1+-70.2 | - | 0/0 | 0 | - | 0 | 0/0/0 | 131 |  |
| 1398 | 18 | 62156 | 23.0+-28.1 | - | 0/0 | 0 | - | 1 | 0/0/0 | 73 |  |
| 1399 | 17 | 281416 | 610.8+-239.8 | - | 0/0 | 0 | - | 0 | 0/0/0 | 343 |  |
| 1400 | 23 | 83814 | 21.3+-17.9 | - | 0/0 | 0 | - | 0 | 0/0/0 | 114 |  |
| 1401 | 15 | 49716 | 96.9+-300.0 | - | 0/0 | 0 | - | 0 | 0/0/0 | 36 |  |
