# Supplementary material for: A Comparative Metagenome Survey of the Fecal Microbiota of a Breast- and a Plant-Fed Asian Elephant Reveals an Unexpectedly High Diversity of Glycoside Hydrolase Family Enzymes
Source: PLoS One. 2014 Sep 10;9(9):e106707. doi: 10.1371/journal.pone.0106707 (PMC4160196; doi:10.1371/journal.pone.0106707)
Supplement: Table S4 — OTUs observed in the 16S rRNA gene datasets of the six-years-old and the three-weeks-old elephant and their frequencies. (DOCX) [file pone.0106707.s005.docx]

**TABLE S4:** OTUs observed in the 16S rRNA gene datasets of the six-years-old and the three-weeks-old elephant and their frequencies.

| **Amount of sequences assigned to the respective OTU** | | **Phylogenetic assignment (best observed)** |
| --- | --- | --- |
| **Six-years-old elephant** | **Three-weeks-old elephant** |  |
| 4 | 16 | Oscillospira |
| 39 | 36 | Ruminococcaceae |
| 1 | 1 | Ruminococcaceae |
| 4 | 4 | Ruminococcaceae |
| 114 | 10 | Ruminococcaceae |
| 13 | 71 | Clostridiaceae |
| 6 | 5 | Streptococcus |
| 467 | 2 | Paraprevotellaceae |
| 237 | 2 | Bacteroidales |
| 233 | 2 | Bacteroidales |
| 1 | 18 | Comamonadaceae |
| 3 | 1909 | Acinetobacter |
| 47 | 495 | Enterobacteriaceae |
