# Supplementary material for: A Comparative Metagenome Survey of the Fecal Microbiota of a Breast- and a Plant-Fed Asian Elephant Reveals an Unexpectedly High Diversity of Glycoside Hydrolase Family Enzymes
Source: PLoS One. 2014 Sep 10;9(9):e106707. doi: 10.1371/journal.pone.0106707 (PMC4160196; doi:10.1371/journal.pone.0106707)
Supplement: Supporting Information S1 — 16S rRNA gene analysis of the baby elephant. (HTML) [file pone.0106707.s006.html]

Javascript must be enabled to view this page.

members
magnitude

young\_assam\_complete---ssu---krona---Elephant\_Assam\_complete----Total---sim\_93---tax\_silva---td\_20

45508

45495

292

265

265

265

8

4

2

251

27

1

1

1

4

4

4

22

22

22

14403

11

1

1

1

10

10

10

14360

142

142

8

126

8

3

1

1

2

2

497

497

2

495

1

1

1

13717

5656

5656

8061

5291

2770

32

32

14

5

1

2

6

18

18

25765

153

153

153

29

124

15643

15643

1

3054

2636

418

6585

880

2

4

359

511

4

4359

4359

764

2

762

9969

9969

9969

132

9828

5

4

2

2

2

2

2

2

2

2

2

2

170

170

170

170

170

4861

4178

4178

818

556

7

23

2

24

14

175

17

2

2

2

7

7

4

4

828

1

827

2279

2

436

2

4

1835

71

71

2

2

165

117

46

2

27

25

7

7

6

6

12

12

2

2

2

625

625

111

9

102

514

119

395

31

31

31

31

13
