# Supplementary material for: A Comparative Metagenome Survey of the Fecal Microbiota of a Breast- and a Plant-Fed Asian Elephant Reveals an Unexpectedly High Diversity of Glycoside Hydrolase Family Enzymes
Source: PLoS One. 2014 Sep 10;9(9):e106707. doi: 10.1371/journal.pone.0106707 (PMC4160196; doi:10.1371/journal.pone.0106707)
Supplement: Supporting Information S2 — 16S rRNA gene analysis of the six-years-old elephant. (HTML) [file pone.0106707.s007.html]

Javascript must be enabled to view this page.

members
magnitude

kandy\_only\_mid3---ssu---krona---Elephant\_Candy\_MID3----Total---sim\_93---tax\_silva---td\_20

38439

38346

46

45

45

45

41

1

3

1

1

1

181

8

8

8

8

21

20

20

4

16

1

1

1

108

1

51

51

4

27

20

49

49

2

45

2

7

7

7

42

42

13

13

28

1

1

2

837

837

837

837

837

18736

18726

18726

1

206

3253

1196

5528

117

5130

106

17

15

140

3

1543

1290

152

101

1888

72

72

5039

4212

827

4

4

4

4

6

1671

1671

1671

1671

1444

227

33

260

2

258

258

258

231

27

250

1884

1884

1795

1795

1170

625

89

179

179

179

179

179

14003

12139

12139

4464

3851

3

40

10

281

44

5

119

111

285

1

1

21

19

2

17

12

5

350

12

338

5441

45

3714

22

110

51

410

88

74

571

2

37

7

2

306

1508

7

549

70

882

52

52

55

55

49

28

21

6

6

805

805

713

713

92

86

6

1004

1004

1004

70

3

924

7

8

2

2

6

6

6

6

103

27

76

101

101

4

46

46

46

30

30

30

21

52

52

52

2

2

2

61

32

32

30

30

30

30

2

2

2

2
